# Cell-based regenerative interventions in Parkinson's disease: Overcoming pharmacological limitations

**DOI:** 10.1016/j.apsb.2026.03.031

**Published:** 2026-03-20

**Authors:** Ana Lizeth Padilla, José L. Lanciego, Miquel Vila, Fernando de Castro, David Pozo, Fernando Rodríguez de Fonseca, Antonia Serrano, Javier Sáez-Valero, Lucía Núñez, Carlos Villalobos, Victor Tapias

**Affiliations:** a Excellence Unit Institute of Biomedicine and Molecular Genetics of Valladolid (IBGM), University of Valladolid & Consejo Superior de Investigaciones Científicas (CSIC), Valladolid 47003, Spain; b Department of Biochemistry and Molecular Biology and Physiology, Medical School, University of Valladolid, Valladolid 47005, Spain; c CNS Gene Therapy Program, Center for Applied Medical Research (CIMA) University of Navarra, Pamplona 31008, Spain; d Centro de Investigación Biomédica en Red de Enfermedades Neurodegenerativas (CIBERNED), Madrid 28029, Spain; e Aligning Science Across Parkinson's (ASAP) Collaborative Research Network, Chevy Chase, MD 20815, USA; f Neurodegenerative Diseases Research Group, Vall d'Hebron Research Institute (VHIR)-Center for Networked Biomedical Research on Neurodegenerative Diseases (CIBERNED), Barcelona 08035, Spain; g Department of Biochemistry and Molecular Biology, Institute of Neurosciences (INc-UAB), Autonomous University of Barcelona, Barcelona 08193, Spain; h Catalan Institution for Research and Advanced Studies (ICREA), Barcelona 08010, Spain; i Instituto Cajal, Consejo Superior de Investigaciones Científicas (CSIC), Madrid 28002, Spain; j Instituto de Investigación Biomédica de Málaga y Plataforma en Nanomedicina (IBIMA-Plataforma BIONAND), Málaga 29590, Spain; k Unidad Clínica de Neurología, Hospital Regional Universitario de Málaga, Málaga 29010, Spain; l Andalusian Network for Clinical and Translational Research in Neurology (NEURO-RECA), Málaga 29001, Spain; m Unidad de Gestión Clínica de Salud Mental, Hospital Regional Universitario de Málaga, Málaga 29010, Spain; n Centro Andaluz de Biología Molecular y Medicina Regenerativa (CABIMER), Universidad de Sevilla-Consejo Superior de Investigaciones Científicas, Sevilla 41092, Spain; o Department of Medical Biochemistry, Molecular Biology and Immunology, Medical School, University of Seville, Sevilla 41009, Spain; p Instituto de Neurociencias de Alicante, Universidad Miguel Hernández-CSIC, San Juan de Alicante, Alicante 03550, Spain; q Instituto de Investigación Sanitaria y Biomédica de Alicante (ISABIAL), Alicante 03010, Spain

**Keywords:** Parkinson's disease, Dopamine neurons, Cell-based therapy, Neuroprotection, Regeneration, Stem cells, Transplantation, Mitochondrial dysfunction

## Abstract

Parkinson's disease (PD) is a progressive neurodegenerative disorder characterized by the loss of dopaminergic (DA) neurons and the pathological aggregation of *α*-synuclein. While current pharmacological therapies provide symptomatic relief, they do not halt or reverse disease progression. Cell-based regenerative strategies have emerged as promising approaches to restore DA function and target the complex, multifactorial pathophysiology of PD. This review critically examines current approaches, including transplantation of fetal ventral mesencephalic tissue, pluripotent stem cell-derived midbrain DA progenitors, and *in vivo* reprogramming of endogenous cells. In addition, supportive cell types, such as mesenchymal stromal cells and carotid body glomus cells, provide neuroprotective and immunomodulatory effects *via* paracrine signaling. We summarize preclinical and clinical evidence on graft survival, integration, and functional recovery, and discuss key determinants of therapeutic efficacy, including mitochondrial function and bioenergetic integrity, immune compatibility, and biomaterial scaffolding. Despite significant progress, major challenges remain regarding long-term efficacy, graft standardization, and host–graft interactions. Ongoing translational advances are poised to drive the development of disease-modifying cell therapies capable of delivering durable clinical benefits and improving long-term outcomes in PD.

## Graphical abstract

Cell-based therapies for Parkinson's disease (PD) hold promise to restore dopaminergic (DA) function, provide robust neuroprotection, and modulate immunity, yet long-term graft integration, standardization, and durable disease modification remain key challenges.

**Figure:**
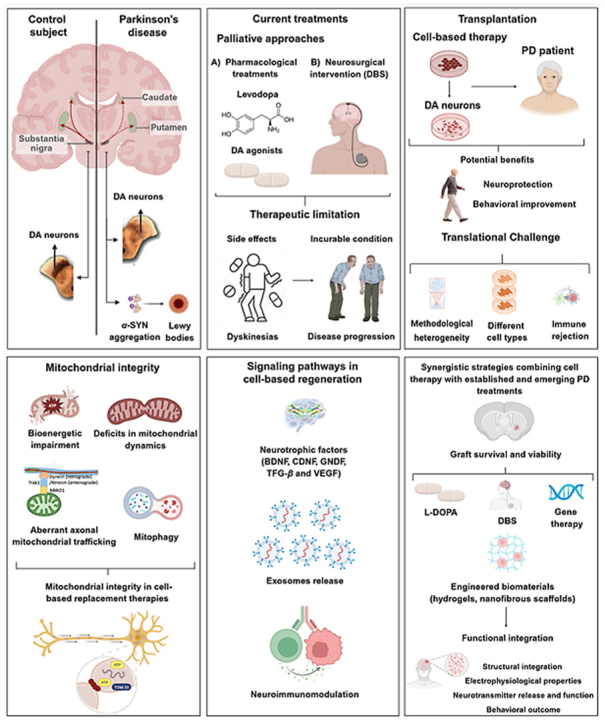

## 1 Introduction

**Figure 1:**
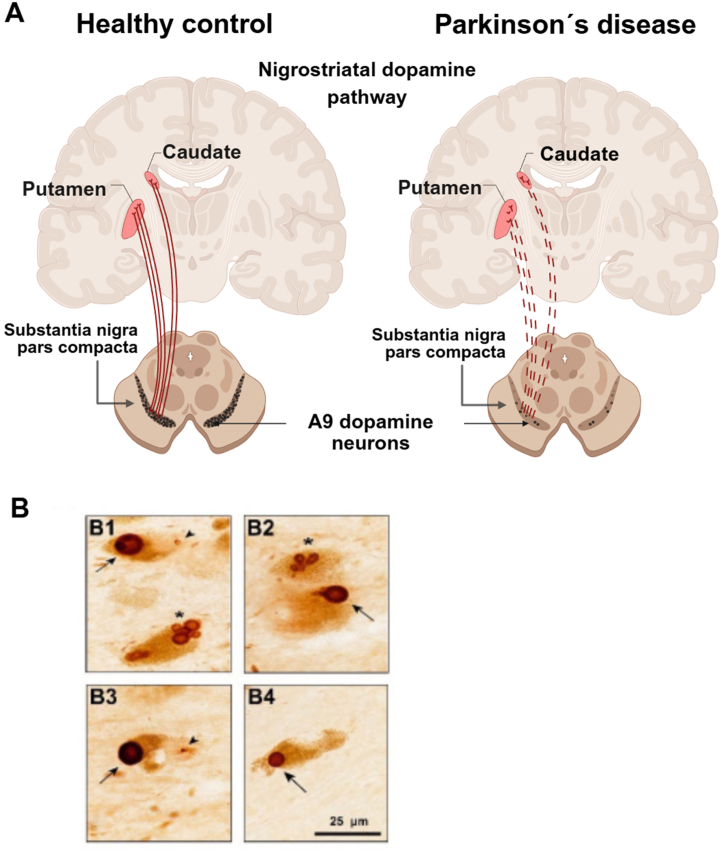
Nigrostriatal dopaminergic degeneration and the formation of Lewy bodies and Marinesco bodies as core neuropathological hallmarks of Parkinson's disease. (A) Selective degeneration of A9 dopamine (DA) neurons in the substantia nigra (SN) pars compacta and loss of their striatal nerve terminals. (B) Post-mortem Parkinson's disease (PD) midbrain sections showing Lewy body (LB) pathology in pigmented SN neurons. LBs (arrows) appear as ring-shaped inclusions with an immunoreactive peripheral halo and a paler core. Multivesicular LBs, characterized by multiple inclusions within a single DA neuron, are indicated by stars. Small nuclear inclusions known as Marinesco bodies (arrowheads), considered markers of advanced aging and PD, are also visible. Scale bar = 25 μm. Illustration created by the authors for this article.

Parkinson's disease (PD) is a chronic, age-associated neurodegenerative disorder characterized by the progressive loss of A9 dopamine (DA)-producing neurons in the substantia nigra (SN)^1^. Neurodegeneration typically initiates distally, with retraction of axon terminals and concomitant reduction in DA content within the caudate-putamen (striatum) (Fig. 1A). This process is accompanied by pathological aggregation of *α*-synuclein (*α*-SYN) into Lewy bodies (LBs) and Lewy neurites (Fig. 1B, original illustration generated by the authors for this manuscript)^2^^,^^3^. These inclusions, often co-localized with ubiquitin and p62, disrupt neuronal homeostasis and accelerate neurodegeneration. Under physiological conditions, *α*-SYN regulates synaptic vesicle trafficking, neurotransmission, and mitochondrial ATP synthesis^4^, ^5^, ^6^. In contrast, *α*-SYN pathological aggregation, influenced by post-translational modifications^7^^,^^8^, spreads from limbic regions toward the neocortex^9^, thereby disturbing synaptic organization and proteostatic balance^3^^,^^10^, ^11^, ^12^. Nuclear inclusions known as Marinesco bodies, detected within DA neurons, are associated with reduced striatal DA markers and may further exacerbate to neuronal vulnerability (Fig. 1B)^13^.

**Figure 2:**
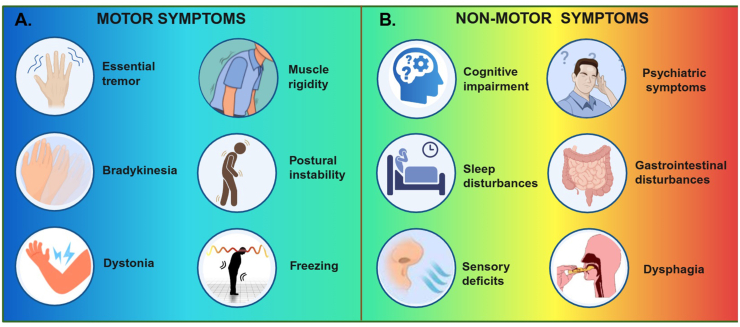
Common clinical signs of Parkinson's disease. (A) Motor symptoms encompass essential tremor, bradykinesia, dystonia, muscle rigidity, postural instability, and episodes of freezing of gait. These features reflect progressive loss of dopaminergic control over voluntary movement. (B) Non-motor symptoms include cognitive impairment, sleep disturbances, sensory abnormalities, mood disorders, and autonomic dysfunction, which often emerge early and substantially contribute to patient disability and reduced quality of life.

**Figure 3:**
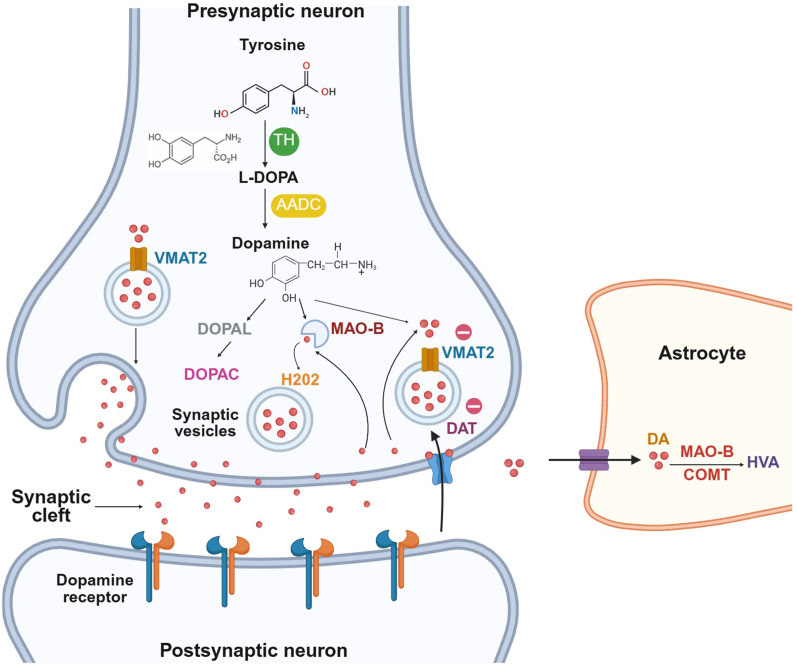
Dopamine synthesis, release, reuptake, and metabolism. Tyrosine is converted to l-3,4-dihydroxyphenylalanine (l-DOPA) by tyrosine hydroxylase (TH), and l-DOPA is then converted to dopamine (DA) by aromatic l-amino acid decarboxylase (AADC) in the presynaptic neuron. DA is loaded into synaptic vesicles by vesicular monoamine transporter 2 (VMAT2) and released into the synaptic cleft, where it binds to dopamine receptors on the postsynaptic neuron. Unbound DA is either returned to the presynaptic neuron through the dopamine transporter (DAT) or broken down by monoamine oxidase B (MAO-B) to 3,4-dihydroxyphenylacetaldehyde (DOPAL) and then 3,4-dihydroxyphenylacetic acid (DOPAC), producing hydrogen peroxide (H_2_O_2_). Astrocytes also clear DA, where MAO-B and catechol-*O*-methyltransferase (COMT) convert it to homovanillic acid (HVA).

Loss of striatal DA underlies the core motor symptoms of PD, including resting tremor, bradykinesia, rigidity, and postural instability (Fig. 2A)^14^^,^^15^, which arise from impaired basal ganglia circuits and higher-order cognitive processes^16^^,^^17^. In addition, patients exhibit a broad range of non-motor symptoms (Fig. 2B), including cognitive decline, mood disorders, sleep disturbances, autonomic and gastrointestinal dysfunction, and sensory deficits, all of which substantially reduce quality of life^18^, ^19^, ^20^, ^21^. DA metabolism is tightly controlled by tyrosine hydroxylase (TH), aromatic l-amino acid decarboxylase, vesicular monoamine transporter 2 (VMAT2), and the DA transporter (DAT) (Fig. 3)^22^. Early dysfunction of VMAT2 or DAT may occur before overt neuronal loss^23^, while oxidative metabolism of DA generates reactive oxygen species (ROS) and toxic aldehydes, promoting mitochondrial dysfunction and apoptosis^24^. Converging mechanisms, including neuroinflammation, glial activation, and impaired proteostasis further increase neuronal vulnerability, reflecting the multifactorial nature of PD pathogenesis^25^, ^26^, ^27^, ^28^.

Despite significant advances in pharmacological and surgical management, current therapeutic approaches, such as levodopa (l-DOPA), deep brain stimulation (DBS), and transcranial static magnetic stimulation (tSMS), remain largely symptomatic and do not alter the underlying neurodegenerative process. Although l-DOPA therapy is initially effective, chronic administration often leads to motor fluctuations and dyskinesias in up to half of patients within five years, offering limited benefit for cognitive and autonomic symptoms^29^, ^30^, ^31^, ^32^. DBS can alleviate selected motor deficits, but it is invasive, expensive, and fails to modify the underlying disease progression^33^^,^^34^. tSMS is a non-invasive neuromodulatory technique that transiently modulates motor cortex excitability^35^. In a randomized, sham-controlled trial in PD, repeated tSMS was safe but showed no significant objective reduction in l-DOPA-induced dyskinesias, despite moderate subjective improvement, reflecting its short-lived effects and the need for longer, controlled studies^36^. Therefore, there is an urgent need for therapies that restore lost DA function and alter disease trajectory. In this context, cell-based regenerative therapies have emerged as one of the most promising disease-modifying approaches for PD^37^. Preclinical studies in rodent and non-human primate models have demonstrated that transplanted DA progenitors can survive long-term, extend axons to appropriate striatal targets, and restore motor function^38^, ^39^, ^40^, ^41^, ^42^, ^43^, ^44^.

Early clinical trials using fetal ventral mesencephalic (fMV) tissue confirmed the feasibility of DA replacement in patients^45^, ^46^, ^47^, ^48^. More recently, first-in-human trials employing human induced pluripotent stem cell (hiPSC)-derived midbrain DA progenitors have demonstrated graft survival, local DA production, favorable safety profiles, and preliminary functional improvements at short-to mid-term follow-up^49^, ^50^, ^51^, marking a pivotal advance toward clinical translation. Parallel clinical investigations using mesenchymal stromal cells (MSCs) and carotid body (CB) glomus cells have demonstrated favorable safety and tolerability, with some patients showing modest yet measurable improvements in motor function, daily activities, and medication requirements, partially sustained for up to three years^52^, ^53^, ^54^, ^55^. Collectively, these results support the feasibility of autologous grafting strategies and justify further controlled studies to determine long-term efficacy in PD.

Recent advances in regenerative medicine now extend beyond simple neuronal replacement toward combinatorial strategies that integrate immune engineering, bioengineering, and metabolic resilience. Ongoing efforts include the generation of hypoimmunogenic iPSC lines, human leukocyte antigen (HLA)-edited donor cells, and regional haplobanks to mitigate immune rejection^56^^,^^57^, as well as interventions to prevent host-to-graft *α*-SYN propagation through the synuclein alpha gene (*SNCA*)-edited cells and *α*-SYN-targeted immunotherapies^58^, ^59^, ^60^, ^61^, ^62^. Innovations in biomaterials, such as biomimetic hydrogels, engineered scaffolds, and localized delivery systems, are further enhancing graft survival, axonal integration, and host tissue repair^42^^,^^63^, ^64^, ^65^, ^66^. In parallel, emerging strategies including astrocyte-to-neuron reprogramming^67^^,^^68^, extracellular vesicle-mediated neurorepair^69^^,^^70^, and approaches aimed at preserving mitochondrial function and bioenergetic resilience^71^^,^^72^, are expanding the therapeutic scope of regenerative interventions. Collectively, these developments mark a transition from proof-of-concept studies to multimodal, clinically scalable regenerative platforms that integrate optimized donor cells with immune, mitochondrial, and bioengineering innovations to achieve durable, disease-modifying benefits in PD.

This review provides a comprehensive overview of the mechanistic rationale, therapeutic progress, and translational challenges of cell-based interventions for PD, emphasizing their potential to improve both motor and non-motor outcomes. It further emphasizes emerging combinatorial strategies and the integration of regenerative medicine with precision bioengineering as a roadmap for next-generation disease-modifying therapies.

## 2 Axonal degeneration as an early pathological hallmark of Parkinson's disease

Axonal pathology precedes neuronal death, involving early degeneration that disrupts neuronal connectivity before the loss of the perikarya. This process is marked by progressive cytoskeletal disintegration, transport deficits, and synaptic dysfunction, ultimately causing axonal retraction and fragmentation, and contributing to the onset of both motor and non-motor symptoms in PD. The temporal progression of axon degeneration has been characterized in transected axon models^73^, revealing three phases: an acute degeneration phase affecting both proximal and distal segments immediately post-injury^74^^,^^75^, a latency period during which distal axons maintain structure and transient excitability^76^^,^^77^, and a rapid granular degeneration phase marked by swift cytoskeletal disintegration distal to the lesion^78^^,^^79^.

DA neurons exhibit a remarkably extensive and intricately structured axonal network, defined by a tightly packed and widely distributed branching pattern, enabling broad neuromodulatory regulation across multiple target regions. Morphological analyses in rats report total axonal lengths ranging from 1.4 × 10^5^ to 7.8 × 10^5^ μm for a single DA neuron in the dorsal tier of the SN, and 5 × 10^5^ to 6.1 × 10^5^ μm in the ventral tier ^80^. A single DA neuron in the rat SN forms approximately 100,000 to 250,000 synaptic connections, reflecting its highly branched architecture and extensive influence within the striatum^81^. These variations underscore the complex and region-specific arborization of DA neurons essential for their distinct functional roles in cognitive processing and motor control within basal ganglia circuitry.

Structural studies also show that the axon initial segment (the critical site for action potential initiation) in DA neurons of the mouse SN averages 25.8 μm in length, with an average of one synapse per neuron^82^. Due to challenges in direct human measurements, estimates of DA neuron axonal architecture derive from extrapolations of animal models and human anatomical data. Assuming conservation of fundamental organizational principles, a single SN DA neuron in humans is estimated to extend an axon approximately 4.5 m long and form between 1 and 2.4 million synapses^81^.

Distinct DA neuron populations display significant variations in intrinsic biophysical properties and input-output dynamics, reflecting functional heterogeneity within the nigrostriatal system^83^^,^^84^. However, all DA neuron subtypes share exceptionally high metabolic demands due to their extensive axonal arborization. To meet these demands, DA neurons are densely enriched with mitochondria and exhibit elevated oxidative phosphorylation, ensuring efficient ATP synthesis to support their complex activities^85^.

Axonal pathology typically originates at the distal end (axon terminal) and advances proximally in a “dying-back” pattern. Morphological alterations begin with swelling and microtubule disassembly, progressing to cytoskeletal fragmentation. DA subpopulations exhibit varying vulnerability to neurodegeneration. In PD, neurons within the ventrolateral SN are most susceptible^86^^,^^87^. This susceptibility likely reflects intrinsic and/or acquired susceptibility to *α*-SYN aggregation^88^. The loss of DA nerve terminals in the dorsal striatum exceeds the death of DA neurons in the SN and is characterized by disrupted structural integrity, concomitant alterations in DA metabolism, and dysregulated neurotransmitter release^89^.

Striatal DAT binding is a key indicator of DA terminal density and innervation integrity. DAT imaging with [^123^I]FP-CIT single-photon emission computed tomography is a well-established tool for differential diagnosis of PD and other neurodegenerative parkinsonism versus non-neurodegenerative tremor disorders^90^, ^91^, ^92^. The positron emission tomography (PET) radiotracer ^18^F-DOPA, a fluorinated form of l-DOPA, assesses presynaptic DA integrity and function in clinical and research settings^93^, ^94^, ^95^. Fluorodopa (^18^F-DOPA) uptake is also valuable for examining graft survival and function following neuronal transplantation^45^^,^^96^^,^^97^. Synaptic density can be measured using PET with the radioligand [^11^C]UCB-J, which binds to the synaptic vesicle glycoprotein 2A^98^. Advances in magnetic resonance imaging have further enhanced analysis of DA functional activity and denervation^99^, ^100^, ^101^.

Postmortem studies provide definitive evidence of progressive DA terminal degeneration coinciding with disease onset. DAT antibodies selectively detect DA projections in the striatum, while TH antibodies identify DA neurons, although expression of both markers varies with disease progression and regulatory mechanisms. Immunohistochemical analysis reveals a significant reduction (35%–75%) in TH and DAT signals in the dorsal putamen of PD subjects within 1–3 years post-diagnosis, worsening to 70%–90% by 5 years, followed by relative stabilization^87^.

Unbiased stereological quantification has revealed a substantial, yet variable, reduction (50%–90%) in TH-positive neurons in patients with PD compared to controls, a loss that is already apparent in the early stages of the disease. Subjects with shorter disease duration exhibit similar TH cell loss to those diagnosed over 20 years earlier, with only minimal additional loss (<20%) thereafter, leaving a residual neuronal population detectable decades post-diagnosis^87^. Quantitative analyses also demonstrate significant downregulation of TH and DAT at protein and mRNA levels in PD brain tissue relative to age-matched controls^102^, ^103^, ^104^, ^105^.

Collectively, these findings highlight axonal degeneration as a critical early event in PD, preceding neuronal death and driving synaptic dysfunction. The highly branched and metabolically demanding architecture of SN DA neurons renders them particularly vulnerable to degenerative stressors. Postmortem analyses and advanced imaging consistently demonstrate marked reductions in DA markers, underscoring the central role of axonal pathology in disease progression. The variability in neuronal susceptibility suggests both intrinsic factors (such as axonal architecture and metabolic burden) and extrinsic contributors (including *α*-SYN aggregation and neuroinflammation). Since axonal dysfunction precedes perikaryal loss, targeting axonal maintenance and synaptic integrity offers promising avenues for neuroprotection.

Importantly, this axon-first model reframes regenerative therapies, including cell-based interventions. Traditional strategies focus primarily on restoring lost neuronal somata within the SN, yet their success depends fundamentally on grafted DA neurons’ ability to develop extensive, functional axonal projections capable of reinnervating widespread targets, especially the dorsal striatum. The immense anatomical complexity and bioenergetic demands of human nigrostriatal DA neurons (requiring reconstruction of millions of synapses over meter-long axons) pose formidable challenges to regeneration.

Therefore, integrating knowledge of axonal degeneration dynamics and selective vulnerability into cell therapy design is essential. Future efforts must prioritize not only survival and phenotypic fidelity of grafted cells but also their capacity for axonal growth, target-specific synaptogenesis, and long-term integration into host circuits. Combining regenerative approaches with molecular strategies that enhance axonal resilience and outgrowth may significantly improve therapeutic outcomes in PD^106^. The following section examines cell-based therapies developed to address these anatomical and physiological demands, highlighting how advances in transplantation biology aim to meet the unique structural and metabolic challenges of the nigrostriatal system.

## 3 Neuroregenerative cell-based therapies for Parkinson's disease

Most current pharmacological and surgical interventions for PD primarily target motor symptoms but do not modify the underlying disease course. Their effectiveness declines over time as degeneration of the nigrostriatal system advances and the capacity for DA storage and release diminishes. l-DOPA, the cornerstone of PD therapy, provides robust symptomatic relief but is associated with long-term complications, including motor fluctuations, dyskinesias, and non-motor adverse effects such as hallucinations, fatigue, and cognitive impairment^32^. Disease severity and progression are commonly assessed using the Hoehn and Yahr (HY) scale and the Movement Disorder Society Unified Parkinson's Disease Rating Scale (MDS-UPDRS), which together evaluate motor and non-motor deficits throughout the disease course^107^, ^108^, ^109^. l-DOPA-induced dyskinesias result from complex alterations in neurotransmitter systems, including glutamate, serotonin, and GABA, combined with dysregulated intracellular signaling and gene expression^110^. These limitations emphasize the critical need for interventions that restore DA circuitry and neuronal function rather than simply relieving symptoms.

**Figure 4:**
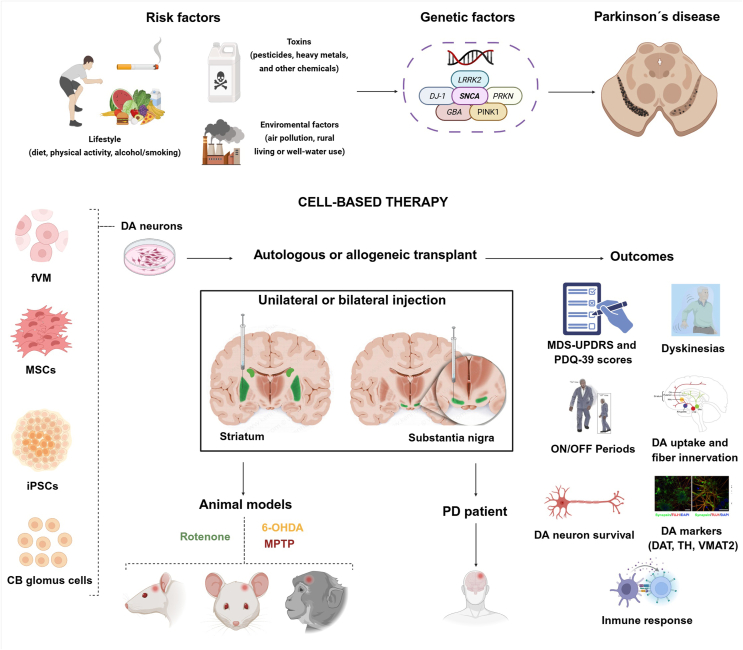
Cell-based regenerative interventions in Parkinson's disease. Parkinson's disease (PD) is influenced by lifestyle factors, environmental toxins, and genetic mutations, including *SNCA*, *LRRK2*, *PRKN*, *PINK1*, *DJ-1*, and *GBA*. Cell-based therapies aim to restore dopamine (DA) signaling using grafts derived from fetal ventral midbrain (fVM), human induced pluripotent stem cell (hiPSC)-derived DA neurons, mesenchymal stromal cells (MSCs), or carotid body (CB) glomus cells. These cells can be transplanted autologously or allogeneically into the striatum/caudate-putamen or substantia nigra. Their efficacy is evaluated in preclinical models and clinical trials, with outcomes including improved motor function, reduced ON/OFF periods, enhanced DA signaling, graft survival, and immune modulation.

Cell-based regenerative therapies have arisen as a promising strategy to address this unmet clinical need. These approaches aim to replace lost DA neurons, restore striatal innervation, improve motor and non-motor functions, and modulate immune and inflammatory responses. Advances in stem cell biology and cellular reprogramming have expanded the repertoire of transplantable DA neurons, enabling the potential for patient-specific regenerative interventions ^111^. Multiple cell types are under investigation, including fetal midbrain (fVM) tissue, iPSCs, MSCs, and CB glomus cells. Each cell source exhibits distinct neurorestorative potential, immunogenic profiles, and translational scalability (Fig. 4). The following subsections describe these strategies and the rationale for their selection in PD therapy.

### 3.1 Embryonic ventral mesencephalic tissue grafts

fVM tissue has long been explored as a therapeutic approach in PD due to its high content of A9 DA neurons, the subtype most affected in PD. These neurons can endogenously synthesize and release DA, extend axons over long distances, and integrate into host circuitry to support motor recovery. The fVM includes DA neurons from the retrorubral field (A8), SN pars compacta (A9), and ventral tegmental area (A10), each contributing distinct physiological roles and projection patterns^112^.

Successful transplantation depends on graft survival, axonal outgrowth, host integration, and restoration of DA-mediated neurotransmission. Preclinical studies in 6-hydroxydopamine (6-OHDA)-lesioned rodents^113^, ^114^, ^115^, ^116^, ^117^ and 1-methyl-4-phenyl-1,2,3,6-tetrahydropyridine (MPTP)-injected non-human primates^118^, ^119^, ^120^ demonstrated that fVM grafts can survive, reinnervate the host brain, and restore motor function. In humans, unilateral fVM transplantation into the putamen resulted in increased DA synthesis and improvements in rigidity, bradykinesia, and motor fluctuations^97^^,^^121^, ^122^, ^123^. Table 1^46^, ^47^, ^48^^,^^97^^,^^113^, ^114^, ^115^, ^116^, ^117^, ^118^, ^119^, ^120^, ^121^, ^122^, ^123^, ^124^, ^125^, ^126^, ^127^, ^128^ provides an overview of the key preclinical and clinical findings discussed above.

**Table 1:** FVM transplantation.

Species	Animal model clinical stage	Transplantation site	Injection	Behavioral phenotype	Nigrostriatal and histological outcomes	Lewy body pathology	Ref.
Rat	6-OHDA	Dorsal neostriatum	Unilateral	Amphetamine-induced rotation: ↓ rotational bias toward lesioned side (improved) Spontaneous rotation: ↑ turning contralateral to transplant Sensorimotor orientation: ↑ responsiveness contralateral to transplant Sensorimotor asymmetry: ↓ asymmetry in T-maze and sensory attention	Not evaluated	Not assessed	[113]
Rat	6-OHDA	2 graft sites: *Dorsal striatum* or *N. accumbens* prefrontal cortex or hippocampus	Unilateral	Striatal grafts restored rotations *N. accumbens* grafts worsened bias *P. cortex* or hippocampal grafts had no effect	Total neuron survival was similar across sites, but younger (E12) donor tissue and striatal sites: ↑ Number TH + neurons ↑ DA density ↑ A9/A10-like phenotypic differentiation	Not assessed	[114]
Rat	6-OHDA	Multiple striatal grafts A single SN graft	Unilateral	Multiple striatal grafts restored rotations more effectively than single SN grafts, showing better functional recovery	↑ Striatal TH fiber density ↑ TH + neurons	Not assessed	[115]
Rat	6-OHDA	Caudate-putamen	Unilateral	Not performed	↑ Number of TH-immunoreactive neurons ↑ TH cell density ↑ TH fiber outgrowth	Not assessed	[116]
Rat	6-OHDA	Striatum	Unilateral	↓ Apomorphine-induced rotation	↑ Striatal TH fiber density ↑ TH + neurons	Not assessed	[117]
Rat	6-OHDA	Striatum	Unilateral	Not performed	Not evaluated	Transfer of hα-syn from host to graft	[128]
Rhesus monkey (*Macaca mulatta*)	MPTP	Caudate nucleus	Bilateral	↓ Sedentary behavior	↑ Catecholamine fiber density	Not assessed	[118]
New world monkey (Marmoset)	MPTP	Putamen	Unilateral	↓ Amphetamine-induced rotation	↑ Striatal TH fiber density	Not assessed	[119]
Green monkey (*Cercopithecus aethiops sabeus*)	MPTP	Caudate nucleus	Unilateral Bilateral	Not performed	↑ Striatal TH nerve density ↑ TH immunoreactive cells	Not assessed	[120]
Human	Severe PD	Putamen	Unilateral	↓ Number of OFF-periods ↓ Limb rigidity & bradykinesia	↑ Putaminal DA synthesis & storage	Not assessed	[97]
Human	Unspecified	Putamen	Bilateral	UPDRS, normal daily tasks, and self-rating scale improvements Better upper extremity performance ↑ Lower extremity performance and H&Y scores	↑ TH-positive neurons Higher TH-cell density Denser TH fiber outgrowth	Not assessed	[121]
Human	Unspecified	Post-commissural putamen	Bilateral	Improvement of motor function, daily living, UPDRS scores, and lower extremity performance Resolution of dyskinesias, dystonia, and motor fluctuations	Graft survival Dense TH-positive neurons Extensive DA innervation Seamless integration without host fiber sprouting	Not assessed	[122]
Human	Unspecified	Putamen	Bilateral	Improved UPDRS motor scores Dyskinesias and motor fluctuations largely resolved	↑ Putaminal DA uptake Grafts integrated structurally with host striatum Robust DA innervation	Not assessed	[123]
Human	Severe PD	Putamen	Bilateral	Improved UPDRS and Schwab & England OFF-scores (mainly in younger patients)	↑ Putaminal DA uptake 85% graft survival	Not assessed	[124]
Human	Severe PD	Post-commissural putamen	Bilateral	Improved UPDRS motor off-period performance	↑ Putaminal DA uptake ↑ TH immunoreactivity in grafts	Not assessed	[125]
Human	Severe PD	Putamen	Unilateral	Gradual loss of l-DOPA response Progressive loss of motor function Cognitive decline starting at 14 years post-transplantation	↑ TH + neurons in grafts Complete TH reinnervation ↑ TH fiber density Absence of inflammatory response Widening of the lateral ventricles & severe medial temporal lobe atrophy	α-Syn & ubiquitin inclusions (∼12% of transplanted cells)	[46]
Human	Severe PD	Post-commissural putamen SN	Bilateral	Improvement in total & motor UPDRS scores	Restoration of putaminal DA uptake ↑ Graft reinnervation with TH fibers	Not assessed	[47]
Human	Severe PD	Caudate & putamen Putamen only	Unilateral Bilateral	↑ Health-related quality of life	↑ DA uptake in some patients	Not assessed	[48]
Human	Moderate PD	Putamen	Bilateral	No clear UPDRS-III benefit overall ↓ l-DOPA equivalent daily dose ↓ OFF-state duration ↑ Dyskinesias	Increased or stabilized putaminal DA activity	Not assessed	[126]
Human	Severe PD	Caudate & putamen Putamen only	Bilateral	Improvement of UPDRS score Independence in all activities of daily living ↓ Dyskinesias Withdrawal of DA medication	Normalization of DA uptake	Not assessed	[127]

Follow-up periods ranging from 12 to 46 months have reported improvements in quality of life, increased striatal ^18^F-DOPA uptake, and robust graft survival. A double-blind, sham-controlled trial demonstrated graft survival in 85% of patients and significant motor improvements, particularly in younger individuals^124^. In contrast, bilateral ectopic nigral transplants failed to improve MDS-UPDRS scores and were associated with postoperative dyskinesias^125^.

Postmortem analyses have confirmed long-standing graft viability. In two patients, striatal transplantation of embryonic DA cell suspensions restored ^18^F-DOPA uptake, reduced dyskinesias, and enabled extensive reinnervation^47^. One case demonstrated persistent graft survival 24 years post-implantation without signs of chronic immune activation^46^, although cognitive decline and reduced l-DOPA responsiveness emerged after 14 years, possibly due to host-to-graft *α*-SYN transmission.

Unilateral transplantation of hfVM tissue into the putamen of PD patients restored DA function (evidenced by increased ^18^F-DOPA uptake) and improved rigidity, bradykinesia, and l-DOPA-induced motor fluctuations, mainly contralateral to the graft^48^. Early dyskinesias often subsided as the graft matured. Recovery remained incomplete, reflecting variability in graft survival, reinnervation, and ongoing host DA, suggesting bilateral implantation may be needed for optimal long-term benefit.

The TransEuro trial investigated the effects of hfVM transplants in 11 patients with early-stage PD over a three-year period, using imaging (^18^F-DOPA, DAT, and serotonergic markers) and motor assessments (UPDRS III) alongside 12 months of immunosuppressive therapy. Overall, the transplants did not produce a clear improvement in motor function compared to controls, although some patients showed a positive relationship between graft survival and DA PET signals, with outcomes differing by surgical site and device used. Secondary outcomes indicated slight reductions in l-DOPA dosage and OFF time, and three patients experienced mild, non-disabling graft-induced dyskinesias. Adverse events were frequent but mostly minor, with no deaths or long-term disabilities reported^126^.

Despite encouraging findings, inconsistencies across trials have been reported, often due to differences in surgical technique, blinding, and outcome measures. A major limitation is the potential for immune rejection of allogeneic grafts, which can compromise long-term survival and functional performance^129^. Postmortem studies from additional patients revealed significant increases in striatal ^18^F-DOPA uptake and reduced motor fluctuations after transplantation, reinforcing clinical relevance^127^.

In addition to restoring DA neurotransmission, preclinical studies indicate that fVM-derived neural stem cells may exert ancillary neuroprotective effects, including the promotion of hippocampal neurogenesis and sustained post-transplant protection^130^. A major limitation, however, is the susceptibility of grafted neurons to host-to-graft *α*-SYN propagation. Postmortem analyses have shown that transplanted neurons can develop LB-like inclusions years after implantation^131^, ^132^, ^133^, ^134^, ^135^, ^136^. Experimental work in 6-OHDA-lesioned rats demonstrated that host-derived human *α*-SYN can retrogradely transfer into grafted DA neurons without direct viral transfection, forming misfolded, proteinase K-resistant inclusions thereby providing mechanistic evidence for prion-like host-to-graft *α*-SYN propagation in PD^128^. These inclusions are thought to arise through mechanisms such as exosome-mediated transfer, receptor-dependent endocytosis, or direct membrane contact, which enable extracellular *α*-SYN to misfold and spread into previously healthy grafted neurons^128^^,^^137^^,^^138^. Such propagation disrupts proteostasis, impairs mitochondrial integrity, and ultimately compromises neuronal viability.

In addition to restoring DA neurotransmission, preclinical studies indicate that fVM-derived neural stem cells may exert additional neuroprotective effects, including the promotion of hippocampal neurogenesis and sustained protection after transplantation A major limitation, however, is the vulnerability of grafted neurons to host-to-graft *α*-SYN propagation. Postmortem analyses have revealed that transplanted neurons can develop LB-like inclusions years after implantation These inclusions are thought to arise through several mechanisms, such as exosome-mediated transfer, receptor-dependent endocytosis, or direct membrane contact, that enable extracellular *α*-SYN to misfold and spread into previously healthy grafted neurons^137^, ^138^, ^139^. Such propagation can compromise proteostasis, mitochondrial integrity, and neuronal viability.

To address this challenge, multiple strategies are under investigation. These include small-molecule inhibitors of *α*-SYN aggregation^140^^,^^141^, pharmacological inducers of autophagy such as trehalose and rapamycin analogs^58^^,^^62^, and passive immunization approaches targeting extracellular *α*-SYN^59^^,^^60^. Passive immunization, in particular, offers advantages due to its high specificity, long half-life, and ability to neutralize extracellular *α*-SYN without imposing significant cellular stress or immune activation^126^. Complementary strategies involve preconditioning grafts to enhance their intrinsic resilience, for example, by overexpressing molecular chaperones chaperones^142^, lysosomal regulators^143^, or proteasome activators^144^ to bolster proteostatic and mitochondrial defenses against host-derived pathology.

Despite these advances, the widespread clinical use of fVM tissue remains limited by ethical and logistical constraints. Fetal tissue availability is restricted, and strict regulations hinder scalability. Accurate identification of A9 versus A10 DA neuron subtypes is critical for ensuring proper graft functionality. Thompson et al. used TH-green fluorescent protein (GFP) transgenic mice to distinguish these subtypes *via* GIRK2 (G-protein-regulated inward-rectifier potassium channel 2) and calbindin markers, thereby improving donor tissue characterization^145^. Generating defined yet heterogeneous DA neuron populations may support appropriate circuit integration and functional neurotransmission.

In summary, fVM-derived DA neuron grafts demonstrate long-term survival, integration, and clinical benefit. However, key obstacles remain, including immunogenicity, *α*-SYN pathology, and donor tissue limitations. Future protocols should focus on refining graft composition, enhancing resilience to host pathology, and ensuring durable integration to fully realize the therapeutic potential of fVM-based or next-generation regenerative approaches in PD.

### 3.2 Implantation of human induced pluripotent stem cell-derived dopamine neurons

hiPSCs provide a renewable, scalable, and ethically acceptable source of DA neurons for treating PD. Derived from a patient's own somatic cells, hiPSCs retain the donor's genetic identity, reducing the risk of immune rejection and potentially eliminating the need for long-term immunosuppression. They can be directed to differentiate into midbrain-specific DA neurons that closely mimic the molecular and functional properties of native SN neurons^146^, ^147^, ^148^. Their compatibility with standardized manufacturing protocols and suitability for cryopreservation further support their clinical utility and potential for personalized regenerative therapies.

Historically, fVM tissue represented the primary source for DA grafts. However, its limited availability, variability, and ethical concerns have restricted clinical application. In this context, hiPSCs provide virtually unlimited availability, greater reproducibility, and fewer ethical and logistical constraints. These features make hiPSC-derived DA neurons a promising and sustainable alternative for disease-modifying cell-based therapies in PD. Floor plate-based differentiation protocols enable efficient generation of mature, functional DA neurons capable of survival, integration, and functional restoration following transplantation^149^^,^^150^

Pioneering work established that somatic fibroblasts from patients with idiopathic PD can be reprogrammed into functional DA neurons using doxycycline-inducible lentiviral vectors^67^. Activation of Wnt and fibroblast growth factor (FGF) 8 signaling remains the gold standard for midbrain DA specification^38^, though newer approaches employing small-molecule inhibition of transforming growth factor *β* (TGF-*β*), bone morphogenetic protein, and glycogen synthase kinase 3*β* have enhanced differentiation efficiency and maturation^151^. Nevertheless, graft failure often results from challenges in maintaining DA neuron identity post-transplantation rather than intrinsic vulnerability of the grafted cells^152^.

Preclinical studies in rodent and primate models have established the safety and efficacy of hiPSC-derived DA neuron transplantation. In 6-OHDA-lesioned mice, mRNA-reprogrammed human fibroblasts differentiated into DA-like neurons that survived in the SN, promoted axonal outgrowth into target regions (*e*.*g*., dorsolateral striatum, prelimbic cortex), and alleviated motor impairments for up to 12 months, despite some apoptotic loss among grafted neurons^39^. In 6-OHDA-lesioned rats, patient-specific hiPSC-derived DA neurons survived long-term without inducing immune responses or tumor formation and reduced amphetamine- and apomorphine-induced rotational behavior^153^. Protein-based reprogramming approaches further enhanced TH ^+^ neuron survival and motor recovery^154^, while co-delivery of metabolism-regulating miRNAs improved graft quality and integration^42^.

Autologous transplantation of hiPSC-derived DA neurons has been shown to restore motor function and promote dense TH^+^ innervation in 6-OHDA-lesioned rats, with no tumorigenicity detected up to 26 weeks post-grafting^42^. Clinical-grade, non-tumorigenic DA progenitors implanted into the striatum of 6-OHDA-lesioned rats and MPTP-lesioned monkeys resulted in improved DA viability, nigrostriatal connectivity, and behavioral performance, without adverse effects^40^. iPSC-derived neurons from healthy and PD donors enhanced spontaneous activity, cell survival, and DA uptake in MPTP-treated cynomolgus monkeys^41^. Comparable outcomes were observed in MPTP-exposed macaques following unilateral transplantation of autologous iPSC-derived DA neurons, leading to restored DA uptake, elevated DOPAC levels, and improved motor function^72^^,^^155^.

In rhesus monkeys, autologous transplantation of iPSC-derived midbrain DA neurons, performed 1–3 years after MPTP infusion, alleviated motor and mood symptoms, enhanced DA activity, and did not induce persistent immune activation^43^. Major histocompatibility complex (MHC)-matched allogeneic grafts showed reduced microglial and lymphocyte infiltration, enhancing survival of iPSC-derived DA neurons in *Macaca fascicularis*^156^. In green monkeys, bilateral intracranial delivery of human parthenogenetic stem cells into the caudate, putamen, and SN led to robust DA neuron differentiation, increased striatal TH^+^ innervation, and improved behavioral outcomes over a 12-month period, with no signs of dyskinesia^157^. Key preclinical findings are summarized in Table 2^49^, ^50^, ^51^,^49^, ^50^, ^51^^,^^72^^,^^153^, ^154^, ^155^, ^156^, ^157^.

**Table 2:** hIPSc-derived DA neuron transplantation.

Species	Animal model clinical stage	Transplantation site	Injection	Behavioral phenotype	Nigrostriatal and histological outcomes	Lewy body pathology	Ref.
Mouse	6-OHDA	SN	Unilateral	↓ Amphetamine-induced rotation	↑ Graft axonal outgrowth in several brain regions ↑ Apoptotic activity ↓ Number of TH + neurons	Not assessed	[39]
Rat	6-OHDA	Dorsolateral striatum	Unilateral	↓ Rotational asymmetry No improvement in cylinder or stepping	Surviving midbrain-like DA neurons Limited axonal outgrowth Mild gliosis No inclusions/tumors	Not assessed	[153]
Rat	6-OHDA	Striatum	Unilateral	Motor recovery with high and optimal NPC doses	Stable grafts at optimal dose Survival of TH + midbrain-like neurons Poor survival of terminal cells No tumors except at high dose	Not assessed	[154]
Rat	6-OHDA	Striatum	Unilateral	↓ Rotational behavior	↑ Number of TH + neurons Widespread hNCAM^+^ innervation Absence of both teratomas & rosettes	Not assessed	[42]
Rat	6-OHDA	Striatum	Unilateral	↓ Methamphetamine-induced rotational behavior	↑ Striatal TH nerve density ↑ TH immunoreactive neurons	Not assessed	[40]
Cynomolgus monkey	MPTP	Putamen	Unilateral	Not performed	↑ TH + neuron survival Lack of intracranial hemorrhage Absence of inflammation	Not assessed	[40]
Cynomolgus monkey (*Macaca fascicularis*)	MPTP	Putamen	Bilateral	Improved neurological/motor function Recovery of spontaneous movements	↑ Putaminal DA uptake ↑ Survival of grafted DA neurons ↑ TH fiber innervation No immune response or tumor formation	Absence of LBs	[41]
Cynomolgus monkey	MPTP	Putamen	Unilateral	No significant changes in motor behavior	Surviving midbrain-like DA neurons Extensive TH fiber reinnervation Presence of mature synapses Normal mitochondrial morphology Minimal microglial activation Lack of tumor formation	Not assessed	[72]
Cynomolgus monkey	MPTP	Caudate nucleus Putamen	Unilateral	Partial recovery of PD symptoms	↑ TH-immunopositive neurons in grafts Extensive DA fiber innervation ↑ Local DA & DOPAC levels ↑ Synaptophysin immunoreactivity No microglial activation	Not assessed	[155]
Rhesus monkey (*Macaca mulatta*)	MPTP	7 tracts: 3 × Caudate 3 × Putamen 1 × SN	Unilateral	↓ Signs of mood-related disorders Autologous grafts: ↑ Clinical rating & fine motor skills ↓ Mood-related deficits Allogenic grafts: No improvement Mood deficits persist	Autologous grafts: ↑ TH^+^ cells and putaminal DA Extensive fiber outgrowth Minimal immune response Allogenic grafts: ↓ TH^+^ cells Minimal fiber outgrowth Strong immune/inflammatory response	Not assessed	[43]
Cynomolgus monkey (*Macaca fascicularis*)	MPTP	6 tracts in the putamen	Unilateral	Not performed	↑ Survival of DA neurons and reinnervation ↓ Iba-1+ and CD45+ cells Transient neuroinflammation Humoral response	Not assessed	[156]
Green monkeys (*Chlorocebus sabaeus*)	MPTP	5 tracts: 1 × Ant. caudate 1× Post. caudate 1 × Ant. putamen 1 × Post. putamen 1 × SN	Bilateral	Low-dose hpNSCs improved parkinsonism: ↓ Number of Parkscores ↑ Healthy behavior scores Lack of dyskinesia over 12 months	↑ Striatal DA/HVA content ↑ Number of SN DA neurons ↑ Reinnervation with TH fibers No evidence of tumor formation	Not assessed	[157]
Human	Unspecified	2 tracts in the putamen	Bilateral sequential	Improvement in MDS-UPDRS III & PDQ-39 scores ↓ l-DOPA requirement Absence of dyskinesias	Moderate restoration of putaminal DA uptake Graft survival No adverse histopathological findings	Not assessed	[49]
Human	Unspecified	Putamen	Bilateral	Improved MDS-UPDRS III Mild H&N improvement Stable PDQ-39 Nearly unchanged l-DOPA dose	DA progenitors engrafted and matured into TH + neurons ↑ Putaminal DA uptake Moderate DA restoration No tumor formation or inflammation	Not assessed	[50, 51]

Despite these advances, clinical implementation remains in early stages. The first completed clinical trial involved bilateral implantation of hiPSC-derived DA neurons into the putamen of a PD subject, without immunosuppression. ^18^F-DOPA PET/CT scans showed moderate presynaptic DA terminal activity near the implantation site, with sustained clinical improvements over 24 months in MDS-UPDRS III, PD questionnaire-39, and self-reported OFF periods^50^.

Ongoing trials aim to validate these early results. In 2018, Kyoto University Hospital initiated a single-arm, open-label Phase I/II study involving bilateral putaminal transplantation of hiPSC-derived DA progenitors^51^. At 24 months, the treatment yielded improved motor outcomes and increased ^18^F-DOPA uptake, supporting the safety and efficacy of this allogeneic approach^49^. A parallel open-label trial (NCT06145711), conducted by Shanghai East Hospital and XellSmart Biomedical, is currently evaluating unilateral autologous transplantation of iPSC-derived DA precursors into the globus pallidus interna in three PD patients.

In summary, hiPSC-derived DA neurons represent a promising regenerative therapy for PD. Their scalability, personalized nature, and compatibility with advanced differentiation protocols (summarized in Table 3^38^^,^^39^^,^^42^^,^^67^^,^^146^^,^^151^^,^^154^^,^^158^, ^159^, ^160^, ^161^, ^162^, ^163^, ^164^, ^165^, ^166^, ^167^, ^168^, ^169^, ^170^, ^171^), make them a viable alternative to fetal-derived sources^38^^,^^39^^,^^50^^,^^67^^,^^146^^,^^152^^,^^154^^,^^158^, ^159^, ^160^, ^161^, ^162^, ^163^, ^164^, ^165^, ^166^, ^167^, ^168^, ^169^, ^170^, ^171^. Preclinical studies support their functional efficacy, long-term survival, and integration into host circuits. However, challenges remain, particularly in optimizing neuronal identity, mitigating apoptotic cell death, and ensuring long-lasting safety. Both autologous and MHC-matched allogeneic approaches offer viable strategies for reducing immunogenicity. Ongoing clinical trials will be crucial in determining whether hiPSC-based therapies can fulfill their potential as a disease-modifying intervention in PD.

**Table 3:** Strategies for DA lineage differentiation.

Cell source	Reprogramming/differentiation strategy	Cell type	Neuronal induction/differentiation method	DA markers	Maturation period	Ref.
hESCs (HS980, HS401) hiPSCs (SM55, SM56)	Not applicable (direct conversion)	Ventral midbrain DA neurons	Direct induction: dual SMAD inhibition, ROCK inhibitor, DMH1 inhibition, CHIR99021, *β*-*mercaptoethanol*, and SB431542 48-h RA pulse SHH pathway activation (SAG) Mechanical dissociation at 9 DDC Terminal differentiation with BDNF, GDNF, and AA ROCK inhibitor used during plating and passaging	ASCL1, CLSTN2, FOXA2, LMX1A, NURR1, OTX2, PITX3, PTPRO, and TH	*In vitro:* ∼40–45 days *In vivo:* ∼7 months post-transplantation	[38]
Human dermal fibroblasts (from a 28-year-old donor)	mRNA-based reprogramming using a StemMACS kit with OCT4, SOX2, KLF4, LIN28, C-MYC, NANOG, and GFP mRNAs	Midbrain DA neurons (SNpc and VTA subtypes)	A commercial 3-step kit protocol: Floor plate specification (10 days), expansion (10 days) Neuronal maturation (≥5 days) Cells were harvested at day 26 for transplantation BDNF, GDNF, DAPT, ascorbate plated with PDL and laminin	Calbindin, DAT, EN2, FOXA2, GIRK2, LMX1A, NeuN, NURR1, OTX2, PITX3, and TH	*In vitro*: up to day 26 *In vivo*: 1‒12 months post-transplantation	[39]
Autologous human dermal fibroblasts	iPSC reprogramming	Midbrain DA progenitor cells	Differentiation to mDAPs under GMP conditions: Directed toward SNpc fate Endpoint Day 28 Quercetin treatment to eliminate undifferentiated cells Functional validation: DA secretion, electrophysiology, TH expression	hNCAM and TH	*In vitro*: 28 days *In vivo* (6-OHDA rat model): 28 days	[42]
Human dermal fibroblasts from iPD patients	DOX-inducible lentiviral transduction of OCT4, SOX2, and KLF4 (±c-MYC) Cre-lox excision for factor-free hiPSCs	DA neurons	EB-based differentiation or MS5 stromal co-culture + BMP antagonist noggin Neural induction with FGF2, FGF8, and SHH Terminal differentiation by growth factor withdrawal	TH and TUJ1	∼24 days for reprogramming + 8 days for terminal differentiation	[67]
Human dermal fibroblasts (skin biopsy from a healthy 28-year-old woman)	Reprogramming of human fibroblasts into hiPSCs using the StemMACS iPSC mRNA Reprogramming kit over 14 days	Midbrain DA neurons	Floor-plate-based protocol using the PSC DA neuron differentiation kit Dual SMAD inhibition for neural induction WNT and SHH pathway activation for midbrain patterning Maturation with BDNF, GDNF, AA, cAMP, DAPT, and TGF-*β*3	EN2, FOXA2, LMX1A, NURR1, OTX2, PITX3, and TH	*In vitro*: 22–26 days *In vivo*: 9–12 months post-transplantation	[146]
ESCs iPSCs TiPSCs	Sendai virus to establish TiPSC clones	Midbrain DA neurons	CTraS method: a cocktail of small molecules (SB431542, DM, and CHIR99021) to accelerate differentiation CdNS-MD protocol: a Modification of the CTraS method that induces a midbrain DA identity using SHH and FGF-8 Maturation phase: BDNF + GDNF + RA + DAPT	PAX6, SYNAPSIN1, and TH	*In vitro*: 30–40 days	[151]
hESCs (H1, H9) hiPSCs (2C6, SeV6)	Not applicable (direct conversion)	Midbrain-like DA neurons	Direct induction: Floor plate-based strategy Directed differentiation *via* dual SMAD inhibition + SHH (C25II, PMA), FGF8, and CHIR99021 Cultured in Neurobasal B27 medium + AA, BDNF, GDNF, TGF*β*3, and db-cAMP CHIR added from Days 3–11	Calbindin, DAT, FOXA2, GIRK2, LMX1A, NURR1, OTX2, and TH	*In vitro*: 25–80 days *In vivo*: 18–20 weeks	[152]
hiPSCs (protein-based, Retroviral, lentiviral) and hESCs (H9, HSF-6)	Protein-based reprogramming (OCT4 + SOX2 + KLF4 + MYC) fused to a cell-penetrating peptide	Midbrain-like DA neurons	Neural induction: co-culture on MS5-SHH feeder cells + ITSA (+bFGF) Midbrain patterning: co-culture on MS5-SHH + ITSA + bFGF + SHH + FGF8 Terminal differentiation: ITSA + BDNF + GDNF + cAMP	Calbindin, DAT, EN1, GIRK2, LMX1A, LMX1B, Nurr1, TH, and VMAT2	*In vitro*: 15 days *In vivo*: 8 weeks post-transplantation	[154]
Mouse and human fibroblasts (from healthy and PD donors)	Not applicable (direct conversion)	iDA neurons	Direct reprogramming via lentiviral overxpression of MASH1 (ASCL1), NURR1, and LMX1ACultured in neuronal induction medium (DMEM/F12 + supplements) + DOX for 6–24 days No intermediate progenitor stage	ALDH1A1, AADC, D2 R, DAT, TH, TUJ1, and VMAT2	12‒24 days	[158]
hiPSCs	Not applicable (direct conversion)	Midbrain DA neurons	Three induction protocols: 1. Dual inhibitor (SB431542 and DM) 2. Dual inhibitor plus SHH and FGF8 3. CHIR99021, SHH, and FGF8 combined protocol Final maturation with N2B27, cAMP, BDNF, GDNF, and AA	DAT, FOXA2, Nestin, PITX3, SOX2, and TH	*In vitro*: up to 41 days Neuronal maturation: 33‒41 days	[159]
Keratinocytes (plucked hair) and PBMCs (blood) from same donor	Reprogramming to hiPSCs using a Sendai viral vector (non-integrating)	Midbrain DA neurons	2D protocol (Kriks et al., 2011): Induction: LDN193189, SB431542, CHIR99021, SHH, C25II, PMA, and FGF8 Medium transition: KSR to N2 Maturation: BDNF, GDNF, AA, db-cAMP, TGF*β*3, and DAPT 3D protocol (Jo et al., 2016): EB formation: 4 days Induction: SB431542 or noggin, and CHIR99021 Patterning: SHH C25II and FGF8 Steps: Embedding in Matrigel and culturing on an orbital shaker Maturation: BDNF, GDNF, AA, and db-cAMP	FOXA2, LMX1A, NURR1, and TH	2D: ∼30 days 3D: ∼39 days	[160]
MEFs	Not applicable (direct conversion)	iDA neurons	Direct transdifferentiation of post-mitotic MEFs by lentiviral overexpression of the transcription factors ASCL1, NURR1, and LMX1A DOX-induced expression of transcription factorsNeuronal-inducing medium (DMEM/F12 + insulin, transferrin, selenium, progesterone, and putrescine) No pluripotency stage involved	EN1, FOXOA1, FOXOA2, TUJ1, TH, and VMAT2	*In vitro*: 14 days post-induction	[161]
hESCs (H9, H7) hiPSCs (C4, N3)	Reprogramming using episomal vectors carrying with (OCT4, SOX2, KLF4, and L-MYC) and 2 microRNA clusters (miR-302s and 200c)	Midbrain DA progenitors and neurons	Directed differentiation using dual-SMAD inhibition + SHH and WNT activation Physical confinement *via* “spotting” method Maturation factors include BDNF, GDNF, and AA	LMX1A, FOXA2, and NURR1	∼28 days	[162]
Human dermal fibroblasts from PD patients with mutations in PARK2 or PARK8 genes	Lentiviral and Sendai virus-based reprogramming to iPSCs	DA neurons	Three-step 2D protocol: Step 1 (Days 0–14): dual SMAD inhibition using noggin, SB431542, DM to generate early neural progenitors Step 2 (Days 14–24): Ventral midbrain patterning using SHH, FGF8, and PMA to produce midbrain neuronal progenitors Step 3 (Days 24–38+): Maturation using Neurobasal medium with B27 supplement, BDNF, GDNF, AA, and forskolin to obtain postmitotic DA neurons	CALB1, DAT1, SOX6, Synaptophysin, and TH	Minimum: 38 days Full maturation: up to 65 days	[163]
hESCs (H9) hiPSCs (C001, C002, C005)	Not applicable (direct conversion)	A9 DA neurons	Direct induction: dual-SMAD inhibition (SB431542, DM) SHH and WNT activation (PM, CHIR) Maturation with BDNF, GDNF, TGF*β*3, db-cAMP, and DAPT Optimized timing and concentration for iPSCs	ALDH1A1, Calbindin, Corin, EN1, FOXA2, LMX1A, LMX1B, NURR1, and TH	60‒70 days	[164]
Gingival and dermal fibroblasts (healthy and PD patients)	Sendai virus reprogramming to obtain hiPSCs for pharmacological Induction	DA neurons	Pharmacological protocol: Induction phase: dual SMAD inhibition (LDN193189 + SB431542) + SHH + PMA + CHIR99021 Maturation phase: BDNF + GDNF + TGF*β*3 + cAMP + AA Direct conversion with AAV viral vector protocol: AAV-mediated expression of transcription factors (LMX1A, NURR1, and PITX3) Differentiation medium: BDNF, GDNF, TGF*β*3, db-cAMP, AA, and DAPT	AADC, DAT, LMX1a, NURR1, PIXT3, and TH	∼20‒25 days	[165]
hESCs (H9, H1) hiPSCs (DANi002C)	Not applicable (direct conversion)	Mesencephalic DA neurons	Days 0–9: dual-SMAD inhibition (SB431542, LDN193189), CHIR99021, SHH, SAG for floor plate induction Days 9–11: FGF8 for midbrain patterning Days 11–16: FGF8, AA, LM22A4, BDNF, db-cAMP, GDNF, and DAPT Days 16–30: same supplements, plated on poly-l-ornithine and laminin Y-27632 on days 0, 11, 16, and 24	CALB1, EN1, FOXA2, GIRK2, LMX1A, OTX2, and TH	*In vitro*: up to 62 days *In vivo*: behavioral and histological assessments at 8, 18, and 26 weeks post-transplantation	[166]
hiPSCs (201B7)	Not applicable (direct conversion)	Midbrain DA neurons	Neurosphere-based protocol: CTraS induction (Days 0–5): SB431542, DM, CHIR99021 Neurosphere formation (days 5–8): SB431542, Y27632, and bFGF Ventral midbrain patterning (day 8+): CHIR99021 and PMA Terminal differentiation (day 19+): BDNF, GDNF, AA, db-cAMP, TGF-*β*3, DAPT, and CHIR99021 (Day 19 only)	ALCAM, Calbindin, Corin, FOXA2, GIRK2, LMX1A, LRTM1, NURR1, PITX3, and TH	*In vitro*: ∼36 days (19 days to neurosphere + 17 days post-plating) *In vivo*: 16 weeks post-transplantation	[167]
Mouse and human PSCs (ESCs, iPSCs)	Not applicable (direct conversion)	iDA neurons	One-step transcription factor induction with six midbrain-specific factors (ASCL1, NURR1, LMX1A, EN1, FOXA2, and PITX3) *via* piggyBac system Maturation supported by BDNF and GDNF Validated by marker expression, electrophysiology, and DA production	AADC, DAT, EN1, FOXA2, GIRK2, LMX1A, PITX3, TH, and VMAT2	∼3 weeks	[168]
hPSCs	Not applicable (direct conversion)	VM midbrain DA progenitors	Direct induction: dual SMAD inhibition (SB431542 + noggin), CHIR99021, SHH-C24II, and FGF8b Maturation phase: BDNF + GDNF + AA + db-cAMP + DAPT	EN1, FOXA2, GIRK2, LMX1A, OTX2, and TH	*In vitro*: 16–40 days *In vivo*: 16–24 weeks	[169]
hEFs HFL1 hFFs	Not applicable (direct conversion)	iDA neurons	Direct conversion *via* lentiviral overexpression of ASCL1, BRN2, MYT1L + LMX1A, and FOXA2 Converted cells were grown in N3 neural induction medium with DOX for at least 7 days	AADC, NURR1, and TH	28‒32 days	[170]
Human fetal lung fibroblasts (IMR-90)	Not applicable (direct conversion)	DA neuron-like cells	Direct chemical induction: VPA, RepSox, kenpaullone, forskolin, Y-27632, PMA, SHH, FGF-8b, bFGF, WNT1, and WNT5a Maturation: Forskolin, kenpaullone, AA, SHH, FGF-8b, bFGF, BDNF, and GDNF	DAT, DDC, NURR1, and TH	6–8 days induction + 7–14 days maturation	[171]

Table 3. Glossary of abbreviations. AADC (Aromatic l-amino acid decarboxylase), AA (Ascorbic acid), AAV (Adeno-associated virus), ALCAM (Activated leukocyte cell adhesion molecule), ALDH1A1 (Aldehyde dehydrogenase 1 family member A1), Ascl1/MASH1 (Achaete-scute homolog 1), BDNF (Brain-derived neurotrophic factor), BMP (Bone morphogenetic protein), Brn2/POU3F2 (POU domain class 3 transcription factor 2), bFGF/FGF2 (Basic fibroblast growth factor), FGF8/8b (Fibroblast growth factor 8 isoforms), Foxa1/2 (Forkhead box proteins A1/A2), GDNF (Glial cell-derived neurotrophic factor), GFP (Green fluorescent protein), GIRK2 (G-protein-activated inwardly rectifying potassium channel 2), hESCs/hiPSCs/hPSCs (Human embryonic/induced pluripotent/pluripotent stem cells), hNCAM (Human neural cell adhesion molecule), ITSA (Insulin-Transferrin-Selenium-Sodium Pyruvate), KLF4 (Kruppel-like factor 4), Lmx1a/b (LIM homeobox transcription factors 1*α*/1*β*), LRTM1 (Leucine-rich repeats and transmembrane domain protein 1), mDAPs (Midbrain dopaminergic progenitors), MEFs (Mouse embryonic fibroblasts), MYT1L (Myelin transcription factor 1-like), NeuN (Neuronal nuclei), Nurr1 (Nuclear receptor related 1 protein), OCT4 (Octamer-binding transcription factor 4), Otx2 (Orthodenticle homolog 2), PAX6 (Paired box protein 6), PBMCs (Peripheral blood mononuclear cells), Pitx3 (Paired-like homeodomain transcription factor 3), RA (Retinoic acid), RepSox/SB431542/LDN-193189/DMH1 (small-molecule pathway inhibitors), SHH/SHH-C24II (Sonic Hedgehog, isoform C24II), SNpc (Substantia nigra pars compacta), Sox2 (SRY-box transcription factor 2), TGF-*β*3 (Transforming growth factor *β*3), TH (Tyrosine hydroxylase), TiPSCs (T cell-derived induced pluripotent stem cells), TUJ1 (Neuron-specific class III *β*-tubulin), VMAT2 (Vesicular monoamine transporter 2), VPA (Valproic acid), VTA (Ventral tegmental area), Wnt1/5a (Wnt family members 1 and 5a), Y-27632 (ROCK inhibitor).

### 3.3 Mesenchymal stromal cell transplants

MSCs are being investigated as supportive therapy for PD due to their immunomodulatory, anti-inflammatory, and neurotrophic properties^172^. While they do not inherently produce DA, MSCs secrete growth factors and cytokines that promote endogenous DA neuron survival, suppress neuroinflammation, and facilitate tissue repair. Their ease of isolation from adult tissues, low immunogenicity, and absence of tumorigenic potential make them a safe and ethically non-controversial therapeutic option^173^.

MSCs are multipotent cells capable of differentiating into derivatives of the mesoderm, ectoderm, and endoderm. Unlike embryonic stem cells (ESCs) or iPSCs, MSCs are not associated with ethical concerns or the risk of teratoma formation, enhancing their suitability for clinical applications. As non-hematopoietic progenitors, MSCs can be harvested from a variety of sources, including bone marrow, adipose tissue, skin, peripheral blood, and placenta^174^. Several studies show MSCs can acquire neuronal-like phenotypes, expressing markers such as neuron-specific enolase, nestin, and glial fibrillary acidic protein^175^^,^^176^. Pharmacological stimulation with 3-isobutyl-1-methylxanthine and forskolin activates cyclic adenosine monophosphate (cAMP) signaling, enhancing neural differentiation and neuronal characteristics^177^.

Preclinical evidence supports the neurorestorative potential of MSCs in PD. Intrastriatal transplantation of MSCs in MPTP-injected mice resulted in partial functional recovery and survival of TH ^+^ cells^178^. In 6-OHDA-lesioned rats, transplantation of rat bone marrow-derived MSCs (BM-MSCs) into the striatum or SN reduced rotational asymmetry, upregulated DA-associated markers (DA receptor 2, VMAT2, and DAT), and increased TH immunolabeling compared to sham controls^176^^,^^179^. These benefits are attributed to MSC-mediated neuroprotection *via* immunomodulation and trophic support^180^. Findings are summarized in Table 4^55^^,^^178^, ^179^, ^180^, ^181^, ^182^.

**Table 4:** Mesenchymal stromal cell transplantation.

Species	Animal model clinical stage	Transplantation site	Injection	Behavioral phenotype	Nigrostriatal and histological outcomes	Lewy body pathology	Ref.
Mouse	MPTP	Striatum	Unilateral	Significant motor recovery	Survival of MSCs in the striatum Partial fiber restoration near grafts	Not assessed	[178]
Rat	6-OHDA	Striatum	Unilateral	↓ Attenuation of amphetamine-induced rotational behavior	Preservation of TH + neurons ↑ Striatal DA release Partial restoration of DA markers (TH, DAT, VMAT2) in the striatum and SN	Not assessed	[179]
Rat	6-OHDA	SN	Unilateral	Not performed	MSC survival in the SN Stimulated subventricular zone neurogenesis No rescue of nigral TH + neurons Lack of DA differentiation	Not assessed	[180]
Rat	6-OHDA	Striatum	Unilateral	↓ Amphetamine-induced behavior (in DI-MIAMI + PAMs-NT3) DI-MIAMI alone or PAMs-blank showed minimal or moderate improvement	Partial increase in ipsilateral SN TH + neurons ↑ Striatal TH fibers TH expressed in grafted cells only with PAMs ↑ Expression of DA markers (DAT, TH, Nurr1) in DI-MIAMI + PAMs-NT3 Cells alone or PAMs-Blank showed minimal or no effect	Not assessed	[181]
Rat	6-OHDA	Striatum	Unilateral	Partial motor recovery: ↓ Rotational behavior ↑ Spontaneous behavior Modest forelimb improvement	SN TH number and striatal fiber density ↑ with low MSC co-grafting but ↓ with high MSCs Graft volume unchanged Reinnervation area and DA survival depended on MSC dose	Not assessed	[182]
Human	Unspecified	Subventricular zone	Unilateral	UPDRS scores improved in both “OFF” and “ON” periods No change in H&Y scores Slight reduction in l-DOPA dose Marginal activities of daily living and dyskinesia improvement	Moderate putaminal DA restoration BM-MSCs survived, integrated, and migrated to ipsilateral SN Lack of parenchymal modifications without tumor development	Not assessed	[55]

However, low neuronal marker expression and poor graft survival remain major limitations. To overcome this, biodegradable, pharmacologically active microcarriers were combined with marrow-isolated adult multilineage inducible cells (a subpopulation of hMSCs) and implanted stereotaxically into the striatum of 6-OHDA-lesioned rats. This approach led to improved graft survival, behavioral recovery, and enhanced functionality of the nigrostriatal system^181^. Co-grafting low doses of MSCs with midbrain DA neurons has also improved outcomes in 6-hydroxydopamine (6-OHDA) hemiparkinsonian rats by reducing rotational behavior, enhancing spontaneous locomotion, and promoting graft survival and axonal reinnervation^182^.

Clinically, an open-label pilot trial tested unilateral autologous MSC transplantation into the subventricular zone of PD patients, showing UPDRS improvements in OFF and ON states after 32 months, with no tumor formation or structural brain changes, and reduced dyskinesia in some cases^55^. Ongoing studies include a Phase I pilot trial (NCT02611167) and two Phase II randomized, double-blind trials (NCT04928287, NCT04506073) evaluating BM-MSCs as potential disease-modifying therapy.

A novel, unexplored avenue involves combining MSCs with biohybrid drugs exhibiting dual biological activity, potentially boosting homing efficiency and immunoregulatory functions^54^. Overall, MSCs show promise as a safe, multipotent, and neuroprotective cell-based approach for PD. Preclinical models demonstrate their ability to induce neuronal-like differentiation, increase DA marker expression, and improve motor function, while early clinical data suggest safety and potential benefit. Key challenges, including limited neuronal differentiation, low graft survival, and variable efficacy, may be addressed through biomaterial scaffolds, co-transplantation, and pharmacological enhancement. Results from ongoing randomized trials will determine their long-term feasibility and clinical utility.

### 3.4 Intracranial delivery of carotid body glomus cells

CB glomus cells represent a neuroprotective approach for PD owing to their dual capacity to produce DA and secrete high levels of glial cell line-derived neurotrophic factor (GDNF)^183^^,^^184^. Although lacking DAT expression, these cells display strong resistance to neurotoxic insults and exert trophic effects that support neuronal survival, reinnervation, and functional recovery through both neurochemical and non-canonical mechanisms^183^.

Preclinical studies in rodent and non-human primate models demonstrate robust antiparkinsonian effects of CB grafts. In both mice and monkeys, CB cell transplantation conferred resistance to MPTP-induced DA neurodegeneration despite lacking DAT expression^183^^,^^185^. In MPTP-lesioned mice, striatal CB implants improved rearing behavior, preserved nigrostriatal pathway integrity, and significantly upregulated GDNF mRNA expression without triggering detectable inflammatory responses^186^.

Similarly, in 6-OHDA-lesioned rats, striatal CB grafts ameliorated motor deficits in a subset of animals, prevented ongoing DA neuron loss, promoted DA terminal sprouting, and enhanced neurotrophic signaling^183^. Transplantation of CB cell aggregates further alleviated motor and sensory impairments, enhanced calcium-dependent DA release, and induced host striatal reinnervation^187^. Table 5^52^^,^^53^^,^^183^, ^184^, ^185^, ^186^, ^187^, ^188^ offers a consolidated summary of the most relevant preclinical and clinical findings outlined in the preceding section.

**Table 5:** Carotid body glomus cell transplantation.

Species	Animal model clinical stage	Transplantation site	Injection	Behavioral phenotype	Nigrostriatal and histological outcomes	Lewy body pathology	Ref.
Mouse	MPTP	Striatum	Unilateral	Not performed	CB grafts survived and integrated Preservation of the nigrostriatal system ↑ Striatal GDNF expression in glomus cells No tumor formation Mild gliosis around the graft	Not assessed	[184]
Mouse	MPTP	Striatum	Unilateral	↑ Number of rearing events	↑ Striatal TH fiber innervation ↑ SN TH + neurons Positive correlation between graft size and SN DA neuron protection No changes in striatal or SN GFAP immunoreactivity CB grafts survived and integrated, with no tumor formation	Not assessed	[186]
Rat	6-OHDA	Striatum	Unilateral	Group I: normalized amphetamine/apomorphine responses Group II: poor motor recovery with persistent DA supersensitivity	Production of neurotrophic factors CB grafts survived long-term Striatal reinnervation by ipsilateral SN and VTA neurons ↑ GDNF levels DAT-negative	Not assessed	[183]
Rat	6-OHDA	Striatum	Unilateral	Improved motor and sensorimotor function (amphetamine-induced rotations & whisker-mediated touch) ↓ Anxiety-like behavior (open field thigmotaxis)	CB grafts survived long-term ↑ Striatal TH-positive neurite outgrowth Partial restoration of striatal DA	Not assessed	[187]
Cynomolgus monkey (*Macaca fascicularis*)	MPTP	Putamen	Unilateral	↓ Tremor and bradykinesia Recovery of balance and spontaneous activity ↓ Disability score (40%–50%) ↑ Improved fine motor skills Persistent apomorphine-induced rotations	Grafted glomus cells survived Extended TH + fibers Induced robust ipsilateral striatal and caudate reinnervation	Not assessed	[185]
Cynomolgus monkey (*Macaca fascicularis*)	MPTP	2 tracts into the post-commissural putamen	Unilateral Bilateral	Long-term improvement in global motor assessment and fine motor tasks (maximal at 6 months and stable up to 12 months)	↑ Striatal^18^F-DOPA uptake at 12 months Total SN TH-positive neurons unchanged Bilateral ↑ SN TH-positive neurons expressing GDNF	Not assessed	[188]
Human	Advanced	Patients 1–4: 3 putaminal targets (anterior, middle, posterior) Patients 5–6: added caudate nucleus target	Bilateral	UPDRS “OFF” scores improved in most patients, peaking at 6 months No OFF-period dyskinesias	CB trophic support Putaminal DA uptake not directly changed Efficacy declined with patient age	Not assessed	[52]
Human	Advanced	Patients 1–4: 3 putaminal targets (anterior, middle, posterior) Patients 5–6: added caudate nucleus target	Bilateral	Enhanced UPDRS III OFF-medication score (including bradykinesia and rigidity)Improved Schwab and England, UPDRS II/IV scale scores No OFF-period dyskinesias	Higher CB integrity Presence of TH + cells in transplanted tissue Trend toward stabilized or slightly increased putaminal DA uptake	Not assessed	[53]

CB aggregate grafts into the putamen of cynomolgus monkeys improved motor performance, increased DA uptake in the grafted striatum, and increased the number of TH ^+^ neurons expressing GDNF^188^. In MPTP-treated *Macaca fascicularis*, unilateral CB cell transplantation targeted to the putamen significantly ameliorated tremor, bradykinesia, akinesia, and postural instability, accompanied by enhanced TH ^+^ reinnervation and greater survival of glomus cells^185^.

Clinical evidence from a phase I/II blinded trial indicated that CB autotransplantation improved total UPDRS scores in several patients, with minimal OFF-period dyskinesias observed between 6 and 12 months post-surgery^52^^,^^53^. Follow-up at 3 years indicated that 50% of patients maintained improved motor function compared to baseline. Sustained DA uptake in the putamen was detected, with benefits achieved using fewer cells than typically required for fetal tissue transplantation, and without inducing OFF-period dyskinesia. However, donor CB glomus cell scarcity remains a major limitation.

Collectively, CB glomus cell transplantation represents a promising neuroprotective strategy for PD. By combining DA activity with potent trophic support, these grafts preserve the nigrostriatal system, promote reinnervation, and improve motor function, even in the absence of DAT-mediated DA recycling. Evidence from preclinical and clinical studies underscores their therapeutic potential, though interindividual variability and significant limited cell availability remain important obstacles. Advancing scalable CB glomus cell expansion, refining surgical delivery protocols, and conducting long-term efficacy studies will be essential to confirm durability of benefit. Addressing these challenges could position CB transplantation as a viable and clinically translatable neurorestorative therapy for PD.

### 3.5 Comparative insights into cell sources for Parkinson's disease therapy

Neuroregenerative therapies for PD face persistent challenges, particularly in achieving durable graft survival and functional integration. Several cell sources have been explored, including fVM tissue, hiPSC-derived DA neurons, MSCs, and CB glomus cells, each exhibiting distinct strengths and limitations. Comparative evaluation highlights trade-offs between intrinsic functional maturity, scalability, immune compatibility, and ethical feasibility, building on the detailed characterization in Sections 1, 2, 3.

fVM grafts remain the benchmark for functional efficacy, demonstrating robust DA neuron characteristics. Clinical studies document transplanted A9-type DA neurons surviving for over two decades and contributing to sustained motor improvements^114^^,^^115^^,^^119^^,^^126^^,^^127^. Broader application is constrained by donor scarcity, variability in tissue quality, ethical considerations, and immunogenicity mediated by MHC class I/II expression^124^.

hiPSC-derived DA neurons provide a renewable, scalable, and ethically acceptable alternative, with potential for autologous transplantation. Preclinical and early clinical studies show midbrain DA phenotypes and axonal extension^40^^,^^42^^,^^43^^,^^49^^,^^72^^,^^155^. Residual immunogenicity may arise from aberrant antigen expression or differentiation-induced MHC upregulation^56^^,^^57^.

MSCs, derived from bone marrow, adipose tissue, placenta, or other sources, primarily act *via* paracrine mechanisms, secreting neurotrophic and immunomodulatory factors such as GDNF, brain-derived neurotrophic factor (BDNF), TGF-*β*, and IL-10^189^^,^^190^. Their immunoprivileged status and favorable safety profile make them suitable as adjunctive therapies alongside neuronal grafts^55^.

CB glomus cells secrete DA and are enriched in GDNF, providing trophic support and resistance to neurotoxins^183^, ^184^, ^185^, ^186^, ^187^, ^188^, though limited DA reuptake due to absent DAT constrains functional efficacy. Clinical studies are limited^52^^,^^53^, but their protective properties suggest utility in combinatorial strategies.

**Figure 5:**
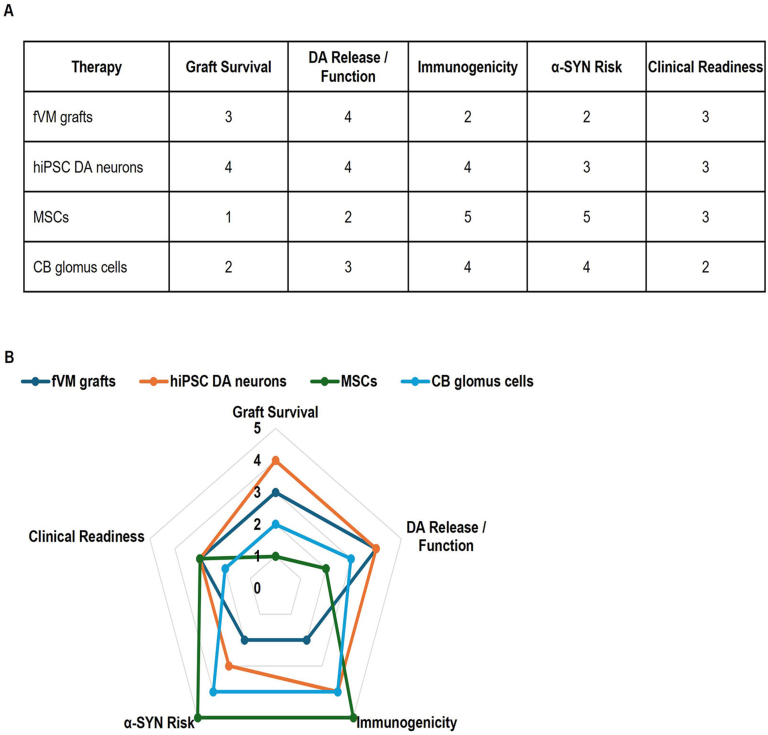
Quantitative assessment of cell-based therapies for Parkinson's disease using outcome scores. (A) Summary table of efficacy measures from preclinical and clinical studies of fetal ventral midbrain (fVM) grafts, human induced pluripotent stem cell (hiPSC)-derived dopamine (DA) neurons, mesenchymal stromal cells (MSCs), and carotid body (CB) glomus cells. Measures are scored according to criteria established in this study. (B) Radar plot integrating five key outcome domains: graft survival, DA release and function, immunogenicity, *α*-synuclein (*α*-SYN) propagation risk, and clinical readiness. Scores are normalized on a 0–5 scale, with larger polygon areas indicating greater overall therapeutic effect based on the scoring system.

Overall, fVM grafts provide the strongest evidence of long-term survival and functional restoration, hiPSC-derived DA neurons offer versatility and scalability, and MSCs and CB glomus cells provide neurotrophic and immunomodulatory support that can complement primary neuronal grafts. These functional and translational differences are summarized in Fig. 5 and Table 6, providing a comparative overview of cell sources for PD therapy.

**Table 6:** Comparison of cell sources for PD: mechanisms, characteristics, and clinical considerations.

Therapy/cell type	Cell source	Therapeutic mechanism	Neuronal features	Graft survival	Immune compatibility	Tumorigenicity	Clinical status	Advantages	Limitations	Key challenges
fVM grafts	Fetal ventral mesencephalon	DA replacement, host integration Moderate neurotrophic support	A9-type DA neurons Synaptic integration	>20 years (human data)	Allogeneic, needs immunosuppression	None (post-mitotic)	Early trials (TRANSEURO)	Proven restoration Long-term survival	Ethical/logistical issues Immune rejection α-Syn pathology	Standardization α-Syn mitigation Donor availability
hiPSC-derived DA neurons	Patient-derived iPSCs	DA replacement, personalized therapy Minimal neurotrophic support	Midbrain DA phenotype Synaptic integration	2–3 years (preclinical and early clinical)	Autologous or HLA-matched	Low (validated protocols)	Phase I/II trials ongoing	Scalable ↓ Immunogenicity Unlimited supply	DA identity maintenance Apoptotic loss	Identity integration Long-term safety
MSCs	Bone marrow Skin Blood Adipose tissue Placenta	Immunomodulation High neurotrophic support (GDNF, BDNF)	Absent but with paracrine support	Poor without scaffolds	Immunoprivileged	None	Phase I/II trials ongoing	Safe ↓ Immunogenicity Ethical	Limited DA production Poor survival	Differentiation Survival Functional efficacy
CB glomus cells	Carotid body	DA + GDNF secretion Very high neurotrophic support (GDNF-enriched)	DA-releasing (TH^+^/DAT) Paracrine support	Moderate (>3 years)	Autologous transplantation	None	Early preclinical and small clinical studies	Neurotoxin resistance Trophic support	Limited DA release Lack of DAT Limited data	Scaling Integration Long-term outcomes

### 3.6 Translational patterns, immune compatibility strategies, and decision-guiding trends among candidate cell therapies

Building on the comparative framework of Section 3.5, translational patterns emerge across PD cell therapies. Methodological heterogeneity, including species differences, cell preparation protocols, transplantation methods, and outcome measures, precludes formal meta-analysis, yet convergent findings on graft longevity, functional impact, and clinical feasibility provide actionable guidance for future translation and highlight key translational barriers that need innovative solutions.

fVM grafts offer the clearest evidence of durable benefit, although ethical constraints, limited donor tissue, and the requirement for transient immunosuppression restrict broader application^46^^,^^47^^,^^124^. Immune rejection in fVM grafts primarily arises from MHC class I/II expression on donor cells, which can trigger host T cell-mediated responses, while microglial activation contributes to graft inflammation and long-term attrition. Standard systemic immunosuppressive regimens remain necessary, but innovative strategies are emerging to minimize peripheral exposure and directly address immune rejection. Localized delivery of slow-release immunosuppressants embedded within biomaterials at the graft site maintains graft tolerance while reducing systemic immunosuppression, exemplifying a synergistic approach that integrates cell therapy with engineered scaffolds to enhance graft survival.

hiPSC-derived DA neurons provide translational flexibility. Autologous hiPSC grafts are largely tolerated due to self-MHC expression, though aberrant antigen expression or differentiation-induced MHC upregulation can provoke immune responses^39^^,^^42^^,^^153^^,^^154^. Allogeneic hiPSC grafts, in contrast, risk conventional T cell-mediated rejection unless HLA-matched or engineered for immune evasion. Cutting-edge hypoimmunogenic hiPSC lines, created through targeted disruption of MHC class I/II genes and overexpression of immune checkpoint molecules such as CD47, reduce recognition by host T and NK cells, enabling “off-the-shelf” transplantation^56^^,^^57^. HLA-matched hiPSC banks further improve compatibility while facilitating scalable clinical application. Combining these engineered cell lines with localized biomaterials represents a synergistic strategy that simultaneously tackles immune rejection and graft viability, providing a clear translational roadmap for future clinical trials.

Beyond primary neuronal grafts, cell types with paracrine activity, such as MSCs and CB glomus cells, act as supportive adjuncts rather than standalone DA replacements. Through secretion of neurotrophic and anti-inflammatory factors, these cells modulate local immune responses, enhance host microenvironments, and improve the integration and survival of co-transplanted DA neurons^174^^,^^183^^,^^184^^,^^191^. Strategic combinations of engineered neurons, supportive cell types, and biomaterial scaffolds thus constitute a multifaceted, synergistic strategy to overcome translational challenges in PD therapy, explicitly addressing both immune and functional limitations.

Overall, decision-making in clinical translation must explicitly balance efficacy, scalability, and immune compatibility. Selecting autologous, HLA-matched, or hypoimmunogenic engineered platforms based on patient needs and feasibility, coupled with localized immunomodulation and biomaterial-based delivery systems, provides a forward-looking framework to optimize graft viability and guide future clinical adoption in PD therapy.

### 3.7 Electrophysiological evidence of graft-host integration

Histological and behavioral assessments have long served as the primary endpoints for evaluating cell transplantation in PD. However, electrophysiological analyses provide a critical benchmark for therapeutic success, offering direct proof that transplanted neurons are not only surviving but also forming functional synapses and participating in host circuitry. These studies establish functional integration as the *sine qua* non of restorative grafting.

*In vitro* patch-clamp recordings confirm that hiPSC-derived DA neurons fire both spontaneous and evoked action potentials, receive excitatory and inhibitory postsynaptic currents, and respond to afferent stimulation, hallmarks of mature neural connectivity^192^^,^^193^. Host afferents form functional synapses onto grafted neurons, while transplanted cells progressively acquire the electrophysiological properties required for participation in basal ganglia circuits.

*In vivo* studies further support these findings. In rodent PD models, transplanted DA neurons receive excitatory input from striatal and cortical projections while extending axons into striatal targets. Long-term recordings in 6-OHDA-lesioned immunodeficient mice revealed that hiPSC-derived DA neurons maintain stable firing patterns for more than one year, displaying tonic and burst firing characteristic of native nigral neurons^146^.

Organoid-based models provide additional insight into network-level physiology. Human midbrain organoids derived from hiPSCs progressively express DA markers, exhibit activity-dependent DA release, and demonstrate synchronized bursting on multi-electrode arrays, indicative of coordinated neuronal activity^194^. Following transplantation, patch-clamp recordings from EGFP^+^ organoid-derived neurons revealed spontaneous and evoked action potentials, afterhyperpolarization “sags,” delayed inward rectification, and functional voltage-gated Na^+^ and K^+^ currents, confirming their maturation into electrophysiologically competent networks *in vivo*.

Bidirectional communication between grafts and host circuits has also been documented. In organotypic and *in vivo* preparations, grafted mouse ventral mesencephalic neurosphere-derived DA neurons engaged in polysynaptic interactions with host neurons. Silencing striatal neurons increased excitatory drive to transplanted cells, while optogenetic stimulation of channel rhodopsin 2-transduced grafted neurons enhanced excitatory currents in neighboring graft neurons, revealing robust intragraft and host-graft connectivity^195^.

Furthermore, optogenetics has been employed to directly manipulate grafted neurons, demonstrating their essential role in mediating therapeutic effects. hESC-derived DA neurons engineered to express halorhodopsin could be selectively silenced *in vitro*, suppressing both firing and DA release^196^. After transplantation into parkinsonian mice, these neurons restored motor function and integrated into striatal circuitry. Strikingly, optogenetic silencing of the grafts abolished behavioral recovery, directly indicating that graft activity is indispensable for therapeutic benefit. These experiments highlight that DA-rich grafts not only survive but also modulate host glutamatergic transmission and drive circuit repair.

Collectively, electrophysiological evidence demonstrates that successful transplantation requires more than survival and DA release; it depends on authentic synaptic integration into basal ganglia circuits. Future translation will benefit from *in vivo* patch-clamp methods, multimodal imaging, and closed-loop neuromodulation to rigorously characterize graft-host communication. Establishing standardized electrophysiological endpoints will be essential to define the quality, stability, and clinical relevance of neuronal integration in PD cell therapies.

These studies confirm that grafted neurons can integrate into host networks, prompting the next question: how might this integration influence non-motor symptoms and higher-order brain functions?

### 3.8 Impact of cell therapies on non-motor symptoms and higher-order circuitry

While cell transplantation consistently improves motor symptoms in PD, many patients and animal models continue to experience non-motor deficits, such as cognitive impairment, mood disturbances, sleep disorders, gastrointestinal dysfunction, and autonomic dysregulation, which often emerge early and significantly impair quality of life^197^. These deficits largely reflect degeneration in extranigral regions, including limbic structures, prefrontal cortex, brainstem nuclei, peripheral autonomic circuits, and the hippocampus, alongside widespread *α*-SYN accumulation that disrupts neurogenesis and contributes to depression^198^.

Most cell-based research emphasizes motor recovery, with limited systematic evaluation of cognitive and affective endpoints. Nonetheless, emerging evidence suggests that transplanted DA neurons can integrate into host networks and modulate cortical and subcortical activity, potentially improving emotional, cognitive, and autonomic functions. Preclinical studies confirm that DA modulation extends beyond motor control: mesolimbic DA supports emotional processing, projections to the prelimbic cortex and dorsomedial striatum regulate cognitive flexibility and goal-directed behavior, and dorsolateral striatal circuits contribute to anxiety regulation and decision-making^199^, ^200^, ^201^.

Rodent models with partial or bilateral 6-OHDA lesions recapitulate working memory^202^^,^^203^ and emotional processing^204^ deficits, observed in patients. Some impairments respond to acute (but not chronic) l-DOPA administration, highlighting a narrow therapeutic window^205^. Pharmacological DA restoration improves working memory, response inhibition, and reinforcement learning, correlating with increased prefrontal cortex and caudate activity^206^, ^207^, ^208^. Clinical trials support the role of DA in affective regulation. For example, pramipexole reduced depressive symptoms independently of motor effects in a 12-week randomized trial^209^. Conversely, apathy frequently emerges after subthalamic nucleus DBS, often linked to DA agonist withdrawal and decreased mesolimbic DA, with preoperative non-motor fluctuations and anxiety predicting susceptibility.

fVM grafts have alleviated cognitive and neuropsychiatric deficits in preclinical models. In 6-OHDA-lesioned rats, striatal reinnervation reduced apathetic-like behavior and restored goal-directed actions^210^^,^^211^. DA receptor 2-mediated reductions in cerebral blood volume further implicate DA in pain modulation. Clinical studies corroborate these findings, showing that patients receiving fVM transplants performed similarly to healthy controls on cognitive tests. Improvements in immediate and delayed verbal recall were observed at 12–24 months, although these benefits declined by 36 months^212^. A double-blind trial of 40 patients showed cognitive stability following bilateral fVM transplantation, confirming safety despite limited initial benefit^213^.

Early-phase trials of allogeneic iPSC-derived DA progenitors indicate safety, with some subjective non-motor improvements reported (*e*.*g*., MDS-UPDRS III, PD questionnaire-39), although effects were inconsistent at 24 months^49^. MSCs may improve non-motor outcomes by differentiating into serotonergic, cholinergic, and noradrenergic phenotypes. Autologous MSC delivery *via* intravenous or intranasal routes produced short-term gains in depression, sleep quality, daytime sleepiness, and overall non-motor symptom scores^214^.

A pilot study of unilateral autologous BM-MSC transplantation observed motor improvements accompanied by reduced freezing, enhanced facial expression, and lower medication requirements, suggesting mood and cognitive-motor integration^55^. CB glomus cells may influence non-motor symptoms *via* paracrine modulation of noradrenergic and serotonergic systems essential for mood and autonomic balance^215^.

Mechanisms underlying these effects remain incompletely defined. Beyond DA replacement, transplanted cells may act *via* retrograde signaling, glial modulation, and stimulation of endogenous plasticity through local microenvironment enhancement. Grafts may indirectly influence higher-order networks by reshaping host activity or supporting repair in extranigral regions. Emerging tools, including multimodal imaging and single-cell transcriptomics, will be valuable for clarifying how transplanted cells engage limbic and cortical circuits to modulate behavioral outcomes.

In summary, non-motor symptoms constitute a critical but under-addressed component of PD. Evidence indicates that transplanted neurons can influence cognitive, affective, and autonomic domains, particularly when projections reach associative or limbic regions. Successful therapy depends not only on cell survival and DA release but also on the precise reconstruction of functional host-graft circuitry. Future clinical studies should therefore incorporate non-motor endpoints and explore cell types or combinatorial strategies capable of addressing the full pathological spectrum of PD.

Importantly, the potential of cell therapies may be maximized when combined with existing pharmacological and surgical interventions (*e*.*g*., levodopa, DBS) or with emerging disease-modifying approaches such as gene therapy, neurotrophic factor delivery, and biomaterial scaffolds. These synergistic opportunities are discussed in the following Section (3.9).

### 3.9 Synergistic strategies combining cell therapy with established and emerging Parkinson's disease treatments

The future of PD therapy increasingly emphasizes multimodal approaches that combine cell-based transplantation with established symptomatic treatments and emerging disease-modifying technologies. Rather than standalone interventions, cell therapies are most effective as components within a broader therapeutic ecosystem aimed at enhancing graft viability, accelerating functional recovery, and extending therapeutic durability across PD's heterogeneous and progressive landscape.

Pharmacological support remains essential. l-DOPA, the cornerstone of symptomatic management, can aid motor function and facilitate rehabilitation during the latency period before grafted DA neurons establish robust connectivity and DA release^216^. Dose and duration must be carefully titrated, as excessive stimulation, particularly in the presence of serotonergic contamination within the graft, can induce graft-induced dyskinesias^217^. Personalized regimens, informed by graft maturity, host responsiveness, and dyskinesia profiles, are therefore critical.

DBS, targeting the subthalamic nucleus or globus pallidus interna, provides robust control of motor symptoms in advanced PD^218^ and may complement cell therapy through synergistic mechanisms. Next-generation DBS systems with sensing capabilities can detect real-time electrophysiological biomarkers of graft activity, enabling adaptive stimulation. Preclinical models show that closed-loop DBS aligned with pathological oscillations improves outcomes^219^, while clinical studies demonstrate that gamma oscillations in the subthalamic nucleus or motor cortex reflect DA tone and symptom severity, guiding adaptive DBS protocols^220^. These approaches illustrate the translational potential of biomarker-driven neuromodulation to support graft integration and long-term efficacy.

Preclinical work underscores that the electrophysiological milieu critically shapes graft outcomes. Building on the integration mechanisms discussed in Section 3.7, hiPSC-derived midbrain DA neurons transplanted into the SN of 6-OHDA-lesioned RAG2-KO mice displayed stable DA-like firing and circuit incorporation^146^. Intranigral graft placement more closely restores physiological nigrostriatal circuitry than intrastriatal delivery, and DBS may help establish a permissive network environment for graft survival and functional integration by normalizing aberrant basal ganglia oscillations.

Gene therapy offers another complementary avenue. Viral vectors encoding DA-synthesizing enzymes (TH, l-amino acid decarboxylase) or neurotrophic factors (cerebral DA neurotrophic factor (CDNF), GDNF, neurturin) can promote axonal sprouting, enhance TH expression, and increase striatal DA tone^221^, supporting graft survival, maturation, and integration of transplanted DA neurons.

Bioengineering strategies are increasingly applied to improve graft outcomes. Co-transplantation of DA progenitors with supportive cell types such as MSCs or CB glomus cells within engineered biomaterials (hydrogels, nanofibrous scaffolds) enhances cell retention, nutrient diffusion, axonal growth, synaptogenesis, and local immune modulation^63^, ^64^, ^65^. Controlled release of bioactive agents mitigates neuroinflammation and reduces dependence on systemic immunosuppression. For example, electroconductive self-healing hydrogels scavenge ROS and rescue ∼88% of inflamed neural stem cells *in vitro*, while alleviating neuroinflammation and neurodegeneration in 6-OHDA rat models^222^.

These combinatorial approaches not only improve graft viability and circuit reconstruction but also enable patient-specific strategies. Factors such as disease stage, biological age, immune competence, and comorbidities should guide regimen design to align therapeutic intensity with individual risk profiles and optimal treatment windows. Collectively, these strategies mark a shift toward integrative neurorestoration, embedding cell therapy within coordinated pharmacological, neuromodulatory, genetic, and biomaterial-based interventions. Future studies should focus on systematic evaluation of multimodal protocols, supported by longitudinal biomarker tracking and adaptive trial designs tailored to the dynamic needs of PD patients.

While these combinations can optimize graft survival, integration, and function, long-term success ultimately depends on the resilience of transplanted cells within the hostile PD brain. Among the earliest and most pervasive stressors is mitochondrial dysfunction, which compromises both native and grafted neurons. The following section explores mitochondrial biology in PD, detailing how bioenergetic deficits, altered dynamics, and impaired quality control intersect with cell therapy outcomes and represent critical therapeutic targets.

## 4 Mitochondrial dysfunction in Parkinson's disease: A central pathomechanism with therapeutic implications

The selective vulnerability of DA neurons in PD arises from intrinsic features such as extensive axonal arborization, high metabolic demand, limited antioxidant capacity, and disrupted calcium homeostasis^85^^,^^223^. Among the many intersecting pathogenic processes, mitochondrial dysfunction emerges as a central contributor, supported by strong evidence from both familial and sporadic PD^224^, ^225^, ^226^. Mitochondrial abnormalities encompass impaired energy production, altered dynamics and trafficking, and defective quality control mechanisms, including mitophagy. Many of these deficits manifest prior to overt neurodegeneration, implicating mitochondrial dysfunction in both disease onset and progression^227^.

Genetic studies further underscore the role of mitochondria in PD pathogenesis. Mutations in PD-associated genes, such as *SNCA*, leucine-rich repeat kinase 2 (*LRRK2*), parkin (*PRKN*), PTEN-induced kinase 1 (*PINK1*), Parkinson protein 7 (*PARK7*), and the glucocerebrosidase gene (*GBA*) disrupt mitochondrial homeostasis *via* diverse mechanisms^228^. For instance, *α*-SYN exerts mitochondrial toxicity through its oligomeric forms. These oligomers associate with mitochondrial membranes, impair oxidative phosphorylation, reduce ATP production, and trigger cellular stress, ultimately driving neuronal dysfunction and death^229^.

Taken together, converging genetic, biochemical, and environmental evidence positions mitochondrial dysfunction at the core of PD pathogenesis, highlighting mitochondria as a critical target for therapeutic intervention.

### 4.1 Bioenergetic failure and complex I deficiency

A defining biochemical hallmark of PD is the selective impairment of mitochondrial complex I (NADH:ubiquinone oxidoreductase), the first and largest enzyme of the electron transport chain. This deficiency is consistently observed in postmortem brain tissue from PD patients, most prominently in the SN, but also in extranigral regions such as the frontal cortex and putamen^230^, ^231^, ^232^. Similar downregulation of complex I has been reported in peripheral tissues, including skeletal muscle, platelets, lymphocytes, and skin fibroblasts^233^, ^234^, ^235^. These deficits appear independent of aging, disease duration, or neuronal loss, indicating a primary pathogenic role. Notably, they are not associated with mitochondrial DNA mutations, deletions, or copy number changes, pointing instead to post-transcriptional regulatory defects or functional inhibition.

Reduced complex I limits ATP synthesis and disrupts the NAD^+^/NADH ratio^236^, resulting in electron accumulation, enhanced ROS production (particularly superoxide at complexes I and III) and oxidative damage to lipids, proteins, and nucleic acids. This contributes to a self-perpetuating cycle of mitochondrial dysfunction, redox imbalance, and neuronal injury^224^. Additionally, oxidative stress disrupts mitochondrial dynamics and impairs mitophagy, worsening energy deficits and triggering neurodegeneration through apoptotic and necrotic pathways.

Neurotoxin models further support this mechanism. Agents such as MPTP and rotenone, which selectively inhibit complex I, reproduce several PD features, including nigrostriatal DA neuron loss, LB-like inclusions, motor deficits, and glial activation^237^^,^^238^. These models remain essential for investigating PD pathomechanisms and evaluating potential therapeutic strategies, underscoring the centrality of complex I dysfunction in PD pathogenesis.

### 4.2 Altered mitochondrial dynamics: fusion and fission imbalance

Mitochondria maintain cellular and bioenergetic homeostasis through continuous cycles of fusion, fission, and mitophagy, processes that regulate organelle morphology, size, shape, and intracellular distribution, predominantly critical in neurons with polarized architecture and high energy demands. Fusion promotes mitochondrial integrity and content exchange and is regulated by the GTPases mitofusin 1/2 (MFN1/2) on the outer mitochondrial membrane (OMM) and optic atrophy 1 (OPA1) on the inner membrane^239^.

Conversely, fission segregates damaged mitochondria and facilitates quality control *via* mitophagy. It is mediated by outer membrane adaptors, including mitochondrial fission factor, fission protein 1 (FIS1), and mitochondrial dynamics proteins 49 and 51, which recruit the cytosolic GTPase dynamin-related protein 1 (DRP1). DRP1 oligomerizes at fission sites to constrict and divide the mitochondrial membrane^240^^,^^241^. While fission is essential for organelle distribution and quality control, excessive fragmentation is linked to mitochondrial dysfunction and impaired biogenesis^242^. Fusion and fission are finely regulated by post-translational modifications, including ubiquitination, phosphorylation, acetylation, *S*-nitrosylation, and SUMOylation, allowing mitochondria to adapt to cellular stress^243^.

Balanced mitochondrial dynamics are crucial for neuronal function, influencing synaptic development, presynaptic maturation, dendritic spine formation, and synaptic transmission^244^. Dysregulation of these processes has been increasingly implicated in neurodegenerative diseases, including PD. Knockdown of MFN2 in hiPSCs impairs neurogenesis and synaptogenesis^245^, while DRP1 ablation in *Drosophila* disrupts mitochondrial trafficking to synapses, causing synaptic dysfunction^246^.

Indicators of mitochondrial fusion impairment in the inner membrane, including protein marker alterations and electron-dense deposits, were observed in both sporadic PD SN samples and cellular PD models^247^. Fibroblasts from individuals with biallelic OPA1 mutations displayed extensive mitochondrial fragmentation and disrupted network organization^248^. Likewise, MFN2 deletion in murine models resulted in severe defects in mitochondrial morphology, respiration, and intracellular localization, accompanied by progressive degeneration of the nigrostriatal DA pathway^249^^,^^250^.

Pharmacological inhibition of DRP1 with dysanore restored mitochondrial homeostasis and activated PINK1/Parkin-mediated mitophagy in MPTP-treated mice, leading to neuroprotection and improved functional outcomes^251^. These findings highlight the therapeutic potential of targeting fusion and fission balance to counteract neurodegeneration in PD.

### 4.3 Impaired mitochondrial axonal transport

Axonal transport is essential for neuronal homeostasis, enabling bidirectional trafficking of proteins, lipids, organelles, and synaptic vesicles along the axon. This process relies on ATP-driven motor proteins along microtubules. Kinesins mediate anterograde transport toward the axon terminal, whereas cytoplasmic dyneins facilitate retrograde transport back to the soma for recycling and degradation. Kinesins are heterotetrameric complexes composed of two kinesin heavy chains (KHC) and two light chains (KLC), with activity regulated *via* post-translational modifications^252^^,^^253^. Dynein function is similarly modulated through phosphorylation of dynein intermediate chains by kinases such as casein kinase^252^^,^^254^.

Axonal transport involves both fast-moving cargoes, like mitochondria and vesicles, and slower elements, such as cytoskeletal proteins, all vital for neuronal viability and synaptic transmission. Mitochondria are especially critical due to neurons’ high energy demands and polarized architecture. Mitochondrial movement is primarily microtubule-based, coordinated by the OMM GTPase Miro and adaptor proteins Milton (Trak1/Trak2). Disruption of Miro or Milton impaired anterograde transport and caused abnormal mitochondrial distribution in *Drosophila* models^137^^,^^255^. Miro knockdown also diminished retrograde motility *in vitro*^256^. At presynaptic terminals, mitochondria are anchored by syntaphilin, which interacts with dynein and the neuron-specific kinesin KIF5A^257^.

Dysregulation of axonal transport contributes to mitochondrial dysfunction and neurodegeneration in PD. In early-stage idiopathic PD (Hoehn and Yahr stages I–II), decreased KHC and KLC1 levels were observed in SN DA neurons, correlating with disease severity and preceding neuronal loss^258^. Dynein reductions occur predominantly in late-stage PD (Hoehn and Yahr stages IV–V), suggesting a temporal progression of transport impairment, beginning with early anterograde mitochondrial disruption followed by retrograde deficits. Efficient mitochondrial trafficking is therefore critical for neuronal survival, positioning axonal transport dysfunction as an early and progressive pathogenic mechanism in PD.

### 4.4 Defective mitophagy and mitochondrial quality control

Mitophagy, a selective form of autophagy, maintains mitochondrial quality by targeting damaged or residual mitochondria for lysosomal degradation. This preserves bioenergetic efficiency, limits ROS accumulation, and supports neuronal viability. Dysfunctional mitochondria are typically engulfed by double-membraned autophagosomes, which fuse with lysosomes for enzymatic degradation. When lysosomal function is compromised, alternative clearance pathways, such as extracellular vesicle release, may compensate^259^.

The PINK1/Parkin pathway is the primary regulator of stress-induced mitophagy. Under normal conditions, PINK1 is imported into healthy mitochondria and rapidly degraded. Upon mitochondrial depolarization, full-length PINK1 accumulates on the OMM, phosphorylating ubiquitin and the E3 ubiquitin ligase Parkin, which facilitates Parkin recruitment from the cytosol. Activated Parkin ubiquitinates various OMM proteins, including MFNs and voltage-dependent anion-selective channel 1, marking damaged mitochondria for autophagic clearance^260^^,^^261^. Ubiquitin chains are recognized by autophagic adaptors such as p62/SQSTM1, mediating their incorporation into LC3-positive autophagosomes.

Besides mitophagy initiation, the PINK1/Parkin pathway regulates mitochondrial biogenesis and morphology. Parkin-mediated degradation of the transcriptional repressor PARIS (ZNF746) upregulates PGC-1*α*, a master regulator of mitochondrial biogenesis^262^. Parkin also ubiquitinates MFN1 and MFN2, promoting fission through enhanced DRP1 recruitment, facilitating segregation of damaged mitochondrial fragments for degradation^261^.

Although extensively studied in stressed immortalized cells, PINK1/Parkin-mediated mitophagy in mature neurons remains less clear. *In vivo* studies in *Drosophila* and mammalian brains report limited Parkin translocation to mitochondria under basal conditions, suggesting reliance on alternative mitophagy pathways^263^^,^^264^. In DA neurons, mitophagy likely occurs predominantly in distal axons, which may elude bulk tissue analyses^265^. Deletion of PINK1 or Parkin in mice did not lead to mitochondrial accumulation but instead reduced mitochondrial mass and size^266^^,^^267^, indicating broader roles in turnover and biosynthesis.

While PINK1 and Parkin mutations account for a small subset of familial PD, growing evidence implicates pathway dysregulation in sporadic PD. Postmortem studies of PD and diffuse LB brains, as well as MPTP models, revealed *S*-nitrosylation and reduced Parkin activity under oxidative stress^268^. Fibroblasts from PINK1 mutation carriers exhibited impaired mitochondrial respiration and membrane potential, recapitulating features of late-onset PD^269^. Similarly, patient-derived iPSC models showed defective Parkin recruitment, increased mitochondrial DNA content, and abnormal mitochondrial biogenesis in DA neurons with PINK1 mutations^270^. Immunohistochemistry also revealed PINK1 colocalization with LBs in 5%–10% of brainstem neurons from heterozygous PINK1 mutation carriers^271^, suggesting partial involvement in idiopathic PD.

These findings underscore defective mitophagy and impaired mitochondrial quality control as key contributors to PD pathogenesis. Therapeutic strategies that enhance PINK1/Parkin signaling, restore lysosomal function, or modulate mitochondrial turnover hold promise for disease modification.

### 4.5 Mitochondrial integrity in cell-based replacement therapies

Emerging regenerative strategies increasingly recognize mitochondrial health as critical for therapeutic success. Cell-based replacement interventions using DA neurons derived from pluripotent stem cells aim to restore the nigrostriatal pathway, but graft survival, integration, and functional maturation depend heavily on maintaining mitochondrial integrity, dynamics, and bioenergetic capacity.

Postmortem analyses of patients up to 14 years after fVM transplantation showed elevated immunoreactivity for Tom20 (an OMM marker) in grafted neurons relative to host cells^272^. This correlated with healthy mitochondrial distribution across neuronal somata and axons, consistent with physiological DA neuron architecture. Similarly, autologous transplantation of hiPSC-derived DA neurons into the putamen of non-human primates revealed morphologically intact mitochondria in both cell bodies and neurites, without fragmentation or pathological clustering^72^.

Intercellular mitochondrial transfer further supports graft viability and host neuron survival. Stereotaxic delivery of MSCs enabled transfer of healthy mitochondria to damaged host cells *via* tunneling nanotubes, enhancing mitochondrial function and survival^273^. Furthermore, co-culture studies showed that hiPSC-derived astrocytes transfer mitochondria to DA neurons through a tunneling nanotube-independent, p38 MAPK-dependent mechanism^71^. Astrocyte-derived mitochondria confer resistance to rotenone-induced mitochondrial toxicity, highlighting neuroprotective potential.

These findings highlight mitochondria's dual role in PD, serving as vulnerable targets of pathology and as active mediators of repair in cell-based therapies. Maintaining mitochondrial health, appropriate intracellular distribution, and competence for intercellular transfer in transplanted neurons is essential for effective integration and sustained functional recovery. Interventions aimed at optimizing mitochondrial quality control, such as pharmacological preconditioning, genetic enhancement, or co-transplantation with mitochondrial donor cells, may further enhance graft performance and therapeutic efficacy.

Future investigations should prioritize elucidating the molecular mechanisms governing mitochondrial dynamics, inter-organelle communication, and intercellular mitochondrial transfer within the transplantation environment. A deeper mechanistic understanding will guide the development of next-generation cell therapies that leverage mitochondrial regenerative capacity to more effectively counteract PD.

Because mitochondrial stress and bioenergetic deficits undermine both native and transplanted neuron survival and function, emerging therapeutic paradigms increasingly incorporate supportive strategies to mitigate these vulnerabilities. The following section will examine paracrine and neuroprotective mechanisms, including exosome-mediated signaling, neurotrophic factor delivery, and immune modulation, that promote graft viability and functional integration in the PD brain.

## 5 Molecular and supportive signaling pathways in cell-based regeneration for Parkinson's disease

**Figure 6:**
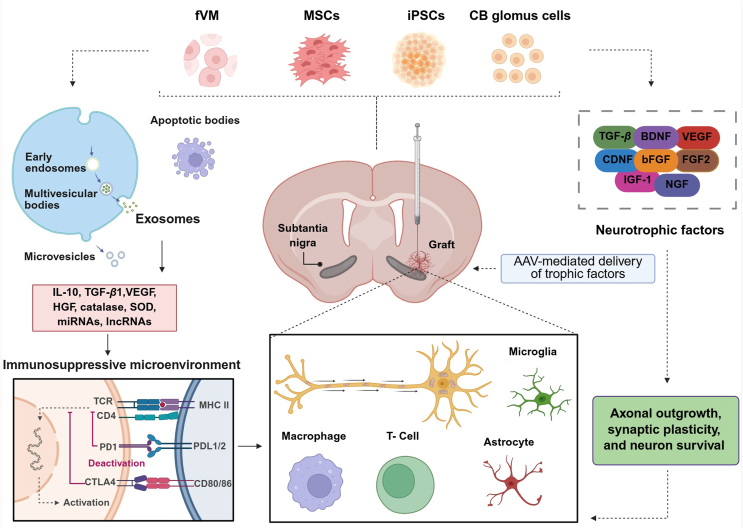
Therapeutic mechanisms of cell-based regeneration in Parkinson's disease. Transplanted cells promote repair through multiple complementary mechanisms. Extracellular vesicles, including exosomes, microvesicles, and apoptotic bodies, deliver anti-inflammatory cytokines, trophic molecules, and regulatory RNAs that support neuronal survival and help establish an immunosuppressive microenvironment conducive to graft acceptance. Concurrently, neurotrophic factors, either secreted by grafted cells or delivered *via* viral vectors, enhance axonal outgrowth, synaptic plasticity, and dopaminergic neuron survival. Together, these interactions between graft-derived signals and host neural, glial, and immune cells create an integrated regenerative network that underpins the therapeutic potential of cell-based strategies in Parkinson's disease.

While the preceding sections have examined the challenges of DA neuron loss and the limitations of conventional replacement approaches, it is increasingly clear that the benefits of cell-based therapies in PD extend beyond simple cell substitution. Transplanted cells can influence disease progression through three complementary mechanisms: release of extracellular vesicles carrying regulatory cargo, secretion of neurotrophic factors that support neuronal survival and plasticity, and modulation of the host neuroimmune environment to enhance graft acceptance and repair (Fig. 6). Together, these processes form a multifaceted regenerative network that underpins the therapeutic potential of next-generation cell-based strategies. The following subsections examine each mechanism in detail.

### 5.1 Therapeutic potential of exosomes in cell replacement strategies

Extracellular vesicles, including exosomes, microvesicles, and apoptotic bodies, were secreted by diverse cell types and classified based on size, biogenesis, and molecular composition. Exosomes, the smallest subset (30–150 nm), originated from the endosomal pathway *via* inward budding of multivesicular bodies, which subsequently fused with the plasma membrane to release their cargo extracellularly^274^. They were abundant in body fluids and carried a broad repertoire of bioactive molecules, including proteins, lipids, and various RNA species, reflecting the cell of origin^275^.

Exosome biogenesis was primarily orchestrated by the endosomal sorting complex required for transport (ESCRT) machinery: ESCRT-0 clustered ubiquitinated cargo, ESCRT-I and -II drove membrane invagination, and ESCRT-III catalyzed vesicle scission with the assistance of vacuolar protein sorting 4 ATPase^276^. ESCRT-independent mechanisms, such as tetraspanin-enriched microdomains and phosphatidylserine-mediated budding, also contributed. Surface proteins, including tetraspanins (CD9, CD63, CD81), lysosomal-associated membrane proteins, MHC molecules, integrins, annexins, and Rab GTPases, facilitated targeting, trafficking, and interactions with recipient cells^277^.

Functionally, exosomes were shown to mediate intercellular communication by delivering regulatory molecules, particularly miRNAs, that modulate gene expression and influence PD pathology^278^^,^^279^. Pathological *α*-SYN was detected in cerebrospinal fluid exosomes from individuals with PD and dementia with LBs, implicating vesicles in toxic protein propagation^280^^,^^281^. *α*-SYN itself impaired exosome biogenesis by downregulating ESCRT components, exacerbating protein accumulation and spread^282^. Age-related microglial dysfunction further increased the release of *α*-SYN-containing exosomes, whereas inhibiting microglial activation or vesicle secretion suppressed *α*-SYN transmission and neurodegeneration in PD models^280^^,^^283^.

Several PD-related genes have been reported to intersect with exosomal pathways. Mutations in *ATP13A2* impaired lysosomal function and hindered *α*-SYN degradation, whereas *ATP13A2* overexpression facilitated *α*-SYN clearance through vesicle release^284^. *LRRK2* mutations disrupted autophagy and exosome trafficking, and elevated neuronal exosomal L1 cell adhesion molecule correlated with LRRK2 pathology and may serve as a biomarker^285^^,^^286^.

Exosomal miRNAs were also shown to modulate PD-relevant pathways. MiR-7, often downregulated in PD, targeted *SNCA* mRNA to inhibit *α*-SYN expression, attenuating its propagation and neuroinflammation in experimental models^287^^,^^288^. MiR-4639-5p, enriched in neuronal exosomes, downregulated *DJ-1*, increasing oxidative stress and DA neuron vulnerability. Its levels were epigenetically regulated by histone deacetylase 11^289^^,^^290^. MiR-137 suppressed oxidation resistance 1 (*OXR1*), a key antioxidant gene, and its inhibition (or *OXR1* restoration) mitigated oxidative injury, improved motor function, and enhanced neuronal survival in PD models. Blocking exosomal miR-137 similarly mitigated oxidative damage and rescued pathological features^291^.

MSC-derived exosomes are of particular interest because they can cross the blood–brain barrier, have low immunogenicity, and remain stable in circulation^70^. Their cargo includes anti-inflammatory cytokines (interleukin (IL)-10 and TGF-*β*1), growth factors (vascular endothelial growth factor, VEGF) and hepatocyte growth factor, HGF), neuroprotective enzymes (catalase and superoxide dismutase), and regulatory RNAs (miRNAs and lncRNAs). These components promote neuronal survival, modulate immune responses, protect against complement-mediated cytotoxicity, and enhance angiogenesis, neurogenesis, and axonal outgrowth^70^^,^^277^.

In PD models, human adipose-derived stem cell exosomes improve apomorphine-induced rotational behavior, preserve SN DA neurons, and reduce microglial activation^69^. Human umbilical cord MSC (hUC-MSC) exosomes restore TH levels, suppress glial activation, and improve motor function in MPTP-injected mice^292^. Dental pulp stem cell- and MSC-derived vesicles protect against DA neuron loss and vascular dysfunction, with BM-MSC secretomes showing similar benefits that are largely attributable to their exosomal fraction^293^, ^294^, ^295^.

hUC-MSC-derived exosomes, which exhibit high proliferative and self-renewal capacity, improve neuron survival and DA content in SH-SY5Y cells and PD animal models^296^^,^^297^. These effects are partly mediated by the transfer of neuroprotective miRNAs such as miR-100a-5p, miR-133b, and miR-188-3p^298^, ^299^, ^300^. Therapeutic strategies using miRNA mimics (miR-124, miR-7) or antagomiRs (miR-155, miR-126) further modulate inflammasome activity, reduce *α*-SYN aggregation, and exert anti-inflammatory and neuroprotective effects^301^. Despite these promising preclinical findings, exosome-based diagnostics and therapies remain in early clinical development.

### 5.2 Neurotrophic factor signaling in cell-based regenerative medicine

Beyond vesicle-mediated communication, transplanted cells secrete neurotrophic factors such as BDNF, CDNF, GDNF, insulin-like growth factor 1, FGF2, nerve growth factor, TGF-*β*, and VEGF^172^^,^^174^. These molecules support axonal growth, synaptic remodeling, neuronal survival, and immunomodulation by suppressing microglial activation and promoting tissue repair.

Poor graft survival in trophic-deficient or immune-reactive environments remain a major obstacle^302^. Strategies to enhance neurotrophic factor signaling include pretreating donor cells with neurotrophins, co-transplanting cells engineered to secrete neurotrophic factors, and delivering exogenous factors *via* viral vectors, encapsulation devices, or hydrogels^303^, ^304^, ^305^. For instance, combining GDNF with fVM grafts improves DA neuron survival and integration in rodents and primates, while encapsulated GDNF-producing cells protect both host and graft^65^^,^^306^. Similarly, CDNF- or GDNF-releasing microcarriers promote graft maturation and axonal extension in hemiparkinsonian rats^307^.

hiPSC-derived grafts highlight the importance of trophic support. Patient-derived fibroblasts and iPSCs exhibited dysregulated neurotrophic factor and receptor expression during DA differentiation^308^. Co-transplantation of hiPSC-derived PITX3-eGFP^+^ fVM progenitors with adeno-associated viral vector-mediated GDNF delivery into the SN or striatum improved graft integration, restored DA levels, and enhanced behavioral outcomes in 6-OHDA rats, with effects correlating to GDNF distribution^309^. In MPTP-lesioned primates, adeno-associated viral vector encoding GDNF promoted axonal extension from human fetal neural stem cells toward the striatum, although most cells failed to mature into DA neurons, indicating that regional cues were also required^310^.

MSCs naturally provide robust trophic support and could be engineered for enhanced output. hUC-MSCs overexpressing VEGF, FGF2, or BDNF increased neuronal differentiation, reduced glial activation, and improved neuroprotection in PD models^311^, ^312^, ^313^. Similarly, BM-MSCs engineered to produce BDNF or CDNF preserved DA markers and recovered motor function in 6-OHDA rats^314^^,^^315^. Preconditioning BM-MSCs with basic FGF or modifying them to co-express GDNF and TH further increased DA levels, enhanced motor outcomes, and protected against MPTP-induced damage in monkeys^316^^,^^317^.

CB-derived cells, which secreted high levels of GDNF, provide paracrine neuroprotection without directly replacing DA neurons. CB transplantation stimulated endogenous nigrostriatal regeneration, promoted axonal sprouting, and yielded sustained clinical improvements in PD patients^183^^,^^318^.

### 5.3 Neuroimmunomodulatory considerations in cell-based approaches for Parkinson's disease

The central nervous system immune landscape plays a pivotal role in graft survival and integration. Allogeneic grafts, including unmatched fVM tissue or iPSC-derived neurons, usually require systemic immunosuppression to prevent rejection^302^^,^^319^^,^^320^. However, long-term immunosuppressive regimens carry significant risks, and even autologous or HLA-matched iPSC-derived neurons can trigger immune responses due to mitochondrial or epigenetic incompatibilities^68^^,^^321^, ^322^, ^323^, ^324^. Immune-engineered iPSCs with HLA deletion or checkpoint ligand overexpression (programmed death-ligand 1, CD47) were developed to evade immune surveillance and prolong graft survival^325^^,^^326^.

Certain donor cell types, mainly MSCs and CB-derived cells, exert intrinsic immunoregulatory effects by secreting IL-10, TGF-*β*, neurotrophic factors, and extracellular vesicles that suppress microglial activation, reprogram macrophages, and recruit regulatory T cells, mechanisms that indirectly support DA neuron regeneration^189^^,^^190^^,^^327^. CB-derived glomus cells additionally release pro-inflammatory cytokines (IL-1*β*, IL-6, tumor necrosis factor *α*), which influence neuronal excitability and host-graft interactions^327^.

MSC-related immunomodulation could be further amplified through preconditioning or genetic modification. FGF2 reduced chemokine CCL11 in dental pulp-derived MSCs *via* c-Jun N-terminal kinase signaling^328^. Hypoxia-preconditioned human olfactory mucosa MSCs secreted TGF-*β*1, enhancing DA mitochondrial function through microglial ALK/PI3K/Akt signaling, with Phase I trials confirming the safety of intraspinal transplantation in PD^329^. Preconditioning MSCs with *α*-SYN promoted stemness, glycolysis, and autophagy-related miRNA profiles, activating lysosomal/autophagic pathways that increased DA neuron survival and potentially countered *α*-SYN toxicity^61^.

To limit systemic immunosuppression, localized strategies have been developed, such as microparticle-based sustained-release tacrolimus (FK506), immunomodulatory hydrogels, and semi-permeable encapsulation devices that create restricted immune privilege^66^^,^^330^. These approaches are particularly relevant in PD, where chronic systemic immunosuppression is undesirable.

In summary, effective regenerative strategies need to couple neuronal replacement with precise immune modulation. Future approaches are likely to combine immune-shielding biomaterials, co-transplantation of immunoregulatory cells, and bioactive scaffolds to optimize graft survival, integration, and functional recovery.

## 6 Conclusions and future perspectives

Despite significant advances in symptomatic management, PD remains incurable. This highlights the urgent need for regenerative strategies that go beyond simple DA replacement. Long-lasting l-DOPA therapy is often complicated by motor fluctuations and dyskinesias. DBS and ablative procedures provide symptomatic relief but remain palliative, as they do not alter the underlying neurodegenerative process. These limitations emphasize the critical need for disease-modifying interventions.

Cell-based approaches have emerged as a promising avenue, encompassing fVM transplantation, iPSC-derived grafts, supportive MSCs, and CB glomus cells. These procedures show potential to reconstruct DA circuits, restore striatal innervation, and provide neuroprotective effects. Nevertheless, clinical translation faces significant challenges, including immunological rejection, heterogeneity in graft composition, and uncertainties about long-term functional integration and safety.

Future research should focus on promoting graft survival and function by combining cellular engineering, immune modulation, and biomaterial-based precision delivery. Incorporating disease-relevant markers such as synaptic pathology, mitochondrial dysfunction, and neuroinflammation may improve patient stratification and prognostic accuracy. Additionally, standardized protocols and robust regulatory frameworks will be essential to ensure safety, reproducibility, and scalability.

By integrating advances in molecular neuroscience, regenerative medicine, and neurotechnologies such as neuroimaging and electrophysiology, next-generation treatments could shift the paradigm from symptomatic management to true neuroprotection and circuit-level repair. If these challenges are successfully addressed, cell-based regenerative interventions have the potential to transform the therapeutic landscape of PD, offering durable, disease-modifying benefits and improved long-term quality of life for patients.

## Author contributions

Victor Tapias designed and conceptualized the article. Ana Lizeth Padilla and Victor Tapias wrote the initial manuscript, prepared the tables, and contributed to revisions. Ana Lizeth Padilla, José L. Lanciego, and Victor Tapias prepared the figures. José L. Lanciego, Miquel Vila, Fernando de Castro, Fernando Rodríguez de Fonseca, Antonia Serrano, David Pozo, Javier Sáez-Valero, Lucía Núñez and Carlos Villalobos participated in the discussion and revision of the manuscript. All authors have read and agreed to the published version of the article.

## Declaration of generative AI and AI-assisted technologies in the manuscript preparation process

AI-assisted tools were employed exclusively to enhance the clarity and overall quality of the writing. All content was reviewed and edited by the authors, who take full responsibility. No scientific content was generated by AI; the manuscript reflects the authors’ original work.

## Conflicts of interest

The authors declare no conflicts of interest.

## References

[1] Kalia L.V., Lang A.E. (2015). Parkinson's disease. Lancet.

[2] Zhang C., Bo R., Zhou T., Chen N., Yuan Y. (2024). The raphe nuclei are the early lesion site of gastric alpha-synuclein propagation to the substantia nigra. Acta Pharm Sin B.

[3] Zhu Q., Song J., Chen J.Y., Yuan Z., Liu L., Xie L.M. (2023). Corynoxine B targets at HMGB1/2 to enhance autophagy for alpha-synuclein clearance in fly and rodent models of Parkinson's disease. Acta Pharm Sin B.

[4] Fusco G., De Simone A., Gopinath T., Vostrikov V., Vendruscolo M., Dobson C.M. (2014). Direct observation of the three regions in alpha-synuclein that determine its membrane-bound behaviour. Nat Commun.

[5] Lautenschlager J., Stephens A.D., Fusco G., Strohl F., Curry N., Zacharopoulou M. (2018). C-terminal calcium binding of alpha-synuclein modulates synaptic vesicle interaction. Nat Commun.

[6] Ludtmann M.H., Angelova P.R., Ninkina N.N., Gandhi S., Buchman V.L., Abramov A.Y. (2016). Monomeric alpha-synuclein exerts a physiological role on brain ATP synthase. J Neurosci.

[7] Grosso Jasutkar H., Oh S.E., Mouradian M.M. (2022). Therapeutics in the pipeline targeting alpha-synuclein for Parkinson's disease. Pharmacol Rev.

[8] Hassanzadeh K., Liu J., Maddila S., Mouradian M.M. (2024). Posttranslational modifications of alpha-synuclein, their therapeutic potential, and crosstalk in health and neurodegenerative diseases. Pharmacol Rev.

[9] Brundin P., Li J.Y., Holton J.L., Lindvall O., Revesz T. (2008). Research in motion: the enigma of Parkinson's disease pathology spread. Nat Rev Neurosci.

[10] Beal M.F., Chiluwal J., Calingasan N.Y., Milne G.L., Shchepinov M.S., Tapias V. (2020). Isotope-reinforced polyunsaturated fatty acids improve Parkinson's disease-like phenotype in rats overexpressing alpha-synuclein. Acta Neuropathol Commun.

[11] Tapias V., Hu X., Luk K.C., Sanders L.H., Lee V.M., Greenamyre J.T. (2017). Synthetic alpha-synuclein fibrils cause mitochondrial impairment and selective dopamine neurodegeneration in part *via* iNOS-mediated nitric oxide production. Cell Mol Life Sci.

[12] Zharikov A.D., Cannon J.R., Tapias V., Bai Q., Horowitz M.P., Shah V. (2015). shRNA targeting alpha-synuclein prevents neurodegeneration in a Parkinson's disease model. J Clin Investig.

[13] Beach T.G., Walker D.G., Sue L.I., Newell A., Adler C.C., Joyce J.N. (2004). Substantia nigra Marinesco bodies are associated with decreased striatal expression of dopaminergic markers. J Neuropathol Exp Neurol.

[14] Gala A.S., Wilkins K.B., Petrucci M.N., Kehnemouyi Y.M., Velisar A., Trager M.H. (2024). The digital signature of emergent tremor in Parkinson's disease. npj Parkinson's Dis.

[15] Grealish S., Jonsson M.E., Li M., Kirik D., Bjorklund A., Thompson L.H. (2010). The A9 dopamine neuron component in grafts of ventral mesencephalon is an important determinant for recovery of motor function in a rat model of Parkinson's disease. Brain.

[16] Cai X., Liu C., Tsutsui-Kimura I., Lee J.H., Guo C., Banerjee A. (2024). Dopamine dynamics are dispensable for movement but promote reward responses. Nature.

[17] Fraser K.M., Collins V., Wolff A.R., Ottenheimer D.J., Bornhoft K.N., Pat F. (2025). Contextual cues facilitate dynamic value encoding in the mesolimbic dopamine system. Curr Biol.

[18] Aarsland D., Batzu L., Halliday G.M., Geurtsen G.J., Ballard C., Ray Chaudhuri K. (2021). Parkinson disease-associated cognitive impairment. Nat Rev Dis Primers.

[19] Chaudhuri K.R., Healy D.G., Schapira A.H., National Institute for Clinical E. (2006). Non-motor symptoms of Parkinson's disease: diagnosis and management. Lancet Neurol.

[20] Durcan R., Wiblin L., Lawson R.A., Khoo T.K., Yarnall A.J., Duncan G.W. (2019). Prevalence and duration of non-motor symptoms in prodromal Parkinson's disease. Eur J Neurol.

[21] Pont-Sunyer C., Hotter A., Gaig C., Seppi K., Compta Y., Katzenschlager R. (2015). The onset of nonmotor symptoms in Parkinson's disease (the ONSET PD study). Mov Disord.

[22] Lee F.J., Pei L., Moszczynska A., Vukusic B., Fletcher P.J., Liu F. (2007). Dopamine transporter cell surface localization facilitated by a direct interaction with the dopamine D2 receptor. EMBO J.

[23] German C.L., Baladi M.G., McFadden L.M., Hanson G.R., Fleckenstein A.E. (2015). Regulation of the dopamine and vesicular monoamine transporters: pharmacological targets and implications for disease. Pharmacol Rev.

[24] Masato A., Plotegher N., Boassa D., Bubacco L. (2019). Impaired dopamine metabolism in Parkinson's disease pathogenesis. Mol Neurodegener.

[25] Dick F., Tysnes O.B., Alves G.W., Nido G.S., Tzoulis C. (2023). Altered transcriptome–proteome coupling indicates aberrant proteostasis in Parkinson's disease. iScience.

[26] Lind-Holm Mogensen F., Seibler P., Grunewald A., Michelucci A. (2025). Microglial dynamics and neuroinflammation in prodromal and early Parkinson's disease. J Neuroinflammation.

[27] Roodveldt C., Bernardino L., Oztop-Cakmak O., Dragic M., Fladmark K.E., Ertan S. (2024). The immune system in Parkinson's disease: what we know so far. Brain.

[28] Smajic S., Prada-Medina C.A., Landoulsi Z., Ghelfi J., Delcambre S., Dietrich C. (2022). Single-cell sequencing of human midbrain reveals glial activation and a Parkinson-specific neuronal state. Brain.

[29] Ahlskog J.E., Muenter M.D. (2001). Frequency of levodopa-related dyskinesias and motor fluctuations as estimated from the cumulative literature. Mov Disord.

[30] Aquino C.C., Fox S.H. (2015). Clinical spectrum of levodopa-induced complications. Mov Disord.

[31] Blanchet P.J., Allard P., Gregoire L., Tardif F., Bedard P.J. (1996). Risk factors for peak dose dyskinesia in 100 levodopa-treated parkinsonian patients. Can J Neurol Sci.

[32] Jankovic J., Tan E.K. (2020). Parkinson's disease: etiopathogenesis and treatment. J Neurol Neurosurg Psychiatry.

[33] Gharabaghi A., Cebi I., Leavitt D., Scherer M., Bookjans P., Brunnett B. (2024). Randomized crossover trial on motor and non-motor outcome of directional deep brain stimulation in Parkinson's disease. npj Parkinson's Dis.

[34] Jost S.T., Konitsioti A., Loehrer P.A., Ashkan K., Rizos A., Sauerbier A. (2023). Non-motor effects of deep brain stimulation in Parkinson's disease motor subtypes. Parkinsonism Relat Disord.

[35] Pagge C., Caballero-Insaurriaga J., Oliviero A., Foffani G., Ammann C. (2024). Transcranial static magnetic field stimulation of the supplementary motor area decreases corticospinal excitability in the motor cortex: a pilot study. Sci Rep.

[36] Dileone M., Ammann C., Catanzaro V., Pagge C., Piredda R., Monje M.H.G. (2022). Home-based transcranial static magnetic field stimulation of the motor cortex for treating levodopa-induced dyskinesias in Parkinson's disease: a randomized controlled trial. Brain Stimul.

[37] Harris J.P., Burrell J.C., Struzyna L.A., Chen H.I., Serruya M.D., Wolf J.A. (2020). Emerging regenerative medicine and tissue engineering strategies for Parkinson's disease. npj Parkinson's Dis.

[38] Alekseenko Z., Dias J.M., Adler A.F., Kozhevnikova M., van Lunteren J.A., Nolbrant S. (2022). Robust derivation of transplantable dopamine neurons from human pluripotent stem cells by timed retinoic acid delivery. Nat Commun.

[39] Brot S., Thamrin N.P., Bonnet M.L., Francheteau M., Patrigeon M., Belnoue L. (2022). Long-term evaluation of intranigral transplantation of human iPSC-derived dopamine neurons in a Parkinson's disease mouse model. Cells.

[40] Doi D., Magotani H., Kikuchi T., Ikeda M., Hiramatsu S., Yoshida K. (2020). Pre-clinical study of induced pluripotent stem cell-derived dopaminergic progenitor cells for Parkinson's disease. Nat Commun.

[41] Kikuchi T., Morizane A., Doi D., Magotani H., Onoe H., Hayashi T. (2017). Human iPS cell-derived dopaminergic neurons function in a primate Parkinson's disease model. Nature.

[42] Song B., Cha Y., Ko S., Jeon J., Lee N., Seo H. (2020). Human autologous iPSC-derived dopaminergic progenitors restore motor function in Parkinson's disease models. J Clin Investig.

[43] Tao Y., Vermilyea S.C., Zammit M., Lu J., Olsen M., Metzger J.M. (2021). Autologous transplant therapy alleviates motor and depressive behaviors in parkinsonian monkeys. Nat Med.

[44] Wianny F., Dzahini K., Fifel K., Wilson C.R.E., Bernat A., Dolmazon V. (2022). Induced cognitive impairments reversed by grafts of neural precursors: a longitudinal study in a macaque model of Parkinson's disease. Adv Sci (Weinh).

[45] Barker R.A., consortium T. (2019). Designing stem-cell-based dopamine cell replacement trials for Parkinson's disease. Nat Med.

[46] Li W., Englund E., Widner H., Mattsson B., van Westen D., Latt J. (2016). Extensive graft-derived dopaminergic innervation is maintained 24 years after transplantation in the degenerating parkinsonian brain. Proc Natl Acad Sci U S A.

[47] Mendez I., Sanchez-Pernaute R., Cooper O., Vinuela A., Ferrari D., Bjorklund L. (2005). Cell type analysis of functional fetal dopamine cell suspension transplants in the striatum and substantia nigra of patients with Parkinson's disease. Brain.

[48] Wenning G.K., Odin P., Morrish P., Rehncrona S., Widner H., Brundin P. (1997). Short- and long-term survival and function of unilateral intrastriatal dopaminergic grafts in Parkinson's disease. Ann Neurol.

[49] Sawamoto N., Doi D., Nakanishi E., Sawamura M., Kikuchi T., Yamakado H. (2025). Phase I/II trial of iPS-cell-derived dopaminergic cells for Parkinson's disease. Nature.

[50] Schweitzer J.S., Song B., Herrington T.M., Park T.Y., Lee N., Ko S. (2020). Personalized iPSC-derived dopamine progenitor cells for Parkinson's disease. N Engl J Med.

[51] Takahashi J. (2020). iPS cell-based therapy for Parkinson's disease: a Kyoto trial. Regen Ther.

[52] Arjona V., Minguez-Castellanos A., Montoro R.J., Ortega A., Escamilla F., Toledo-Aral J.J. (2003). Autotransplantation of human carotid body cell aggregates for treatment of Parkinson's disease. Neurosurgery.

[53] Minguez-Castellanos A., Escamilla-Sevilla F., Hotton G.R., Toledo-Aral J.J., Ortega-Moreno A., Mendez-Ferrer S. (2007). Carotid body autotransplantation in Parkinson disease: a clinical and positron emission tomography study. J Neurol Neurosurg Psychiatry.

[54] Pozo D. (2021). Cell-based drug delivery harnesses inflammatory and autoimmune responses in neurodegeneration. J Mol Med (Berl).

[55] Venkataramana N.K., Kumar S.K., Balaraju S., Radhakrishnan R.C., Bansal A., Dixit A. (2010). Open-labeled study of unilateral autologous bone-marrow-derived mesenchymal stem cell transplantation in Parkinson's disease. Transl Res.

[56] Han X., Wang M., Duan S., Franco P.J., Kenty J.H., Hedrick P. (2019). Generation of hypoimmunogenic human pluripotent stem cells. Proc Natl Acad Sci U S A.

[57] Kim J., Nam Y., Jeon D., Choi Y., Choi S., Hong C.P. (2025). Generation of hypoimmunogenic universal iPS cells through HLA-type gene knockout. Exp Mol Med.

[58] Hoffmann A.C., Minakaki G., Menges S., Salvi R., Savitskiy S., Kazman A. (2019). Extracellular aggregated alpha synuclein primarily triggers lysosomal dysfunction in neural cells prevented by trehalose. Sci Rep.

[59] Kallab M., Herrera-Vaquero M., Johannesson M., Eriksson F., Sigvardson J., Poewe W. (2018). Region-specific effects of immunotherapy with antibodies targeting alpha-synuclein in a transgenic model of synucleinopathy. Front Neurosci.

[60] Pagano G., Taylor K.I., Anzures Cabrera J., Simuni T., Marek K., Postuma R.B. (2024). Prasinezumab slows motor progression in rapidly progressing early-stage Parkinson's disease. Nat Med.

[61] Shin J.Y., Kim D.Y., Lee J., Shin Y.J., Kim Y.S., Lee P.H. (2022). Priming mesenchymal stem cells with alpha-synuclein enhances neuroprotective properties through induction of autophagy in Parkinsonian models. Stem Cell Res Ther.

[62] Zhang K., Zhu S., Li J., Jiang T., Feng L., Pei J. (2021). Targeting autophagy using small-molecule compounds to improve potential therapy of Parkinson's disease. Acta Pharm Sin B.

[63] Comini G., Kelly R., Jarrin S., Patton T., Narasimhan K., Pandit A. (2024). Survival and maturation of human induced pluripotent stem cell-derived dopaminergic progenitors in the parkinsonian rat brain is enhanced by transplantation in a neurotrophin-enriched hydrogel. J Neural Eng.

[64] Francis N.L., Zhao N., Calvelli H.R., Saini A., Gifford J.J., Wagner G.C. (2020). Peptide-based scaffolds for the culture and transplantation of human dopaminergic neurons. Tissue Eng Part A.

[65] Moriarty N., Cabre S., Alamilla V., Pandit A., Dowd E. (2019). Encapsulation of young donor age dopaminergic grafts in a GDNF-loaded collagen hydrogel further increases their survival, reinnervation, and functional efficacy after intrastriatal transplantation in hemi-Parkinsonian rats. Eur J Neurosci.

[66] Naaz A., Turnquist H.R., Gorantla V.S., Little S.R. (2024). Drug delivery strategies for local immunomodulation in transplantation: bridging the translational gap. Adv Drug Deliv Rev.

[67] Soldner F., Hockemeyer D., Beard C., Gao Q., Bell G.W., Cook E.G. (2009). Parkinson's disease patient-derived induced pluripotent stem cells free of viral reprogramming factors. Cell.

[68] Wei W., Gaffney D.J., Chinnery P.F. (2021). Cell reprogramming shapes the mitochondrial DNA landscape. Nat Commun.

[69] Chan L., Hsu W., Chen K.Y., Wang W., Hung Y.C., Hong C.T. (2023). Therapeutic effect of human adipocyte-derived stem cell-derived exosomes on a transgenic mouse model of Parkinson's disease. In Vivo (Athens).

[70] Xiao H., Yu X., Liu Y., Jiang W., Meng X., Dong Z. (2025). Mesenchymal stem cell-derived exosomes-a promising therapeutic approach to improve neurocognitive disorders in chronic obstructive pulmonary disease. Stem Cell Res Ther.

[71] Cheng X.Y., Biswas S., Li J., Mao C.J., Chechneva O., Chen J. (2020). Human iPSCs derived astrocytes rescue rotenone-induced mitochondrial dysfunction and dopaminergic neurodegeneration *in vitro* by donating functional mitochondria. Transl Neurodegener.

[72] Hallett P.J., Deleidi M., Astradsson A., Smith G.A., Cooper O., Osborn T.M. (2015). Successful function of autologous iPSC-derived dopamine neurons following transplantation in a non-human primate model of Parkinson's disease. Cell Stem Cell.

[73] Sasaki Y., Milbrandt J. (2010). Axonal degeneration is blocked by nicotinamide mononucleotide adenylyltransferase (Nmnat) protein transduction into transected axons. J Biol Chem.

[74] Knoferle J., Koch J.C., Ostendorf T., Michel U., Planchamp V., Vutova P. (2010). Mechanisms of acute axonal degeneration in the optic nerve *in vivo*. Proc Natl Acad Sci U S A.

[75] Koch J.C., Bitow F., Haack J., d'Hedouville Z., Zhang J.N., Tonges L. (2015). Alpha-Synuclein affects neurite morphology, autophagy, vesicle transport and axonal degeneration in CNS neurons. Cell Death Dis.

[76] Burke R.E., O'Malley K. (2013). Axon degeneration in Parkinson's disease. Exp Neurol.

[77] Moldovan M., Alvarez S., Krarup C. (2009). Motor axon excitability during Wallerian degeneration. Brain.

[78] Wang J.T., Medress Z.A., Barres B.A. (2012). Axon degeneration: molecular mechanisms of a self-destruction pathway. J Cell Biol.

[79] Wilson D.M., Cookson M.R., Van Den Bosch L., Zetterberg H., Holtzman D.M., Dewachter I. (2023). Hallmarks of neurodegenerative diseases. Cell.

[80] Matsuda W., Furuta T., Nakamura K.C., Hioki H., Fujiyama F., Arai R. (2009). Single nigrostriatal dopaminergic neurons form widely spread and highly dense axonal arborizations in the neostriatum. J Neurosci.

[81] Bolam J.P., Pissadaki E.K. (2012). Living on the edge with too many mouths to feed: why dopamine neurons die. Mov Disord.

[82] Gonzalez-Cabrera C., Meza R., Ulloa L., Merino-Sepulveda P., Luco V., Sanhueza A. (2017). Characterization of the axon initial segment of mice substantia nigra dopaminergic neurons. J Comp Neurol.

[83] Lammel S., Hetzel A., Hackel O., Jones I., Liss B., Roeper J. (2008). Unique properties of mesoprefrontal neurons within a dual mesocorticolimbic dopamine system. Neuron.

[84] Tarfa R.A., Evans R.C., Khaliq Z.M. (2017). Enhanced sensitivity to hyperpolarizing inhibition in mesoaccumbal relative to nigrostriatal dopamine neuron subpopulations. J Neurosci.

[85] Pacelli C., Giguere N., Bourque M.J., Levesque M., Slack R.S., Trudeau L.E. (2015). Elevated mitochondrial bioenergetics and axonal arborization size are key contributors to the vulnerability of dopamine neurons. Curr Biol.

[86] Damier P., Hirsch E.C., Agid Y., Graybiel A.M. (1999). The substantia nigra of the human brain. II. Patterns of loss of dopamine-containing neurons in Parkinson's disease. Brain.

[87] Kordower J.H., Olanow C.W., Dodiya H.B., Chu Y., Beach T.G., Adler C.H. (2013). Disease duration and the integrity of the nigrostriatal system in Parkinson's disease. Brain.

[88] Surmeier D.J., Obeso J.A., Halliday G.M. (2017). Selective neuronal vulnerability in Parkinson disease. Nat Rev Neurosci.

[89] Heng N., Malek N., Lawton M.A., Nodehi A., Pitz V., Grosset K.A. (2023). Striatal dopamine loss in early Parkinson's disease: systematic review and novel analysis of dopamine transporter imaging. Mov Disord Clin Pract.

[90] Houot M., Arnaud S., Mongin M., Pop G., Soussan M., Lannuzel A. (2024). Relevance of ^123^I-FP-CIT SPECT prescriptions for the diagnosis of parkinsonian syndromes. Sci Rep.

[91] Koros C., Simitsi A.M., Prentakis A., Papagiannakis N., Bougea A., Pachi I. (2020). DaTSCAN (^123^I-FP-CIT SPECT) imaging in early versus mid and late onset Parkinson's disease: longitudinal data from the PPMI study. Parkinsonism Relat Disord.

[92] Laruelle M., Baldwin R.M., Malison R.T., Zea-Ponce Y., Zoghbi S.S., al-Tikriti M.S. (1993). SPECT imaging of dopamine and serotonin transporters with [^123^I]beta-CIT: pharmacological characterization of brain uptake in nonhuman primates. Synapse.

[93] Morley V., Dolt K.S., Alcaide-Corral C.J., Walton T., Lucatelli C., Mashimo T. (2023). *In vivo*^18^F-DOPA PET imaging identifies a dopaminergic deficit in a rat model with a G51D alpha-synuclein mutation. Front Neurosci.

[94] Nurmi E., Ruottinen H.M., Bergman J., Haaparanta M., Solin O., Sonninen P. (2001). Rate of progression in Parkinson's disease: a 6-[^18^F]fluoro-L-dopa PET study. Mov Disord.

[95] Pavese N., Rivero-Bosch M., Lewis S.J., Whone A.L., Brooks D.J. (2011). Progression of monoaminergic dysfunction in Parkinson's disease: a longitudinal ^18^F-dopa PET study. Neuroimage.

[96] Freed C.R., Breeze R.E., Rosenberg N.L., Schneck S.A., Kriek E., Qi J.X. (1992). Survival of implanted fetal dopamine cells and neurologic improvement 12 to 46 months after transplantation for Parkinson's disease. N Engl J Med.

[97] Lindvall O., Brundin P., Widner H., Rehncrona S., Gustavii B., Frackowiak R. (1990). Grafts of fetal dopamine neurons survive and improve motor function in Parkinson's disease. Science.

[98] Matuskey D., Tinaz S., Wilcox K.C., Naganawa M., Toyonaga T., Dias M. (2020). Synaptic changes in Parkinson disease assessed with *in vivo* imaging. Ann Neurol.

[99] Chen Q., Andersen A.H., Zhang Z., Ovadia A., Cass W.A., Gash D.M. (1999). Functional MRI of basal ganglia responsiveness to levodopa in parkinsonian rhesus monkeys. Exp Neurol.

[100] Hernadi G., Pinter D., Nagy S.A., Orsi G., Komoly S., Janszky J. (2021). Fast 3 T nigral hyperintensity magnetic resonance imaging in Parkinson's disease. Sci Rep.

[101] Sulzer D., Cassidy C., Horga G., Kang U.J., Fahn S., Casella L. (2018). Neuromelanin detection by magnetic resonance imaging (MRI) and its promise as a biomarker for Parkinson's disease. npj Parkinson's Dis.

[102] Bannon M.J., Poosch M.S., Xia Y., Goebel D.J., Cassin B., Kapatos G. (1992). Dopamine transporter mRNA content in human substantia nigra decreases precipitously with age. Proc Natl Acad Sci U S A.

[103] Joyce J.N., Smutzer G., Whitty C.J., Myers A., Bannon M.J. (1997). Differential modification of dopamine transporter and tyrosine hydroxylase mRNAs in midbrain of subjects with Parkinson's, Alzheimer's with parkinsonism, and Alzheimer's disease. Mov Disord.

[104] Kastner A., Hirsch E.C., Agid Y., Javoy-Agid F. (1993). Tyrosine hydroxylase protein and messenger RNA in the dopaminergic nigral neurons of patients with Parkinson's disease. Brain Res.

[105] Miller G.W., Staley J.K., Heilman C.J., Perez J.T., Mash D.C., Rye D.B. (1997). Immunochemical analysis of dopamine transporter protein in Parkinson's disease. Ann Neurol.

[106] Pirooznia S.K., Rosenthal L.S., Dawson V.L., Dawson T.M. (2021). Parkinson disease: translating insights from molecular mechanisms to neuroprotection. Pharmacol Rev.

[107] Goetz C.G., Poewe W., Rascol O., Sampaio C., Stebbins G.T., Counsell C. (2004). Movement disorder society task force report on the Hoehn and Yahr staging scale: status and recommendations. Mov Disord.

[108] Hoehn M.M., Yahr M.D. (1967). Parkinsonism: onset, progression and mortality. Neurology.

[109] Holden S.K., Finseth T., Sillau S.H., Berman B.D. (2018). Progression of MDS-UPDRS scores over five years in *de novo* parkinson disease from the Parkinson's progression markers initiative cohort. Mov Disord Clin Pract.

[110] Huot P., Johnston T.H., Koprich J.B., Fox S.H., Brotchie J.M. (2013). The pharmacology of l-DOPA-induced dyskinesia in Parkinson's disease. Pharmacol Rev.

[111] Gallego I., Villate-Beitia I., Saenz-Del-Burgo L., Puras G., Pedraz J.L. (2022). Therapeutic opportunities and delivery strategies for brain revascularization in stroke, neurodegeneration, and aging. Pharmacol Rev.

[112] Bjorklund A., Dunnett S.B. (2007). Dopamine neuron systems in the brain: an update. Trends Neurosci.

[113] Bjorklund A., Stenevi U., Dunnett S.B., Iversen S.D. (1981). Functional reactivation of the deafferented neostriatum by nigral transplants. Nature.

[114] Fjodorova M., Torres E.M., Dunnett S.B. (2017). Transplantation site influences the phenotypic differentiation of dopamine neurons in ventral mesencephalic grafts in Parkinsonian rats. Exp Neurol.

[115] Goren B., Kahveci N., Eyigor O., Alkan T., Korfali E., Ozluk K. (2005). Effects of intranigral *vs* intrastriatal fetal mesencephalic neural grafts on motor behavior disorders in a rat Parkinson model. Surg Neurol.

[116] Nikkhah G., Cunningham M.G., Jodicke A., Knappe U., Bjorklund A. (1994). Improved graft survival and striatal reinnervation by microtransplantation of fetal nigral cell suspensions in the rat Parkinson model. Brain Res.

[117] Perlow M.J., Freed W.J., Hoffer B.J., Seiger A., Olson L., Wyatt R.J. (1979). Brain grafts reduce motor abnormalities produced by destruction of nigrostriatal dopamine system. Science.

[118] Bakay R.A., Fiandaca M.S., Barrow D.L., Schiff A., Collins D.C. (1985). Preliminary report on the use of fetal tissue transplantation to correct MPTP-induced Parkinson-like syndrome in primates. Appl Neurophysiol.

[119] Fine A., Hunt S.P., Oertel W.H., Nomoto M., Chong P.N., Bond A. (1988). Transplantation of embryonic marmoset dopaminergic neurons to the corpus striatum of marmosets rendered parkinsonian by 1-methyl-4-phenyl-1,2,3,6-tetrahydropyridine. Prog Brain Res.

[120] Sladek J.R., Collier T.J., Haber S.N., Roth R.H., Redmond D.E. (1986). Survival and growth of fetal catecholamine neurons transplanted into primate brain. Brain Res Bull.

[121] Kopyov O.V., Jacques D.S., Lieberman A., Duma C.M., Rogers R.L. (1997). Outcome following intrastriatal fetal mesencephalic grafts for Parkinson's patients is directly related to the volume of grafted tissue. Exp Neurol.

[122] Kordower J.H., Freeman T.B., Snow B.J., Vingerhoets F.J., Mufson E.J., Sanberg P.R. (1995). Neuropathological evidence of graft survival and striatal reinnervation after the transplantation of fetal mesencephalic tissue in a patient with Parkinson's disease. N Engl J Med.

[123] Ma Y., Tang C., Chaly T., Greene P., Breeze R., Fahn S. (2010). Dopamine cell implantation in Parkinson's disease: long-term clinical and ^18^F-FDOPA PET outcomes. J Nucl Med.

[124] Freed C.R., Greene P.E., Breeze R.E., Tsai W.Y., DuMouchel W., Kao R. (2001). Transplantation of embryonic dopamine neurons for severe Parkinson's disease. N Engl J Med.

[125] Olanow C.W., Goetz C.G., Kordower J.H., Stoessl A.J., Sossi V., Brin M.F. (2003). A double-blind controlled trial of bilateral fetal nigral transplantation in Parkinson's disease. Ann Neurol.

[126] Barker R.A., Lao-Kaim N.P., Guzman N.V., Athauda D., Bjartmarz H., Bjorklund A. (2025). The TransEuro open-label trial of human fetal ventral mesencephalic transplantation in patients with moderate Parkinson's disease. Nat Biotechnol.

[127] Kefalopoulou Z., Politis M., Piccini P., Mencacci N., Bhatia K., Jahanshahi M. (2014). Long-term clinical outcome of fetal cell transplantation for Parkinson disease: two case reports. JAMA Neurol.

[128] Kordower J.H., Dodiya H.B., Kordower A.M., Terpstra B., Paumier K., Madhavan L. (2011). Transfer of host-derived alpha synuclein to grafted dopaminergic neurons in rat. Neurobiol Dis.

[129] Kordower J.H., Styren S., Clarke M., DeKosky S.T., Olanow C.W., Freeman T.B. (1997). Fetal grafting for Parkinson's disease: expression of immune markers in two patients with functional fetal nigral implants. Cell Transplant.

[130] Nelke A., Garcia-Lopez S., Caso J.R., Pereira M.P. (2024). The therapeutic use of clonal neural stem cells in experimental Parkinson s disease. Stem Cell Res Ther.

[131] Cooper O., Astradsson A., Hallett P., Robertson H., Mendez I., Isacson O. (2009). Lack of functional relevance of isolated cell damage in transplants of Parkinson's disease patients. J Neurol.

[132] Kordower J.H., Chu Y., Hauser R.A., Freeman T.B., Olanow C.W. (2008). Lewy body-like pathology in long-term embryonic nigral transplants in Parkinson's disease. Nat Med.

[133] Kordower J.H., Chu Y., Hauser R.A., Olanow C.W., Freeman T.B. (2008). Transplanted dopaminergic neurons develop PD pathologic changes: a second case report. Mov Disord.

[134] Kurowska Z., Englund E., Widner H., Lindvall O., Li J.Y., Brundin P. (2011). Signs of degeneration in 12–22-year old grafts of mesencephalic dopamine neurons in patients with Parkinson's disease. J Parkinsons Dis.

[135] Li J.Y., Englund E., Holton J.L., Soulet D., Hagell P., Lees A.J. (2008). Lewy bodies in grafted neurons in subjects with Parkinson's disease suggest host-to-graft disease propagation. Nat Med.

[136] Ornelas A.S., Adler C.H., Serrano G.E., Curry J.R., Shill H.A., Kopyov O. (2020). Co-Existence of tau and alpha-synuclein pathology in fetal graft tissue at autopsy: a case report. Parkinsonism Relat Disord.

[137] Guo X., Macleod G.T., Wellington A., Hu F., Panchumarthi S., Schoenfield M. (2005). The GTPase dMiro is required for axonal transport of mitochondria to *Drosophila* synapses. Neuron.

[138] Kaur U., Lee J.C. (2020). Unroofing site-specific alpha-synuclein–lipid interactions at the plasma membrane. Proc Natl Acad Sci U S A.

[139] Zhang S., Liu Y.Q., Jia C., Lim Y.J., Feng G., Xu E. (2021). Mechanistic basis for receptor-mediated pathological alpha-synuclein fibril cell-to-cell transmission in Parkinson's disease. Proc Natl Acad Sci U S A.

[140] Chen K.S., Menezes K., Rodgers J.B., O'Hara D.M., Tran N., Fujisawa K. (2021). Small molecule inhibitors of alpha-synuclein oligomers identified by targeting early dopamine-mediated motor impairment in *C. elegans*. Mol Neurodegener.

[141] Horne R.I., Andrzejewska E.A., Alam P., Brotzakis Z.F., Srivastava A., Aubert A. (2024). Discovery of potent inhibitors of alpha-synuclein aggregation using structure-based iterative learning. Nat Chem Biol.

[142] Taguchi Y.V., Gorenberg E.L., Nagy M., Thrasher D., Fenton W.A., Volpicelli-Daley L. (2019). Hsp110 mitigates alpha-synuclein pathology *in vivo*. Proc Natl Acad Sci U S A.

[143] Drobny A., Boros F.A., Balta D., Prieto Huarcaya S., Caylioglu D., Qazi N. (2023). Reciprocal effects of alpha-synuclein aggregation and lysosomal homeostasis in synucleinopathy models. Transl Neurodegener.

[144] Lee J., Sung K.W., Bae E.J., Yoon D., Kim D., Lee J.S. (2023). Targeted degradation of *α*-synuclein aggregates in Parkinson's disease using the AUTOTAC technology. Mol Neurodegener.

[145] Thompson L., Barraud P., Andersson E., Kirik D., Bjorklund A. (2005). Identification of dopaminergic neurons of nigral and ventral tegmental area subtypes in grafts of fetal ventral mesencephalon based on cell morphology, protein expression, and efferent projections. J Neurosci.

[146] Ballion B., Bonnet M.L., Brot S., Gaillard A. (2025). Electrophysiological characterisation of intranigral-grafted hiPSC-derived dopaminergic neurons in a mouse model of Parkinson's disease. Stem Cell Res Ther.

[147] Hiller B.M., Marmion D.J., Thompson C.A., Elliott N.A., Federoff H., Brundin P. (2022). Optimizing maturity and dose of iPSC-derived dopamine progenitor cell therapy for Parkinson's disease. npj Regen Med.

[148] Takahashi R., Nakanishi E., Yamakado H., Sawamoto N., Takahashi J. (2025). Allogenic transplantation therapy of iPS cell-derived dopamine progenitors for Parkinson's disease—current status of the Kyoto Trial and future perspectives. Parkinsonism Relat Disord.

[149] Corti S., Bonjean R., Legier T., Rattier D., Melon C., Salin P. (2021). Enhanced differentiation of human induced pluripotent stem cells toward the midbrain dopaminergic neuron lineage through GLYPICAN-4 downregulation. Stem Cells Transl Med.

[150] Fedele S., Collo G., Behr K., Bischofberger J., Muller S., Kunath T. (2017). Expansion of human midbrain floor plate progenitors from induced pluripotent stem cells increases dopaminergic neuron differentiation potential. Sci Rep.

[151] Fujimori K., Matsumoto T., Kisa F., Hattori N., Okano H., Akamatsu W. (2017). Escape from pluripotency *via* inhibition of TGF-beta/BMP and activation of Wnt signaling accelerates differentiation and aging in hPSC progeny cells. Stem Cell Rep.

[152] Kriks S., Shim J.W., Piao J., Ganat Y.M., Wakeman D.R., Xie Z. (2011). Dopamine neurons derived from human ES cells efficiently engraft in animal models of Parkinson's disease. Nature.

[153] Hargus G., Cooper O., Deleidi M., Levy A., Lee K., Marlow E. (2010). Differentiated Parkinson patient-derived induced pluripotent stem cells grow in the adult rodent brain and reduce motor asymmetry in Parkinsonian rats. Proc Natl Acad Sci U S A.

[154] Rhee Y.H., Ko J.Y., Chang M.Y., Yi S.H., Kim D., Kim C.H. (2011). Protein-based human iPS cells efficiently generate functional dopamine neurons and can treat a rat model of Parkinson disease. J Clin Investig.

[155] Wang S., Zou C., Fu L., Wang B., An J., Song G. (2015). Autologous iPSC-derived dopamine neuron transplantation in a nonhuman primate Parkinson's disease model. Cell Discov.

[156] Morizane A., Kikuchi T., Hayashi T., Mizuma H., Takara S., Doi H. (2017). MHC matching improves engraftment of iPSC-derived neurons in non-human primates. Nat Commun.

[157] Gonzalez R., Garitaonandia I., Poustovoitov M., Abramihina T., McEntire C., Culp B. (2016). Neural stem cells derived from human parthenogenetic stem cells engraft and promote recovery in a nonhuman primate model of Parkinson's disease. Cell Transplant.

[158] Caiazzo M., Dell'Anno M.T., Dvoretskova E., Lazarevic D., Taverna S., Leo D. (2011). Direct generation of functional dopaminergic neurons from mouse and human fibroblasts. Nature.

[159] Chen Y., Kuang J., Niu Y., Zhu H., Chen X., So K.F. (2024). Multiple factors to assist human-derived induced pluripotent stem cells to efficiently differentiate into midbrain dopaminergic neurons. Neural Regen Res.

[160] Chlebanowska P., Sulkowski M., Skrzypek K., Tejchman A., Muszynska A., Noroozi R. (2020). Origin of the induced pluripotent stem cells affects their differentiation into dopaminergic neurons. Int J Mol Sci.

[161] Della Valle F., Thimma M.P., Caiazzo M., Pulcrano S., Celii M., Adroub S.A. (2020). Transdifferentiation of mouse embryonic fibroblasts into dopaminergic neurons reactivates LINE-1 repetitive elements. Stem Cell Rep.

[162] Kim J., Jeon J., Song B., Lee N., Ko S., Cha Y. (2022). Spotting-based differentiation of functional dopaminergic progenitors from human pluripotent stem cells. Nat Protoc.

[163] Lebedeva O.S., Sharova E.I., Grekhnev D.A., Skorodumova L.O., Kopylova I.V., Vassina E.M. (2023). An efficient 2D protocol for differentiation of iPSCs into mature postmitotic dopaminergic neurons: application for modeling Parkinson's disease. Int J Mol Sci.

[164] Li H., Jiang H., Li H., Li L., Yan Z., Feng J. (2022). Generation of human A9 dopaminergic pacemakers from induced pluripotent stem cells. Mol Psychiatr.

[165] Mahajani S., Raina A., Fokken C., Kugler S., Bahr M. (2019). Homogenous generation of dopaminergic neurons from multiple hiPSC lines by transient expression of transcription factors. Cell Death Dis.

[166] Maimaitili M., Chen M., Febbraro F., Ucuncu E., Kelly R., Niclis J.C. (2023). Enhanced production of mesencephalic dopaminergic neurons from lineage-restricted human undifferentiated stem cells. Nat Commun.

[167] Nakamura R., Nonaka R., Oyama G., Jo T., Kamo H., Nuermaimaiti M. (2023). A defined method for differentiating human iPSCs into midbrain dopaminergic progenitors that safely restore motor deficits in Parkinson's disease. Front Neurosci.

[168] Ng Y.H., Chanda S., Janas J.A., Yang N., Kokubu Y., Sudhof T.C. (2021). Efficient generation of dopaminergic induced neuronal cells with midbrain characteristics. Stem Cell Rep.

[169] Nolbrant S., Heuer A., Parmar M., Kirkeby A. (2017). Generation of high-purity human ventral midbrain dopaminergic progenitors for *in vitro* maturation and intracerebral transplantation. Nat Protoc.

[170] Pfisterer U., Kirkeby A., Torper O., Wood J., Nelander J., Dufour A. (2011). Direct conversion of human fibroblasts to dopaminergic neurons. Proc Natl Acad Sci U S A.

[171] Qin H., Zhao A.D., Sun M.L., Ma K., Fu X.B. (2020). Direct conversion of human fibroblasts into dopaminergic neuron-like cells using small molecules and protein factors. Milit Med Res.

[172] Ekrani S.T., Mahmoudi M., Haghmorad D., Kheder R.K., Hatami A., Esmaeili S.A. (2024). Manipulated mesenchymal stem cell therapy in the treatment of Parkinson's disease. Stem Cell Res Ther.

[173] Li J., Li N., Wei J., Feng C., Chen Y., Chen T. (2022). Genetically engineered mesenchymal stem cells with dopamine synthesis for Parkinson's disease in animal models. npj Parkinson's Dis.

[174] Fricova D., Korchak J.A., Zubair A.C. (2020). Challenges and translational considerations of mesenchymal stem/stromal cell therapy for Parkinson's disease. npj Regen Med.

[175] Barnabe G.F., Schwindt T.T., Calcagnotto M.E., Motta F.L., Martinez G., de Oliveira A.C. (2009). Chemically-induced RAT mesenchymal stem cells adopt molecular properties of neuronal-like cells but do not have basic neuronal functional properties. PLoS One.

[176] Chen D., Fu W., Zhuang W., Lv C., Li F., Wang X. (2017). Therapeutic effects of intranigral transplantation of mesenchymal stem cells in rat models of Parkinson's disease. J Neurosci Res.

[177] Thompson R., Casali C., Chan C. (2019). Forskolin and IBMX induce neural transdifferentiation of MSCs through downregulation of the NRSF. Sci Rep.

[178] Li Y., Chen J., Wang L., Zhang L., Lu M., Chopp M. (2001). Intracerebral transplantation of bone marrow stromal cells in a 1-methyl-4-phenyl-1,2,3,6-tetrahydropyridine mouse model of Parkinson's disease. Neurosci Lett.

[179] Bouchez G., Sensebe L., Vourc'h P., Garreau L., Bodard S., Rico A. (2008). Partial recovery of dopaminergic pathway after graft of adult mesenchymal stem cells in a rat model of Parkinson's disease. Neurochem Int.

[180] Schwerk A., Altschuler J., Roch M., Gossen M., Winter C., Berg J. (2015). Human adipose-derived mesenchymal stromal cells increase endogenous neurogenesis in the rat subventricular zone acutely after 6-hydroxydopamine lesioning. Cytotherapy.

[181] Delcroix G.J., Garbayo E., Sindji L., Thomas O., Vanpouille-Box C., Schiller P.C. (2011). The therapeutic potential of human multipotent mesenchymal stromal cells combined with pharmacologically active microcarriers transplanted in hemi-parkinsonian rats. Biomaterials.

[182] Rodriguez-Pallares J., Garcia-Garrote M., Parga J.A., Labandeira-Garcia J.L. (2021). Dose-dependent effect of mesenchymal stromal cells co-grafted with dopaminergic neurons in a Parkinson's disease rat model. J Cell Mol Med.

[183] Toledo-Aral J.J., Mendez-Ferrer S., Pardal R., Echevarria M., Lopez-Barneo J. (2003). Trophic restoration of the nigrostriatal dopaminergic pathway in long-term carotid body-grafted parkinsonian rats. J Neurosci.

[184] Villadiego J., Mendez-Ferrer S., Valdes-Sanchez T., Silos-Santiago I., Farinas I., Lopez-Barneo J. (2005). Selective glial cell line-derived neurotrophic factor production in adult dopaminergic carotid body cells in situ and after intrastriatal transplantation. J Neurosci.

[185] Luquin M.R., Montoro R.J., Guillen J., Saldise L., Insausti R., Del Rio J. (1999). Recovery of chronic parkinsonian monkeys by autotransplants of carotid body cell aggregates into putamen. Neuron.

[186] Munoz-Manchado A.B., Villadiego J., Suarez-Luna N., Bermejo-Navas A., Garrido-Gil P., Labandeira-Garcia J.L. (2013). Neuroprotective and reparative effects of carotid body grafts in a chronic MPTP model of Parkinson's disease. Neurobiol Aging.

[187] Espejo E.F., Montoro R.J., Armengol J.A., Lopez-Barneo J. (1998). Cellular and functional recovery of Parkinsonian rats after intrastriatal transplantation of carotid body cell aggregates. Neuron.

[188] Luquin M.R., Manrique M., Guillen J., Arbizu J., Ordonez C., Marcilla I. (2011). Enhanced GDNF expression in dopaminergic cells of monkeys grafted with carotid body cell aggregates. Brain Res.

[189] de Castro L.L., Lopes-Pacheco M., Weiss D.J., Cruz F.F., Rocco P.R.M. (2019). Current understanding of the immunosuppressive properties of mesenchymal stromal cells. J Mol Med (Berl).

[190] Liu S., Liu F., Zhou Y., Jin B., Sun Q., Guo S. (2020). Immunosuppressive property of MSCs mediated by cell surface receptors. Front Immunol.

[191] Unnisa A., Dua K., Kamal M.A. (2023). Mechanism of mesenchymal stem cells as a multitarget disease-modifying therapy for Parkinson's disease. Curr Neuropharmacol.

[192] Halliwell R.F., Salmanzadeh H., Coyne L., Cao W.S. (2021). An electrophysiological and pharmacological study of the properties of human iPSC-derived neurons for drug discovery. Cells.

[193] Rakovic A., Voss D., Vulinovic F., Meier B., Hellberg A.K., Nau C. (2022). Electrophysiological properties of induced pluripotent stem cell-derived midbrain dopaminergic neurons correlate with expression of tyrosine hydroxylase. Front Cell Neurosci.

[194] Zheng X., Han D., Liu W., Wang X., Pan N., Wang Y. (2023). Human iPSC-derived midbrain organoids functionally integrate into striatum circuits and restore motor function in a mouse model of Parkinson's disease. Theranostics.

[195] Tonnesen J., Parish C.L., Sorensen A.T., Andersson A., Lundberg C., Deisseroth K. (2011). Functional integration of grafted neural stem cell-derived dopaminergic neurons monitored by optogenetics in an *in vitro* Parkinson model. PLoS One.

[196] Steinbeck J.A., Choi S.J., Mrejeru A., Ganat Y., Deisseroth K., Sulzer D. (2015). Optogenetics enables functional analysis of human embryonic stem cell-derived grafts in a Parkinson's disease model. Nat Biotechnol.

[197] Jellinger K.A. (2015). Neuropathobiology of non-motor symptoms in Parkinson disease. J Neural Transm.

[198] Mazzotta G.M., Conte C. (2024). Alpha synuclein toxicity and non-motor Parkinson's. Cells.

[199] Grospe G.M., Baker P.M., Ragozzino M.E. (2018). Cognitive flexibility deficits following 6-OHDA lesions of the rat dorsomedial striatum. Neuroscience.

[200] Nguyen D., Alushaj E., Erb S., Ito R. (2019). Dissociative effects of dorsomedial striatum D1 and D2 receptor antagonism in the regulation of anxiety and learned approach-avoidance conflict decision-making. Neuropharmacology.

[201] Verharen J.P.H., Adan R.A.H., Vanderschuren L. (2019). Differential contributions of striatal dopamine D1 and D2 receptors to component processes of value-based decision making. Neuropsychopharmacology.

[202] Ferro M.M., Bellissimo M.I., Anselmo-Franci J.A., Angellucci M.E., Canteras N.S., Da Cunha C. (2005). Comparison of bilaterally 6-OHDA- and MPTP-lesioned rats as models of the early phase of Parkinson's disease: histological, neurochemical, motor and memory alterations. J Neurosci Methods.

[203] Tadaiesky M.T., Dombrowski P.A., Figueiredo C.P., Cargnin-Ferreira E., Da Cunha C., Takahashi R.N. (2008). Emotional, cognitive and neurochemical alterations in a premotor stage model of Parkinson's disease. Neuroscience.

[204] Santiago R.M., Barbieiro J., Lima M.M., Dombrowski P.A., Andreatini R., Vital M.A. (2010). Depressive-like behaviors alterations induced by intranigral MPTP, 6-OHDA, LPS and rotenone models of Parkinson's disease are predominantly associated with serotonin and dopamine. Prog Neuropsychopharmacol Biol Psychiatry.

[205] Loiodice S., Wing Young H., Rion B., Meot B., Montagne P., Denibaud A.S. (2019). Implication of nigral dopaminergic lesion and repeated l-dopa exposure in neuropsychiatric symptoms of Parkinson's disease. Behav Brain Res.

[206] Cools R., Stefanova E., Barker R.A., Robbins T.W., Owen A.M. (2002). Dopaminergic modulation of high-level cognition in Parkinson's disease: the role of the prefrontal cortex revealed by PET. Brain.

[207] McCoy B., Jahfari S., Engels G., Knapen T., Theeuwes J. (2019). Dopaminergic medication reduces striatal sensitivity to negative outcomes in Parkinson's disease. Brain.

[208] Wylie S.A., van Wouwe N.C., Godfrey S.G., Bissett P.G., Logan G.D., Kanoff K.E. (2018). Dopaminergic medication shifts the balance between going and stopping in Parkinson's disease. Neuropsychologia.

[209] Barone P., Poewe W., Albrecht S., Debieuvre C., Massey D., Rascol O. (2010). Pramipexole for the treatment of depressive symptoms in patients with Parkinson's disease: a randomised, double-blind, placebo-controlled trial. Lancet Neurol.

[210] Chiu C.H., Weng S.J., Yeh S.H., Jhao Y.T., Chang H.F., Huang W.S. (2022). Assessment of the anti-nociceptive effects of fetal ventral mesencephalic tissue allografts in a rat model of hemi-Parkinson's disease using fMRI. Front Aging Neurosci.

[211] Lelos M.J., Morgan R.J., Kelly C.M., Torres E.M., Rosser A.E., Dunnett S.B. (2016). Amelioration of non-motor dysfunctions after transplantation of human dopamine neurons in a model of Parkinson's disease. Exp Neurol.

[212] Sass K.J., Buchanan C.P., Westerveld M., Marek K.L., Farhi A., Robbins R.J. (1995). General cognitive ability following unilateral and bilateral fetal ventral mesencephalic tissue transplantation for treatment of Parkinson's disease. Arch Neurol.

[213] Trott C.T., Fahn S., Greene P., Dillon S., Winfield H., Winfield L. (2003). Cognition following bilateral implants of embryonic dopamine neurons in PD: a double blind study. Neurology.

[214] Boika A., Aleinikava N., Chyzhyk V., Zafranskaya M., Nizheharodava D., Ponomarev V. (2020). Mesenchymal stem cells in Parkinson's disease: motor and nonmotor symptoms in the early posttransplant period. Surg Neurol Int.

[215] Oomori Y., Nakaya K., Tanaka H., Iuchi H., Ishikawa K., Satoh Y. (1994). Immunohistochemical and histochemical evidence for the presence of noradrenaline, serotonin and gamma-aminobutyric acid in chief cells of the mouse carotid body. Cell Tissue Res.

[216] Riederer P., Strobel S., Nagatsu T., Watanabe H., Chen X., Loschmann P.A. (2025). Levodopa treatment: impacts and mechanisms throughout Parkinson's disease progression. J Neural Transm.

[217] Politis M., Oertel W.H., Wu K., Quinn N.P., Pogarell O., Brooks D.J. (2011). Graft-induced dyskinesias in Parkinson's disease: high striatal serotonin/dopamine transporter ratio. Mov Disord.

[218] Brinker D., Smilowska K., Paschen S., Antonini A., Moro E., Deuschl G. (2024). How to use the New European Academy of Neurology/Movement Disorder Society European Section Guideline for invasive therapies in Parkinson's disease. Mov Disord Clin Pract.

[219] Wang X., Chen M., Shen Y., Li Y., Li S., Xu Y. (2024). A longitudinal electrophysiological and behavior dataset for PD rat in response to deep brain stimulation. Sci Data.

[220] Oehrn C.R., Cernera S., Hammer L.H., Shcherbakova M., Yao J., Hahn A. (2024). Chronic adaptive deep brain stimulation *versus* conventional stimulation in Parkinson's disease: a blinded randomized feasibility trial. Nat Med.

[221] Szunyogh S., Carroll E., Wade-Martins R. (2025). Recent developments in gene therapy for Parkinson's disease. Mol Ther.

[222] Chen T.Y., Xu J., Tai C.H., Wen T.K., Hsu S.H. (2025). Biodegradable, electroconductive self-healing hydrogel based on polydopamine-coated polyurethane nano-crosslinker for Parkinson's disease therapy. Biomaterials.

[223] Guzman J.N., Sanchez-Padilla J., Wokosin D., Kondapalli J., Ilijic E., Schumacker P.T. (2010). Oxidant stress evoked by pacemaking in dopaminergic neurons is attenuated by DJ-1. Nature.

[224] Henrich M.T., Oertel W.H., Surmeier D.J., Geibl F.F. (2023). Mitochondrial dysfunction in Parkinson’s disease — a key disease hallmark with therapeutic potential. Mol Neurodegener.

[225] Tapias V. (2019). Editorial: mitochondrial dysfunction and neurodegeneration. Front Neurosci.

[226] Tapias V., McCoy J.L., Greenamyre J.T. (2019). Phenothiazine normalizes the NADH/NAD^+^ ratio, maintains mitochondrial integrity and protects the nigrostriatal dopamine system in a chronic rotenone model of Parkinson's disease. Redox Biol.

[227] Gibson G.E., Starkov A., Blass J.P., Ratan R.R., Beal M.F. (2010). Cause and consequence: mitochondrial dysfunction initiates and propagates neuronal dysfunction, neuronal death and behavioral abnormalities in age-associated neurodegenerative diseases. Biochim Biophys Acta.

[228] Li W., Fu Y., Halliday G.M., Sue C.M. (2021). PARK genes link mitochondrial dysfunction and alpha-synuclein pathology in sporadic Parkinson's disease. Front Cell Dev Biol.

[229] Emin D., Zhang Y.P., Lobanova E., Miller A., Li X., Xia Z. (2022). Small soluble alpha-synuclein aggregates are the toxic species in Parkinson's disease. Nat Commun.

[230] Mann V.M., Cooper J.M., Krige D., Daniel S.E., Schapira A.H., Marsden C.D. (1992). Brain, skeletal muscle and platelet homogenate mitochondrial function in Parkinson's disease. Brain.

[231] Parker W.D., Parks J.K., Swerdlow R.H. (2008). Complex I deficiency in Parkinson's disease frontal cortex. Brain Res.

[232] Schapira A.H., Cooper J.M., Dexter D., Jenner P., Clark J.B., Marsden C.D. (1989). Mitochondrial complex I deficiency in Parkinson's disease. Lancet.

[233] Cardellach F., Marti M.J., Fernandez-Sola J., Marin C., Hoek J.B., Tolosa E. (1993). Mitochondrial respiratory chain activity in skeletal muscle from patients with Parkinson's disease. Neurology.

[234] Mak S.K., Tewari D., Tetrud J.W., Langston J.W., Schule B. (2011). Mitochondrial dysfunction in skin fibroblasts from a Parkinson's disease patient with an alpha-synuclein triplication. J Parkinsons Dis.

[235] Yoshino H., Nakagawa-Hattori Y., Kondo T., Mizuno Y. (1992). Mitochondrial complex I and II activities of lymphocytes and platelets in Parkinson's disease. J Neural Transm Park Dis Dement Sect.

[236] Xu X., Pang Y., Fan X. (2025). Mitochondria in oxidative stress, inflammation and aging: from mechanisms to therapeutic advances. Signal Transduct Targeted Ther.

[237] Cannon J.R., Tapias V., Na H.M., Honick A.S., Drolet R.E., Greenamyre J.T. (2009). A highly reproducible rotenone model of Parkinson's disease. Neurobiol Dis.

[238] Wu D.C., Teismann P., Tieu K., Vila M., Jackson-Lewis V., Ischiropoulos H. (2003). NADPH oxidase mediates oxidative stress in the 1-methyl-4-phenyl-1,2,3,6-tetrahydropyridine model of Parkinson's disease. Proc Natl Acad Sci U S A.

[239] Chen W., Zhao H., Li Y. (2023). Mitochondrial dynamics in health and disease: mechanisms and potential targets. Signal Transduct Targeted Ther.

[240] Cipolat S., Martins de Brito O., Dal Zilio B., Scorrano L. (2004). OPA1 requires mitofusin 1 to promote mitochondrial fusion. Proc Natl Acad Sci U S A.

[241] Kalia R., Wang R.Y., Yusuf A., Thomas P.V., Agard D.A., Shaw J.M. (2018). Structural basis of mitochondrial receptor binding and constriction by DRP1. Nature.

[242] Itoh K., Nakamura K., Iijima M., Sesaki H. (2013). Mitochondrial dynamics in neurodegeneration. Trends Cell Biol.

[243] Willems P.H., Rossignol R., Dieteren C.E., Murphy M.P., Koopman W.J. (2015). Redox homeostasis and mitochondrial dynamics. Cell Metab.

[244] Li Z., Okamoto K., Hayashi Y., Sheng M. (2004). The importance of dendritic mitochondria in the morphogenesis and plasticity of spines and synapses. Cell.

[245] Fang D., Yan S., Yu Q., Chen D., Yan S.S. (2016). Mfn2 is required for mitochondrial development and synapse formation in human induced pluripotent stem cells/hiPSC derived cortical neurons. Sci Rep.

[246] Verstreken P., Ly C.V., Venken K.J., Koh T.W., Zhou Y., Bellen H.J. (2005). Synaptic mitochondria are critical for mobilization of reserve pool vesicles at Drosophila neuromuscular junctions. Neuron.

[247] Zilocchi M., Finzi G., Lualdi M., Sessa F., Fasano M., Alberio T. (2018). Mitochondrial alterations in Parkinson's disease human samples and cellular models. Neurochem Int.

[248] Liao C., Ashley N., Diot A., Morten K., Phadwal K., Williams A. (2017). Dysregulated mitophagy and mitochondrial organization in optic atrophy due to OPA1 mutations. Neurology.

[249] Lee S., Sterky F.H., Mourier A., Terzioglu M., Cullheim S., Olson L. (2012). Mitofusin 2 is necessary for striatal axonal projections of midbrain dopamine neurons. Hum Mol Genet.

[250] Pham A.H., Meng S., Chu Q.N., Chan D.C. (2012). Loss of Mfn2 results in progressive, retrograde degeneration of dopaminergic neurons in the nigrostriatal circuit. Hum Mol Genet.

[251] Feng S.T., Wang Z.Z., Yuan Y.H., Wang X.L., Guo Z.Y., Hu J.H. (2021). Inhibition of dynamin-related protein 1 ameliorates the mitochondrial ultrastructure *via* PINK1 and Parkin in the mice model of Parkinson's disease. Eur J Pharmacol.

[252] Cason S.E., Holzbaur E.L.F. (2022). Selective motor activation in organelle transport along axons. Nat Rev Mol Cell Biol.

[253] Kaan H.Y., Hackney D.D., Kozielski F. (2011). The structure of the kinesin-1 motor-tail complex reveals the mechanism of autoinhibition. Science.

[254] Canty J.T., Yildiz A. (2020). Activation and regulation of cytoplasmic dynein. Trends Biochem Sci.

[255] Stowers R.S., Megeath L.J., Gorska-Andrzejak J., Meinertzhagen I.A., Schwarz T.L. (2002). Axonal transport of mitochondria to synapses depends on milton, a novel *Drosophila* protein. Neuron.

[256] Russo G.J., Louie K., Wellington A., Macleod G.T., Hu F., Panchumarthi S. (2009). *Drosophila* Miro is required for both anterograde and retrograde axonal mitochondrial transport. J Neurosci.

[257] Chen Y.M., Gerwin C., Sheng Z.H. (2009). Dynein light chain LC8 regulates syntaphilin-mediated mitochondrial docking in axons. J Neurosci.

[258] Chu Y., Morfini G.A., Langhamer L.B., He Y., Brady S.T., Kordower J.H. (2012). Alterations in axonal transport motor proteins in sporadic and experimental Parkinson's disease. Brain.

[259] Liang W., Sagar S., Ravindran R., Najor R.H., Quiles J.M., Chi L. (2023). Mitochondria are secreted in extracellular vesicles when lysosomal function is impaired. Nat Commun.

[260] Geisler S., Holmstrom K.M., Skujat D., Fiesel F.C., Rothfuss O.C., Kahle P.J. (2010). PINK1/Parkin-mediated mitophagy is dependent on VDAC1 and p62/SQSTM1. Nat Cell Biol.

[261] Ziviani E., Tao R.N., Whitworth A.J. (2010). Drosophila parkin requires PINK1 for mitochondrial translocation and ubiquitinates mitofusin. Proc Natl Acad Sci U S A.

[262] Shin J.H., Ko H.S., Kang H., Lee Y., Lee Y.I., Pletinkova O. (2011). PARIS (ZNF746) repression of PGC-1alpha contributes to neurodegeneration in Parkinson's disease. Cell.

[263] Cornelissen T., Vilain S., Vints K., Gounko N., Verstreken P., Vandenberghe W. (2018). Deficiency of parkin and PINK1 impairs age-dependent mitophagy in *Drosophila*. eLife.

[264] McWilliams T.G., Prescott A.R., Montava-Garriga L., Ball G., Singh F., Barini E. (2018). Basal mitophagy occurs independently of PINK1 in mouse tissues of high metabolic demand. Cell Metab.

[265] Ashrafi G., Schlehe J.S., LaVoie M.J., Schwarz T.L. (2014). Mitophagy of damaged mitochondria occurs locally in distal neuronal axons and requires PINK1 and Parkin. J Cell Biol.

[266] Devireddy S., Liu A., Lampe T., Hollenbeck P.J. (2015). The organization of mitochondrial quality control and life cycle in the nervous system *in vivo* in the absence of PINK1. J Neurosci.

[267] Stevens D.A., Lee Y., Kang H.C., Lee B.D., Lee Y.I., Bower A. (2015). Parkin loss leads to PARIS-dependent declines in mitochondrial mass and respiration. Proc Natl Acad Sci U S A.

[268] Chung K.K., Thomas B., Li X., Pletnikova O., Troncoso J.C., Marsh L. (2004). *S*-Nitrosylation of parkin regulates ubiquitination and compromises parkin's protective function. Science.

[269] Hoepken H.H., Gispert S., Morales B., Wingerter O., Del Turco D., Mulsch A. (2007). Mitochondrial dysfunction, peroxidation damage and changes in glutathione metabolism in PARK6. Neurobiol Dis.

[270] Seibler P., Graziotto J., Jeong H., Simunovic F., Klein C., Krainc D. (2011). Mitochondrial Parkin recruitment is impaired in neurons derived from mutant PINK1 induced pluripotent stem cells. J Neurosci.

[271] Gandhi S., Muqit M.M., Stanyer L., Healy D.G., Abou-Sleiman P.M., Hargreaves I. (2006). PINK1 protein in normal human brain and Parkinson's disease. Brain.

[272] Hallett P.J., Cooper O., Sadi D., Robertson H., Mendez I., Isacson O. (2014). Long-term health of dopaminergic neuron transplants in Parkinson's disease patients. Cell Rep.

[273] Liu K., Ji K., Guo L., Wu W., Lu H., Shan P. (2014). Mesenchymal stem cells rescue injured endothelial cells in an *in vitro* ischemia–reperfusion model *via* tunneling nanotube like structure-mediated mitochondrial transfer. Microvasc Res.

[274] Hessvik N.P., Llorente A. (2018). Current knowledge on exosome biogenesis and release. Cell Mol Life Sci.

[275] Salzman L.F., Goldstein R.H., Atkins R., Babigian H. (1966). Conceptual thinking in psychiatric patients. Arch Gen Psychiatry.

[276] Pinnell J.R., Cui M., Tieu K. (2021). Exosomes in Parkinson disease. J Neurochem.

[277] Heris R.M., Shirvaliloo M., Abbaspour-Aghdam S., Hazrati A., Shariati A., Youshanlouei H.R. (2022). The potential use of mesenchymal stem cells and their exosomes in Parkinson's disease treatment. Stem Cell Res Ther.

[278] Chmielarz P., Konovalova J., Najam S.S., Alter H., Piepponen T.P., Erfle H. (2017). Dicer and microRNAs protect adult dopamine neurons. Cell Death Dis.

[279] Pang X., Hogan E.M., Casserly A., Gao G., Gardner P.D., Tapper A.R. (2014). Dicer expression is essential for adult midbrain dopaminergic neuron maintenance and survival. Mol Cell Neurosci.

[280] Guo M., Wang J., Zhao Y., Feng Y., Han S., Dong Q. (2020). Microglial exosomes facilitate alpha-synuclein transmission in Parkinson's disease. Brain.

[281] Stuendl A., Kunadt M., Kruse N., Bartels C., Moebius W., Danzer K.M. (2016). Induction of alpha-synuclein aggregate formation by CSF exosomes from patients with Parkinson's disease and dementia with Lewy bodies. Brain.

[282] Spencer B., Kim C., Gonzalez T., Bisquertt A., Patrick C., Rockenstein E. (2016). alpha-Synuclein interferes with the ESCRT-III complex contributing to the pathogenesis of Lewy body disease. Hum Mol Genet.

[283] Xia Y., Zhang G., Han C., Ma K., Guo X., Wan F. (2019). Microglia as modulators of exosomal alpha-synuclein transmission. Cell Death Dis.

[284] Tsunemi T., Ishiguro Y., Yoroisaka A., Valdez C., Miyamoto K., Ishikawa K. (2020). Astrocytes protect human dopaminergic neurons from alpha-synuclein accumulation and propagation. J Neurosci.

[285] Fraser K.B., Rawlins A.B., Clark R.G., Alcalay R.N., Standaert D.G., Liu N. (2016). Ser(P)-1292 LRRK2 in urinary exosomes is elevated in idiopathic Parkinson's disease. Mov Disord.

[286] Zou J., Guo Y., Wei L., Yu F., Yu B., Xu A. (2020). Long noncoding RNA POU3F3 and alpha-Synuclein in plasma L1CAM exosomes combined with beta-glucocerebrosidase activity: potential predictors of Parkinson's disease. Neurotherapeutics.

[287] McMillan K.J., Murray T.K., Bengoa-Vergniory N., Cordero-Llana O., Cooper J., Buckley A. (2017). Loss of microRNA-7 regulation leads to alpha-synuclein accumulation and dopaminergic neuronal loss *in vivo*. Mol Ther.

[288] Zhang J., Zhao M., Yan R., Liu J., Maddila S., Junn E. (2021). MicroRNA-7 protects against neurodegeneration induced by alpha-synuclein preformed fibrils in the mouse brain. Neurotherapeutics.

[289] Chen Y., Gao C., Sun Q., Pan H., Huang P., Ding J. (2017). MicroRNA-4639 is a regulator of DJ-1 expression and a potential early diagnostic marker for Parkinson's disease. Front Aging Neurosci.

[290] He L., Chen Y., Lin S., Shen R., Pan H., Zhou Y. (2023). Regulation of Hsa-miR-4639-5p expression and its potential role in the pathogenesis of Parkinson's disease. Aging Cell.

[291] Jiang Y., Liu J., Chen L., Jin Y., Zhang G., Lin Z. (2019). Serum secreted miR-137-containing exosomes affects oxidative stress of neurons by regulating OXR1 in Parkinson's disease. Brain Res.

[292] Zhang Z.X., Zhou Y.J., Gu P., Zhao W., Chen H.X., Wu R.Y. (2023). Exosomes derived from human umbilical cord mesenchymal stem cells alleviate Parkinson's disease and neuronal damage through inhibition of microglia. Neural Regen Res.

[293] Berg J., Roch M., Altschuler J., Winter C., Schwerk A., Kurtz A. (2015). Human adipose-derived mesenchymal stem cells improve motor functions and are neuroprotective in the 6-hydroxydopamine-rat model for Parkinson's disease when cultured in monolayer cultures but suppress hippocampal neurogenesis and hippocampal memory function when cultured in spheroids. Stem Cell Rev Rep.

[294] Jarmalaviciute A., Tunaitis V., Pivoraite U., Venalis A., Pivoriunas A. (2015). Exosomes from dental pulp stem cells rescue human dopaminergic neurons from 6-hydroxy-dopamine-induced apoptosis. Cytotherapy.

[295] Xue C., Li X., Ba L., Zhang M., Yang Y., Gao Y. (2021). MSC-derived exosomes can enhance the angiogenesis of human brain MECs and show therapeutic potential in a mouse model of Parkinson's disease. Aging Dis.

[296] Chen H.X., Liang F.C., Gu P., Xu B.L., Xu H.J., Wang W.T. (2020). Exosomes derived from mesenchymal stem cells repair a Parkinson's disease model by inducing autophagy. Cell Death Dis.

[297] Chen H.X., Xu H.J., Zhang W., Luo Z.Y., Zhang Z.X., Shi H.H. (2025). HucMSCs-derived exosomes protect against 6-hydroxydopamineinduced Parkinson's disease in rats by inhibiting Caspase-3 expression and suppressing apoptosis. Curr Stem Cell Res Ther.

[298] He S., Wang Q., Chen L., He Y.J., Wang X., Qu S. (2023). miR-100a-5p-enriched exosomes derived from mesenchymal stem cells enhance the anti-oxidant effect in a Parkinson's disease model *via* regulation of Nox4/ROS/Nrf2 signaling. J Transl Med.

[299] Kim J., Inoue K., Ishii J., Vanti W.B., Voronov S.V., Murchison E. (2007). A microRNA feedback circuit in midbrain dopamine neurons. Science.

[300] Li Q., Wang Z., Xing H., Wang Y., Guo Y. (2021). Exosomes derived from miR-188-3p-modified adipose-derived mesenchymal stem cells protect Parkinson's disease. Mol Ther Nucleic Acids.

[301] Leggio L., Vivarelli S., L'Episcopo F., Tirolo C., Caniglia S., Testa N. (2017). microRNAs in Parkinson's disease: from pathogenesis to novel diagnostic and therapeutic approaches. Int J Mol Sci.

[302] Park T.Y., Jeon J., Cha Y., Kim K.S. (2024). Past, present, and future of cell replacement therapy for Parkinson's disease: a novel emphasis on host immune responses. Cell Res.

[303] Mayer E., Fawcett J.W., Dunnett S.B. (1993). Basic fibroblast growth factor promotes the survival of embryonic ventral mesencephalic dopaminergic neurons--II. Effects on nigral transplants *in vivo*. Neuroscience.

[304] Takayama H., Ray J., Raymon H.K., Baird A., Hogg J., Fisher L.J. (1995). Basic fibroblast growth factor increases dopaminergic graft survival and function in a rat model of Parkinson's disease. Nat Med.

[305] Zeng B.Y., Jenner P., Marsden C.D. (1996). Altered motor function and graft survival produced by basic fibroblast growth factor in rats with 6-OHDA lesions and fetal ventral mesencephalic grafts are associated with glial proliferation. Exp Neurol.

[306] Redmond D.E., McEntire C.R., Kingsbery J.P., Leranth C., Elsworth J.D., Bjugstad K.B. (2013). Comparison of fetal mesencephalic grafts, AAV-delivered GDNF, and both combined in an MPTP-induced nonhuman primate Parkinson's model. Mol Ther.

[307] Tatard V.M., Sindji L., Branton J.G., Aubert-Pouessel A., Colleau J., Benoit J.P. (2007). Pharmacologically active microcarriers releasing glial cell line - derived neurotrophic factor: survival and differentiation of embryonic dopaminergic neurons after grafting in hemiparkinsonian rats. Biomaterials.

[308] Novosadova E.V., Nenasheva V.V., Makarova I.V., Dolotov O.V., Inozemtseva L.S., Arsenyeva E.L. (2020). Parkinson's disease-associated changes in the expression of neurotrophic factors and their receptors upon neuronal differentiation of human induced pluripotent stem cells. J Mol Neurosci.

[309] Moriarty N., Gantner C.W., Hunt C.P.J., Ermine C.M., Frausin S., Viventi S. (2022). A combined cell and gene therapy approach for homotopic reconstruction of midbrain dopamine pathways using human pluripotent stem cells. Cell Stem Cell.

[310] Wakeman D.R., Redmond D.E., Dodiya H.B., Sladek J.R., Leranth C., Teng Y.D. (2014). Human neural stem cells survive long term in the midbrain of dopamine-depleted monkeys after GDNF overexpression and project neurites toward an appropriate target. Stem Cells Transl Med.

[311] Jiang Z., Wang J., Sun G., Feng M. (2022). BDNF-modified human umbilical cord mesenchymal stem cells-derived dopaminergic-like neurons improve rotation behavior of Parkinson's disease rats through neuroprotection and anti-neuroinflammation. Mol Cell Neurosci.

[312] Jinfeng L., Yunliang W., Xinshan L., Shanshan W., Chunyang X., Peng X. (2016). The effect of MSCs derived from the human umbilical cord transduced by fibroblast growth factor-20 on Parkinson's disease. Stem Cell Int.

[313] Xiong N., Zhang Z., Huang J., Chen C., Zhang Z., Jia M. (2011). VEGF-expressing human umbilical cord mesenchymal stem cells, an improved therapy strategy for Parkinson's disease. Gene Ther.

[314] Jiaming M., Niu C. (2015). Comparing neuroprotective effects of CDNF-expressing bone marrow derived mesenchymal stem cells *via* differing routes of administration utilizing an *in vivo* model of Parkinson's disease. Neurol Sci.

[315] Somoza R., Juri C., Baes M., Wyneken U., Rubio F.J. (2010). Intranigral transplantation of epigenetically induced BDNF-secreting human mesenchymal stem cells: implications for cell-based therapies in Parkinson's disease. Biol Blood Marrow Transplant.

[316] Shi D., Chen G., Lv L., Li L., Wei D., Gu P. (2011). The effect of lentivirus-mediated TH and GDNF genetic engineering mesenchymal stem cells on Parkinson's disease rat model. Neurol Sci.

[317] Xiong N., Yang H., Liu L., Xiong J., Zhang Z., Zhang X. (2013). bFGF promotes the differentiation and effectiveness of human bone marrow mesenchymal stem cells in a rotenone model for Parkinson's disease. Environ Toxicol Pharmacol.

[318] Villadiego J., Garcia-Swinburn R., Garcia-Gonzalez D., Lebron-Galan R., Murcia-Belmonte V., Garcia-Roldan E. (2023). Extracellular matrix protein anosmin-1 overexpression alters dopaminergic phenotype in the CNS and the PNS with no pathogenic consequences in a MPTP model of Parkinson's disease. Brain Struct Funct.

[319] Emborg M.E., Metzger J.M., D'Amour K., Colwell J.C., Neumann L.C., Zhang A. (2025). Advantages and challenges of using allogeneic *vs*. autologous sources for neuronal cell replacement in Parkinson's disease: insights from non-human primate studies. Brain Res Bull.

[320] Qarin S., Howlett S.K., Jones J.L., Barker R.A. (2021). The immunogenicity of midbrain dopaminergic neurons and the implications for neural grafting trials in Parkinson's disease. Neuronal Signal.

[321] Bashor C.J., Hilton I.B., Bandukwala H., Smith D.M., Veiseh O. (2022). Engineering the next generation of cell-based therapeutics. Nat Rev Drug Discov.

[322] Cerneckis J., Cai H., Shi Y. (2024). Induced pluripotent stem cells (iPSCs): molecular mechanisms of induction and applications. Signal Transduct Targeted Ther.

[323] Kim K., Doi A., Wen B., Ng K., Zhao R., Cahan P. (2010). Epigenetic memory in induced pluripotent stem cells. Nature.

[324] Ohi Y., Qin H., Hong C., Blouin L., Polo J.M., Guo T. (2011). Incomplete DNA methylation underlies a transcriptional memory of somatic cells in human iPS cells. Nat Cell Biol.

[325] Gravina A., Tediashvili G., Zheng Y., Iwabuchi K.A., Peyrot S.M., Roodsari S.Z. (2023). Synthetic immune checkpoint engagers protect HLA-deficient iPSCs and derivatives from innate immune cell cytotoxicity. Cell Stem Cell.

[326] Meissner T.B., Schulze H.S., Dale S.M. (2022). Immune editing: overcoming immune barriers in stem cell transplantation. Curr Stem Cell Rep.

[327] Lazarov N.E., Atanasova D.Y. (2023). Carotid body dysfunction and mechanisms of disease. Adv Anat Embryol Cell Biol.

[328] Kurogoushi R., Hasegawa T., Akazawa Y., Iwata K., Sugimoto A., Yamaguchi-Ueda K. (2021). Fibroblast growth factor 2 suppresses the expression of C–C motif chemokine 11 through the c-Jun N-terminal kinase pathway in human dental pulp-derived mesenchymal stem cells. Exp Ther Med.

[329] Zhuo Y., Li W.S., Lu W., Li X., Ge L.T., Huang Y. (2024). TGF-beta1 mediates hypoxia-preconditioned olfactory mucosa mesenchymal stem cells improved neural functional recovery in Parkinson's disease models and patients. Milit Med Res.

[330] Zita G., Summer K. (1967). [Scintigraphy in the diagnosis of bone diseases]. Wien Med Wochenschr.

